# Supplementary material for: Measuring the effects of a nurse-led intervention on frailty status of older people living in the community in Ethiopia: A protocol for a quasi-experimental study
Source: PLoS One. 2024 Jan 19;19(1):e0296166. doi: 10.1371/journal.pone.0296166 (PMC10798498; doi:10.1371/journal.pone.0296166)
Supplement: S1 Checklist — (DOC) [file pone.0296166.s001.doc]

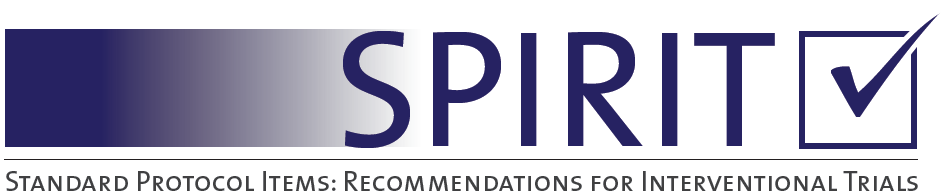


SPIRIT 2013 Checklist: Recommended items to address in a clinical trial protocol and related documents*

| Section/item | ItemNo | Description |
| --- | --- | --- |
| **Administrative information** | | |
| Title | 1 | Measuring the Effects of a Nurse-led Intervention on Frailty Status of Older People Living in the Community in Ethiopia: A Protocol for a Quasi-experimental Study |
| Trial registration | 2a | NCT05754398. |
|  | 2b | ClinicalTrial.gov registry |
| Protocol version | 3 | Version 3 |
| Funding | 4 | Financial support as a stipend by the University of Wollongong |
| Roles and responsibilities | 5a | University of Wollongong |
|  | 5b | Ayele Semachew Kasa as a contact and responsible party,  ask255@uowmail.edu.au |
|  | 5c | The project will be followed and supported technically by senior experts, professors, and supervisors from the University of Wollongong. |
|  | 5d | A group of supervisors will oversee the progress of the study. |
| Introduction |  |  |
| Background and rationale | 6a | Considering the importance of the health of older persons and the growing increase of the ageing population in Ethiopia, understanding frailty status and the effect of a nurse-led intervention on older persons with frailty can help in designing inclusive health promotion programs for older persons in Sub-Sharan Africa. The intervention will be designed based on the Integral Conceptual Model of Frailty (ICMF) framework. This framework beliefs that physical, psychological, and social domains are key components to ensure the health of frail older persons.  Studies from developed countries have recognised the importance of frailty in older persons and have developed a number of frailty interventions with positive outcomes in community settings. These studies suggest that further researches are needed (Marcus-Varwijk et al. 2020) to evaluate the effectiveness of additional nurse-led interventions in other contexts and settings (Markle-Reid, Browne, and Gafni 2013; Song and Boo 2022) with a relatively longer follow-up (Ha and Park 2020). A study conducted by Marker et al (Markle-Reid et al. 2006) also showed that proactively providing home-based care health promotion to frail older persons would improve QOL and reduce frailty status. |
|  | 6b | There is no comparator group. |
| Objectives | 7 | The nurse-led intervention decreases the frailty including the physical, social, and psychological domains among the older persons living in the community in Bahir Dar, Ethiopia.  The nurse-led intervention improves the quality of life among the older persons living in the community in Bahir Dar, Ethiopia. |
| Trial design | 8 | A pre-, post-, and follow-up single group quasi-experimental study design will be adopted to examine the effect of a nurse-led intervention on frailty among older persons living in Bahir Dar City, Ethiopia. This study will adhere to the Transparent Reporting of Evaluations with Nonrandomized Designs (TREND) guidelines (Des Jarlais et 2004). |

| Methods: Participants, interventions, and outcomes | | |
| --- | --- | --- |
| Study setting | 9 | The study will be conducted in a region found in Sub-Saharan Africa, Bahir Dar, Ethiopia. Bahir Dar is the capital city for the regional state of Amhara in Ethiopia. Based on a survey conducted by Bahir Dar City Labour and Social Affairs Administration Office in 2018 revealed that there were over 3,300 older persons in Bahir Dar City administration (Birhanie et al. 2021).  One sub-city from Bahir Dar will be selected using the lottery method. The list of older persons in the selected sub-city will be selected from the household’s registration of the city’s administration health office or from the health post. Initially, study participants will be contacted by the Community Health Workers (CHWs) through home-to-home visit for explaining the aim of the study, getting consent, screening frailty status, and their interest to participate in the intervention/study. CHWs are registered nurses working in the same area where study participants residing and work at the health post and home visiting. |
| Eligibility criteria | 10 | The definition of old age depends on various countries’ settings to determine the old age cutoff point (Jemal et al. 2021). In Ethiopia, the cut points of old age started at 60 years (Federal Negarit Gazeta of the Federal Democratic Republic Of Ethiopia 2011; HelpAge International 2013). Hence, older persons aged 60 years or above, whose frailty score ≥ 5 as measured by the Tilburg Frailty Indicator (TFI) and residing in Bahir Dar, Ethiopia will be included in the study. Participants will be excluded if they are unable to communicate, have cognitive impairment, are bed-redden, not living at home, have been hospitalised with a known psychiatric problem within the past six months, and will not remain in the selected area during the study period. |
| Interventions | 11a | The intervention comprises six independent and interconnected educational training sessions on:  Ageing and age-related changes, healthy nutrition, physical activity, mental health, social interaction and support and overall discussion.  On each session, the intervention providers will describe the training with learning objectives prior to the training, ask leading questions of the session and at the end of each session study participants will be given a simple take-home message. Moreover, the study participants will have the opportunity to reflect ideas, ask questions and discuss with the intervention providers. Each of the six components will be offered each month for six consecutive months. Each session will last approximately from 30 to 40 minutes. All the six sessions will be delivered through a face-to-face approach. |
|  | 11b | Study participants will be provided an exercise safety protocol that will guide them what to do if they experience discomforts while engaging in the activity. Moreover, they feel uncomfortable symptoms during the intervention sessions or during the survey, they have the full right to stop their participation. |
|  | 11c | To improve adherence and reduce lost to follow-up (LTFU), participants will be encouraged and reminded by phone to attend upcoming sessions. In the intervention period, there will be also a fort nightly 5 to 10-minute follow-up phone call with study participants to get feedback on their training. |
|  | 11d | A home-based intervention by two Community Health Workers (CHWs) with close supervision and support by a PhD candidate in nursing. |
| Outcomes | 12 | Level of frailty as measured by the Tilburg Frailty Indicator Amharic Version (TFI-AM) is the primary outcome of the study. Whereas nutritional status as measured by the mini-nutritional assessment (MNA) tool, depression as measured by the Geriatric Depression Scale-15 (GDS-15), activity of daily living as measured by the Katz-ADL and quality of life as measured by (WHOQOL-BREEF) were the secondary outcome of the study. |
| Participant timeline | 13 | After confirming study participant eligibility and willingness to join the study, a baseline questionnaire will be administered prior to the start of the nurse-led intervention. At the end, study participants who received all the nurse-led intervention sessions will be included in the final analysis to determine the effectiveness of the nurse-led intervention in reducing frailty among older persons in Ethiopia (Figure 1).  Assessed for eligibility/screening (n=?) Required sample (n=68)  Baseline data (n=TBD)  Study participants who provided consent for participation (n=TBD)  Follow-up status (n=TBD), with reason. Eg. LTFU (Transfer out, accident, death etc.)  Study participants for analysis (n=TBD)  Eligibility checks  Contacted at baseline for consent  Baseline data collection  Follow-up  End line  Figure 1: The flow chart of educational intervention of the study.  TBD: To be determined |
| Sample size | 14 | The sample size is calculated using a priori computation of sample size using G* Power version 3.1.9.4 (Faul F, Erdfelder E, Buchner A 2009) with assumption of a two-tailed test with an alpha value of 0.05, effect size (f) of 0.5, and a power of 0.95 revealed that 57 participants are required. By considering a 10 to 20% (Ha and Park 2020; Markle-Reid et al. 2013) withdrawal rate during the intervention, at least 68 study participants will be required. |
| Recruitment | 15 | Study participants will be contacted by the Community Health Workers (CHWs) through home-to-home visit for explaining the aim of the study, getting consent, screening frailty status, and their interest to participate in the intervention/study. CHWs are registered nurses working in the same area where study participants residing and work at the health post and home visiting. |
| **Methods: Assignment of interventions (for controlled trials)** | | |
| Allocation: |  |  |
| Sequence generation | 16a | As the study is a single group quasi-experimental study design, allocation of the study participants into different groups is not applicable. |
| Allocation concealment mechanism | 16b | A poster that contains the aim of the study, eligibility criteria, criteria required to participate and benefits of participating in the study will be distributed through health posts and community gatherings using the local language. The list of older persons in the selected sub-city will be obtained from the household’s registration with the city’s administration health office. After communicated by the Community Health Workers (CHWs) through home-to-home visits for explaining the aim of the study, screen frailty status, and get consent, and interest in participating in the intervention. |
| Implementation | 16c | As mentioned in 16a above, there will be a single group of study participants who will receive the treatment. The enrolment and assignment of the treatment will be made by the CHWs. |
| Blinding (masking) | 17a | Though the there is a single group of intervention groups, the outcome assessors will not be able to know what interventions were delivered to the intervention groups. |
|  | 17b | There will not be blinding. |
| **Methods: Data collection, management, and analysis** | | |
| Data collection methods | 18a | The data will be collected through a face-to-face administered structured survey questionnaire, and anthropometrical physical measurements. To reduce assessor bias, CHWs will not be involved in the data collection process. Hence, two professional nurses from Bahir Dar city will be recruited for data collection. The data collectors will not be involved in providing the intervention. They will be trained for two days about measurement tools, and how the study participants will be approached ethically. To determine the effect of a nurse-led intervention on frailty status among older persons, data will be collected at baseline (before intervention) (T0), immediately after the intervention (T1) and at twelfth week of the intervention (T2) (Ha and Park 2020; Haider et al. 2017; Lee et al. 2018). The primary and secondary outcome measures will be measured using validated tools. |
|  | 18b | Strategies to promote study participants’ retention in the intervention is planned as indicated in 11c. |
| Data management | 19 | The data will be collected as hard copy print outs and kept in a locked filing cabinet for a period of five years by the PhD candidate. Collected data will be entered into EpiData software manager and individual paper ID number coding will be given instead of personal identifiable information. Furthermore, a securely password protected USB drive will be used to store the data set and statistical analysis outputs. |
| Statistical methods | 20a | Fully completed and cleaned questionnaires will be entered in EpiData Manager software and exported to the IBM SPSS 26.0 (IBM Corp., Armonk, NY, USA) for analyses. To understand the correlates of frailty among older persons, Pearson correlation analysis will be utilised. Numerical and categorical data will be summarized as mean (±*SD*) and frequencies, respectively. Normality of the data will be checked. To measure the effect of a nurse-led intervention on the level of frailty among older persons Fisher's exact test, paired sample *t* test, and a generalised linear model (GLM) using repeated measures ANOVA will be used. If the data is not normal, their nonparametric equivalents (Wilcoxon and Friedman tests) will be applied. Statistical significances will be set at *p*-values < 0.5. |
|  | 20b | No other additional method of analysis will be employed. |
|  | 20c | To handle any missing data, a series mean imputation and linear interpolation imputation, will be applied in SPSS. |
| **Methods: Monitoring** | | |
| Data monitoring | 21a | The study progress and monitoring of activities related to the research will be led by the principal investigator and support and guidance will be received from supervisors of the study. Moreover, the ethical conduct of the study will be monitored by the Institutional Review Boards (IRB). However, for this particular study, there is no established data monitoring committee. |
|  | 21b | In this study, an analysis of data before data collection will not be conducted. If there is any, the final decision to terminate the trial will rely on the principal investigator and the stud supervision teams. A notification of the decision will be made to the IRBs |
| Harms | 22 | Plan for collecting, assessing, reporting, and managing adverse events or any other unintended effects of the study will be communicated to the teams of supervisors and to the IRB. |
| Auditing | 23 | During the intervention period, the progress of the study will be evaluated each month with the intervention providers and the principal investigators. However, there will not be specific way of procedures for auditing the trial conduct. |

| Ethics and dissemination | | |
| --- | --- | --- |
| Research ethics approval | 24 | The study was approved by the University of Wollongong Human Research Ethics Committee with approval number 2022/212 on the 12^th^ of September 2022 and Bahir Dar University with approval number 563/2022 on the 24^th^ of October 2022. |
| Protocol amendments | 25 | Modifications to the protocol has not been planned. In case, if there is a plan to modify notification to relevant parties including the IRBs, the trial registers, and journals will be made. |
| Consent or assent | 26a | Informed consent will be obtained after full explanation of the objective and procedures of the study.  Who will obtain informed consent or assent from potential trial participants or authorised surrogates, and how (see Item 32) |
|  | 26b | Additional consent provisions for collection and use of participant data and biological specimens in ancillary studies, if applicable |
| Confidentiality | 27 | How personal information about potential and enrolled participants will be collected, shared, and maintained in order to protect confidentiality before, during, and after the trial |
| Declaration of interests | 28 | Financial and other competing interests for principal investigators for the overall trial and each study site |
| Access to data | 29 | Statement of who will have access to the final trial dataset, and disclosure of contractual agreements that limit such access for investigators |
| Ancillary and post-trial care | 30 | Provisions, if any, for ancillary and post-trial care, and for compensation to those who suffer harm from trial participation |
| Dissemination policy | 31a | Plans for investigators and sponsor to communicate trial results to participants, healthcare professionals, the public, and other relevant groups (eg, via publication, reporting in results databases, or other data sharing arrangements), including any publication restrictions |
|  | 31b | Authorship eligibility guidelines and any intended use of professional writers |
|  | 31c | Plans, if any, for granting public access to the full protocol, participant-level dataset, and statistical code |

| Appendices |  |  |
| --- | --- | --- |
| Informed consent materials | 32 | Model consent form and other related documentation given to participants and authorised surrogates  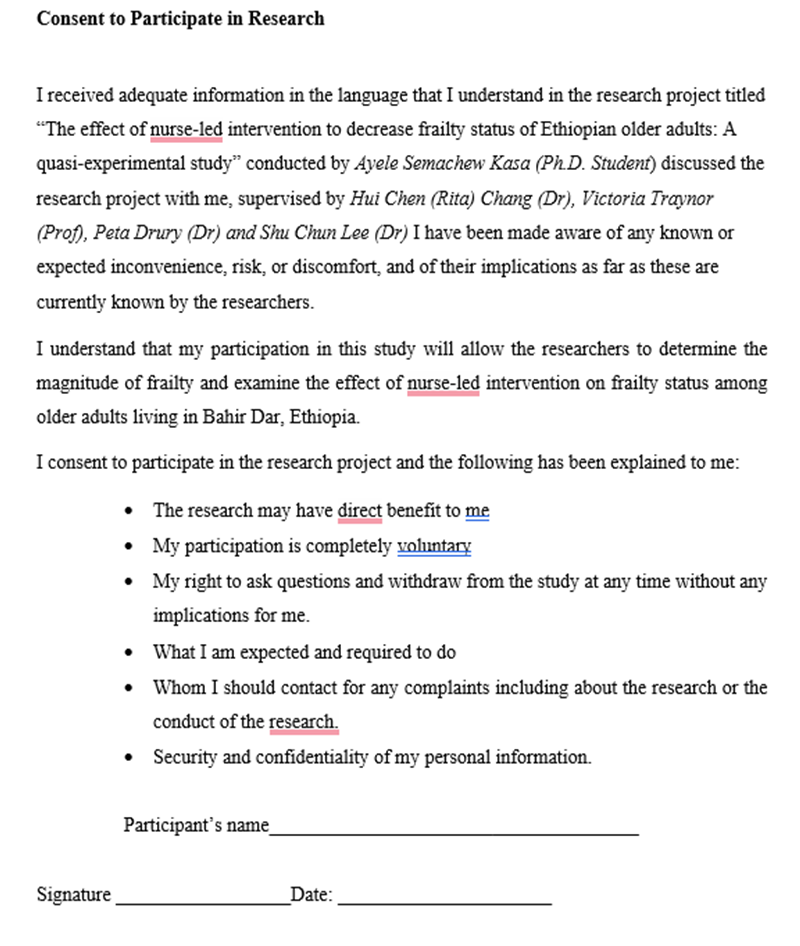 |
| Biological specimens | 33 | Confidentiality of the personal identity of the participants will be assured by using anonymous codes and securely stored data. Secure storage of data will be ensured by delivering data directly from data collectors to researchers, securely storing the completed questionnaire in a locked cabinet and restricting access to the PhD candidate only. |

*It is strongly recommended that this checklist be read in conjunction with the SPIRIT 2013 Explanation & Elaboration for important clarification on the items. Amendments to the protocol should be tracked and dated. The SPIRIT checklist is copyrighted by the SPIRIT Group under the Creative Commons “[Attribution-NonCommercial-NoDerivs 3.0 Unported](http://www.creativecommons.org/licenses/by-nc-nd/3.0/)” license.

Birhanie, Gebremeskel, Haimanot Melese, Gebrerufael Solomon, Berihu Fissha, and Molla Teferi. 2021. “Fear of Falling and Associated Factors among Older People Living in Bahir Dar City, Amhara, Ethiopia- a Cross-Sectional Study.” *BMC Geriatrics* 21(1):586. doi: 10.1186/s12877-021-02534-x.

Faul F, Erdfelder E, Buchner A, Lang AG. 2009. “Statistical Power Analyses Using G*power 3.1: Tests for Correlation and Regression Analyses.” *Behav Res Methods.* 41(4):1149–60.

Federal Negarit Gazeta of the Federal Democratic Republic Of Ethiopia. 2011. *A Proclamation to Provide For Public Servants’ Pension: Proclamation No. 714/2011*.

Ha, Jiyeon, and Yeon Hwan Park. 2020. “Effects of a Person-Centered Nursing Intervention for Frailty among Prefrail Community-Dwelling Older Adults.” *International Journal of Environmental Research and Public Health* 17(18):1–19. doi: 10.3390/ijerph17186660.

Haider, Sandra, Thomas E. Dorner, Eva Luger, Ali Kapan, Sylvia Titze, Christian Lackinger, and Karin E. Schindler. 2017. “Impact of a Home-Based Physical and Nutritional Intervention Program Conducted by Lay-Volunteers on Handgrip Strength in Prefrail and Frail Older Adults: A Randomized Control Trial.” *PLoS ONE* 12(1):1–15. doi: 10.1371/journal.pone.0169613.

HelpAge International. 2013. *Vulnerability of Older People in Ethiopia: The Case of Oromia, Amhara and SNNP Regional States*.

Des Jarlais et. 2004. “Standards for Reporting Non-Randomized Evaluations of Behavioral and Public Health Interventions: The TREND Statement.” *American Journal of Public Health* 94(3):361–66. doi: 10.1111/j.1360-0443.2004.00785.x.

Jemal, Kemal, Dejene Hailu, Bikila Tesfa, Tasfaye Lama, Tadele Kinati, and Endeshaw Mengistu. 2021. “Geriatric Depression and Quality of Life in North Shoa Zone, Oromia Region: A Community Cross-Sectional Study.” *Annals of General Psychiatry* 20(1):1–10. doi: 10.1186/s12991-021-00357-z.

Lee, Iris Fung Kam, Felix Ngok Yau, Sally Suk Ha Yim, and Diana Tze Fan Lee. 2018. “Evaluating the Impact of a Home-Based Rehabilitation Service on Older People and Their Caregivers: A Matched-Control Quasi-Experimental Study.” *Clinical Interventions in Aging* 13:1727–37. doi: 10.2147/CIA.S172871.

Marcus-Varwijk, Anne Esther, Lilian L. Peters, Tommy L. S. Visscher, Carolien H. M. Smits, Adelita V. Ranchor, and Joris P. J. Slaets. 2020. “Impact of a Nurse-Led Health Promotion Intervention in an Aging Population: Results From a Quasi-Experimental Study on the ‘Community Health Consultation Offices for Seniors.’” *Journal of Aging and Health* 32(1):83–94. doi: 10.1177/0898264318804946.

Markle-Reid, Maureen, Gina Browne, and Amiram Gafni. 2013. “Nurse-Led Health Promotion Interventions Improve Quality of Life in Frail Older Home Care Clients: Lessons Learned from Three Randomized Trials in Ontario, Canada.” *Journal of Evaluation in Clinical Practice* 19(1):118–31. doi: 10.1111/j.1365-2753.2011.01782.x.

Markle-Reid, Maureen, Robin Weir, Gina Browne, Jacqueline Roberts, Amiram Gafni, and Sandra Henderson. 2006. “Health Promotion for Frail Older Home Care Clients.” *Journal of Advanced Nursing* 54(3):381–95. doi: 10.1111/j.1365-2648.2006.03817.x.

Song, Mi Sook, and Sunjoo Boo. 2022. “Effects of a Nurse-Led Multicomponent Intervention for Frail Older Adults Living Alone in a Community: A Quasi-Experimental Study.” *BMC Nursing* 21(1):1–9. doi: 10.1186/s12912-021-00801-1.
