# Supplementary material for: Measuring the effects of a nurse-led intervention on frailty status of older people living in the community in Ethiopia: A protocol for a quasi-experimental study
Source: PLoS One. 2024 Jan 19;19(1):e0296166. doi: 10.1371/journal.pone.0296166 (PMC10798498; doi:10.1371/journal.pone.0296166)
Supplement: S2 File — (DOCX) [file pone.0296166.s004.docx]

**The Effects of a Nurse-led Intervention on Frailty Status of Older People Living in the Community in Ethiopia: A quasi-experimental study**

Research Proposal

**By Ayele Semachew Kasa**

**Supervisors:**

Professor Victoria Traynor

Dr. Peta Drury

Dr. Hui-Chen (Rita) Chang

Dr. Shu-Chun Lee

**Table of content**

[List of Abbreviations iii](#_Toc153265105)

[Achievements To Date iv](#_Toc153265106)

[Abstract v](#_Toc153265107)

[Introduction 1](#_Toc153265108)

[Background 2](#_Toc153265109)

[Research Aims and Objectives 5](#_Toc153265110)

[Literature Review 5](#_Toc153265111)

[Methodological approach 12](#_Toc153265112)

[Study setting, design, and sample 12](#_Toc153265113)

[Recruitment strategies 13](#_Toc153265114)

[Data Management 20](#_Toc153265115)

[Data Analyses 20](#_Toc153265116)

[Ethics 20](#_Toc153265117)

[Research Merit and Integrity 21](#_Toc153265118)

[Risk and Benefit of the Study 22](#_Toc153265119)

[Project Planning 22](#_Toc153265120)

[Dissemination strategy 22](#_Toc153265121)

[Timeline 23](#_Toc153265122)

[Resources 24](#_Toc153265123)

[Strengths of the study 25](#_Toc153265124)

[Limitations of the study 25](#_Toc153265125)

[Implications for practice 25](#_Toc153265126)

[Conclusion 26](#_Toc153265127)

[Acknowledgments 26](#_Toc153265128)

[References 27](#_Toc153265129)

[Appendices 41](#_Toc153265130)

[Appendix A: Literature review 41](#_Toc153265131)

[Appendix A 1: Review one 41](#_Toc153265132)

[Appendix A2: Review two 38](#_Toc153265133)

[Appendix B: Information Sheets & Consent Forms 62](#_Toc153265134)

[Appendix C: Data Collection Tools 68](#_Toc153265135)

[Questionnaires: English version 68](#_Toc153265136)

[Questionnaire: Amharic version 78](#_Toc153265137)

**List of Figures**

[Figure 1: Stages of the study. 4](#_Toc104408984)

[Figure 2: Conceptual model of the study adapted from the integral conceptual model of frailty. 11](#_Toc104408985)

[Figure 3: The flow chart of educational intervention of the study. 13](#_Toc104408986)

[Figure 4: Construct of the Nurse-led Intervention (NLI) program. 16](#_Toc104408987)

# List of Abbreviations

ADL: Activity of Daily Living

CHWs: Community Health Workers

GDS-15: Geriatric Depression Scales-15

HICs: High-Income Countries

ICMF: Integral Conceptual Model of Frailty

LTFU: Lost to Follow-up

LICs: Low-Income Countries

LMICs: Lower Middle-income Countries

NLI: Nurse-led Intervention

SNAQ: Simplified Nutritional Appetite Questionnaire

SSA: Sub-Saharan Africa

TFI: Tilburg Frailty Indicator

QOL: Quality of life

US: United States

# Achievements To Date

This section highlights achievements to date including:

Articles in press

Accepted conference papers

**List of research achievements relevant to PhD study**

- *Kasa Ayele S., Lee Shu C., and Chang Hui-Chen (Rita).* The extent of frailty in older adults living in Africa: Systematic review and meta-analysis. *Nursing Open (Article in press)*
- *Kasa Ayele S., Lee Shu C., and Chang Hui-Chen (Rita).* Depression among older adults living in resource-limited settings: A systematic review and meta-analysis. *BMC Psychiatry (Article in press)*

**Accepted papers for conference presentations relevant to PhD study.**

- *Kasa Ayele S., Lee Shu C., and Chang Hui-Chen (Rita).* The extent of frailty in older adults living in Africa: A systematic review and meta-analysis. ***Oral presentation*** in The Sigma's 33^rd^ International Nursing Research Conference to be held in Edinburgh, Scotland from 3-5 August 2022.
- *Kasa Ayele S., Lee Shu C., and Chang Hui-Chen (Rita).* The magnitude of depression among older adults living in a resource-limited settings: A systematic review and meta-analysis from Ethiopian perspective. ***Oral Presentation*** at the 7^th^ International Nursing Research Conference of World Academy of Nursing Science (7th WANS) Taipei, Taiwan from October 18-19/2022.

**First Year Proposal**

**Study Title**

The effect of a nurse-led intervention to decrease frailty status of Ethiopian older persons: A quasi-experimental study

# Abstract

**Background:** The number of older people is growing rapidly in sub-Saharan Africa, more so than in the developed world and will continue to do so in the future. However, in the context of sub-Saharan Africa, little work has been done on the frailty status of the older population. Frailty leads to a progressive disability in physical, mental, and social health unless appropriate measures are implemented to reduce its progression. To the researchers’ knowledge, no prior studies have attempted to assess the level of frailty and associated health consequences let alone the effectiveness of a nurse-led intervention to reduce frailty.

**Aim:** The overall aim of this study is to design, implement and evaluate the effect of a nurse-led intervention on frailty and associated health consequences among older persons living in Bahir Dar, Ethiopia.

**Methods:** The study will be conducted using a quasi-experimental study design on older persons living in Bahir Dar City, Amara Region, Ethiopia. One sub city from Bahir Dar will be selected using lottery method. The required number of older adults will be taken from the household’s registration of the city’s administration health office and from a health post (the lowest healthcare administration in the Ethiopian health tier system) in the selected sub city. To determine the effect of a nurse-led intervention on frailty status, data will be collected before intervention (baseline), immediately after the intervention and at the twelfth week of post intervention using validated survey tools. Data will be analysed using IBM SPSS 26.0 (IBM Corp., Armonk, NY, USA).

**Conclusion:** Previous studies from developed nations have recognized the impact of frailty on older adults and have developed several frailty interventions with positive outcomes from community settings. The proposed study is aimed to evaluate the effect of a nurse-led intervention on frailty status among older adults living in low-income settings.

**Implication for practice:** This study is the first nurse-led intervention designed to decrease frailty among older adults in Ethiopia. The educational material developed by the researcher will be implemented in the community settings as an educational resource. The study will demonstrate options on how a nurse-led intervention for older adults with frailty can be integrated with the existing Ethiopian health extension package. Healthcare professionals, especially nurses working in the community through home visiting will become familiar with the concept of frailty and associated screening tools when they assess older adults.

**Key words:** Nurse-led, intervention, education, frailty, elderly, Ethiopia

# Introduction

Population ageing will be the most important demographic dynamic worldwide in the coming decades. Currently, nearly 63 percent of the population age 60 and over live in developing countries (1). By 2025, this number is expected to increase to 75%. By 2050, one in five people in low-income countries will be over the age of 60 (2). Of the 15 countries that currently have more than 10 million older people, seven are developing countries (3). In these countries, the healthcare is focused on halting different infectious diseases, children, maternal and other healthcare related problems (4). The older adult has different and additional health needs compared to the younger adult. However, the healthcare system in many developing countries including in Ethiopia is not geared to address the health needs of older people (5).

Aging is a natural process, and the provision of care to the elderly, regardless of their background is a marker of quality healthcare. Unfortunately, inadequate healthcare infrastructure, insufficient financial capacity, and lack of research hinder the progress of the quality of aged care in low-resource countries (6,7). In addition to preventive care, the psychosocial needs of older populations are often over looked in Africa (8). Consequently, promoting health and quality of life (QOL) in older persons becomes challenging (9,10).

In Africa following Nigeria, Ethiopia has the largest older population, with over 6 million people aged 60 and over (11). The number of people aged 60 and over in sub-Saharan Africa (SSA) will nearly double from approximately 34 million in 2005 to 67 million in 2030. The number of older people is growing faster in SSA than in the developed countries and will continue to do so in the future (1). However, in the context of SSA, little work has been done to examine the health, frailty and well-being of the older population (1,8,12). To the researchers’ knowledge, no previous research has attempted to develop and assess the effectiveness of a nurse-led intervention to reduce frailty and assess the associated health outcomes.

# Background

Frailty is a geriatric clinical syndrome with numerous adverse outcomes (13). It is not caused by a single factor but is commonly influenced by multiple factors that can be interrelated or independent of each other (14,15). Progressive disability in physical, mental, and social health are manifestations of frailty. These signs greatly affect the older persons wellbeing and QOL (16). Frailty in the older adult population is influenced by a range of physical, behavioural, psychological, and social health factors (17–19). A systematic review and meta-analysis on the prevalence of frailty among community-dwelling older persons in Low-Income Countries (LICs) and Lower Middle-income Countries (LMICs) revealed that the frailty status in LICs and LMICs appeared to be higher than the weighted prevalence in High Income Countries (HICs) (20).

The recent recognition of the multidimensional features of frailty has highlighted the need for individualised multifactorial interventions, such as those focusing on the physical and psychosocial domains of health (21). However, previous studies focused on older residents in long-term care facilities focusing only the physical health of older people. Prior studies have rarely examined the effectiveness of interventions implemented to community dwelling older persons, who are not candidates for these services but are at risk of frailty (22,23).

The global increase in the number of older people and the accompanying increase of chronic conditions underline the necessity of health promotion and preventive intervention for the high-risk populations (24). Health education is one of the most important community-based prevention programs that motivate people to adopt healthy behaviors, develop positive attitudes, make decisions and incorporate the necessary skills to put their decision into practice (25). Clearly, educational programs and initiatives are vital for the prevention and management of frailty (26).

Interventions that detect frailty and promote health-related behaviours, seem promising to prevent falls, reduce morbidity and improve QOL in community-dwelling older people (24). In this regard, a nurse-led intervention can optimise health outcomes and reduce the need for acute hospital use among frail older persons living in the community (27). Nurses play an important role in delivering health promotion and preventive interventions to older people (28). Nurses are often in prime position to gather information about personal medical history, family background, as well as social, cultural, and economic factors that might be important in developing an intervention. Nurses are often able to apply their skills in health education to help older people to develop prevention and management strategies that are appropriate for their personal and family circumstances. They are also skilled at assessment, ongoing care, education, and family support (29).

Studies from developed countries have recognised the importance of frailty in older persons and have developed a number of frailty interventions with positive outcomes in community settings. These studies suggest that further research is needed (24) to evaluate the effectiveness of additional nurse-led interventions in other contexts and settings (30,31) with a relatively longer follow-up (32). A study conducted by Marker et al (33) also showed that proactively providing home-based care health promotion to frail older persons would improve QOL and reduce frailty status.

Nurses in community and primary care settings are in a key position to deliver healthcare solutions. Their skills and knowledge in providing education will make a substantial contribution to improving clinical and functional health outcomes (34). Nurses are on the frontline of health care provision in SSA and forming a bridge between health care institutions and the populations they serve (35). In Ethiopia, nurses are the backbone of the health care system (36) and the largest healthcare professionals (37) involved implementing various government funded healthcare interventions in the community.

Even though frailty has not been studied in Ethiopian context, it has been recognised by the World Health Organization and the United States (US) National Academy of Sciences as a major public health concern among older persons (38). Considering the importance of the health of older persons and the growing increase of this ageing population in Ethiopia, understanding frailty status and the effect of a nurse-led intervention on older persons with frailty can help in designing inclusive health promotion programs for older persons in Ethiopia.

However, research related to frailty is still at an incipient stage in many low income settings such as Africa (39) and to date no studies investigating frailty in Ethiopia have been conducted. There is a lack of research on frailty prevention interventions in older persons, and no studies have assessed the impact of a nurse-led intervention designed to decrease frailty among older persons in community settings. Therefore, this study aims to design, implement, and evaluate a nurse-led intervention to decrease frailty of older persons in the Ethiopia community. Overall, the study will comprise five stages (Figure 1)

Literature Review

1. Systematic reviews of frailty & depression
2. Identify a frailty framework
3. Identify a frailty measurement tool

Stage I

Stage II

Adapt a frailty tool for use within community populations in Ethiopia

Stage III

Develop a nurse-led intervention in collaboration with a community nurse

Implement and evaluate nurse-led intervention

Stage IV

Stage V

Sustainability & replicability of study i.e. practice and policy recommendations

Figure 1: Stages of the study.

# Research Aims and Objectives

1. To adapt the Tilburg Frailty Indicator (TFI) to Amharic version (TFI-AM).

- The specific objectives:
  - Translating TFI from English to Amharic (local language) and making cultural adaptions.
  - Testing the reliability and validity of the TFI-AM Ethiopian version.

1. Measure the effects of a nurse-led intervention on frail older people living in the community in Ethiopia.

- The specific objectives:
  - Develop a nurse-led intervention.
  - Implement the nurse-led intervention.
  - Measure the effects on frailty, nutritional status, depression, activities of daily living and quality of life.

**Research Questions**

The following research questions will be addressed to achieve the overall aims of this study:

- Does the nurse-led intervention decrease the frailty including the physical, social, and psychological domains among the older persons living in the community in Bahir Dar, Ethiopia?
- Does the nurse-led intervention improve the quality of life among the older persons living in the community in Bahir Dar, Ethiopia?

**Study Outcomes**

**Primary Outcome Measures**

- The effect of nurse-led intervention on the frailty status on older persons.

**Secondary Outcome Measures**

- Sociodemographic related correlates/predictors of frailty in older persons.
- Activity of daily living
- Nutritional status
- Depression
- Quality of life

# Literature Review

To determine the prevalence of frailty in older persons in African, one systematic review and meta-analysis has been completed. The findings indicated that a significant number of older persons have frailty. Overall, the review revealed that 38.64% of older persons from Africa had frailty. This systematic review and meta-analysis has been submitted to Nursing Open Journal for publication and is under review. In addition, a second systematic review and meta-analysis was done to assess the magnitude of one of the secondary outcomes. One of these secondary outcomes is depression. Studies indicated that the correlation of frailty and depression is substantial (40,41). Depression is common in later life, especially when people are frail, which can lead to reduced QOL (42). Since no previous studies determined the level of frailty in Ethiopia this review didn’t address the level of depression among frail older persons. As a result, the review sought to determine the level of depression among older persons living in Ethiopia. The result showed that a significant number of older persons are living with depression. Hence, from this work it can be hypothesised that if the level of frailty among older persons in Ethiopia is known and depression is assessed, the level of depression among older persons might be worsened than the resent finding. The abstract of the first and the second systematic review and meta-analysis papers are presented below, and the full-text manuscript of these papers are provided in Appendix A.

**Abstract #1**

**Title of the review:** The extent of frailty in older adults living in Africa: Systematic review and meta-analysis

**Background:** Although Africa is the second largest continent in terms of population, only a few studies, which have not been meticulously conducted, have been performed about frailty among older adults.

**Objective:** To determine the pooled prevalence of frailty in the African continent through a meta-analysis and to identify factors associated with frailty.

**Methods:** Eleven studies were selected based on predefined inclusion and exclusion criteria. Data were extracted using a standardised data extraction checklist, and analysis was conducted using STATA 14 statistical software. The Cochran Q test and inverse variance weighting were used to assess heterogeneity. Because considerable heterogeneity was noted, a random effects meta-analysis model was used to estimate the pooled prevalence of frailty.

**Results:** A forest plot of the 11 included studies revealed that the overall pooled prevalence of frailty among older adults in Africa was 38.64% (95% confidence interval [CI]: 26.11, 51.17). Subgroup analysis indicated the highest prevalence of frailty in West Africa, with a prevalence of 57.23% (95% CI: 45.47, 68.99), and studies in institutional settings revealed the highest prevalence of frailty at 53.04% (95% CI: 38.28, 67.80). In this study, the presence of a comorbidity (AOR = 1.6, 95% CI: 1.77, 2.18) was associated with a status of frailty among older adults.

**Conclusion:** This systematic review and meta-analysis revealed that frailty is highly prevalent among older adults in Africa. Therefore, inclusive and comprehensive research that addresses the health of older adults in Africa must be conducted.

**Keywords:** Frailty, elderly, aged, older adults, Africa

The full paper found in Appendix A1.

**Abstract #2**

**Title of the review:** Depression among older adults living in resource-limited settings: A systematic review and meta-analysis

**Objective:** This systematic review and meta-analysis aims to estimate the overall prevalence of depression and identify related factors among older people in Ethiopia.

**Methods:** Multiple databases with no date limit were searched. The Preferred Reporting Items for Systematic Reviews and Meta-Analyses (PRISMA) was also utilized. The quality of the included studies was assessed using the Joanna Briggs Institute quality appraisal tool. The presence of publication bias was evaluated by performing Egger’s test and a visual inspection of the symmetry in funnel plots.

**Results:** The overall prevalence of depression among older adults in Ethiopia was 41.85 (95% CI = 33.52, 50.18). Relative to other regions of Ethiopia, the Oromia region had a higher prevalence of depression at 48.07% (95% confidence interval [CI] = 35.62, 60.51). Female older adults (adjusted odds ratio [AOR] = 1.76; 95% CI = 1.17, 2.63), older adults with no formal education (AOR = 1.82; 95% CI = 1.03, 3.19), older adults with chronic diseases (AOR = 2.46; 95% CI = 1.00, 6.06), and with low or no social support (AOR = 2.01; 95% CI = 1.06, 3.83) had a significantly greater prevalence of depression.

**Conclusion**: Our systematic review and meta-analysis revealed that almost two out of five older adults had depression. Female sex, absence of formal education, having chronic diseases, and low or no social support were the independent predictors of depression among older adults in Ethiopia. Our systematic review and meta-analysis highlight that depression among older adults in Ethiopia is a public health problem.

**Keywords**: Depression, prevalence, predictors, risk factors, older adults, elderly, Ethiopia

The full paper found in Appendix A2.

**Theoretical Perspective**

**Conceptual and Theoretical Models**

Every scientific model is associated with a philosophical framework, which presents a distinct and formalised description of the nature and development of scientific knowledge (43–45). Models are descriptions or analogies used to help us visualize things that are not typically observed directly (46). Before the 1980s, the terms “frail” or “frailty” were rarely used to refer to older persons (46). Frailty is a nonspecific state of increasing risk, which reflects physiological changes in multiple systems. It is highly age-associated (47). Various operational schemes have been employed to find a definition of frailty (48). It is a health problem characterized by increased vulnerability and decreased reserve capacity (49,50). It can occur as a result of a range of diseases and medical conditions (49). It has traditionally been a bio-medically dominated concept that is defined as a clinical syndrome or vulnerability in different physical states (13,51). Such a narrow conceptualisation of frailty emphasises only its biomedical indicators and ignores the multidimensional features of frailty (52). Although frailty is increasingly recognised as a critical health problem in older persons (53,54) a universally accepted frailty model to determine the consequence of frailty is scarce (55). Models of frailty vary widely depending on the population studied, study design, and methods used. To date, despite these variations, frailty has generally been conceptualized using two different models, namely, Fried’s Frailty Model and Integral Conceptual Model of Frailty (ICMF).

**Fried’s Frailty Model**

Most conceptual and operational definitions of frailty place heavy emphasis on physical loss in older people. The Fried’s frailty conceptual model based chiefly on the medical sciences (43). This model (56) is the known model for observing frailty using physical phenotypes. In this model, the definition of frailty focuses only on the physical domain of human functioning (17,57,58) whereby the psychological and the social domains are neglected.

Various authors and researchers argue that the definition of frailty according to this model is too narrow, only focusing on the physical state (43,59). This leads to concern that if the definition and accompanying model of frailty relays solely on the physical state of older persons, attention for the individual as a whole is threatened (60). This could potentially lead to disintegration of care, thereby reducing the quality of care provided to frail older persons (61). A literature search revealed that a growing number of researchers have criticised the one-sided focus on physical frailty and convinced of the multifactorial nature of the concept of frailty (43,46,62,63).

**Integral Conceptual Model of Frailty (ICMF)**

Physical, mental, cognitive, and social functioning changes with age. Accumulation of problems in one or more of these domains of functioning is characteristic for a frail individual (64). As persons with frailty may deal with multiple health problems, it is particularly important to provide a person-centered care approach that is tailored to the individual and their environment context (65). To provide comprehensive and holistic aged friendly care, frailty should be seen from different dimensions. From the ICMF (66) perspective, frailty seen as a dynamic state affecting an individual who experiences losses in one or more domains of human functioning (physical, psychological, social), which is caused by the influence of a range of variables.

A literature review that included different studies in older persons found that the prevalence of physical frailty varied and was higher when psychosocial frailty was included (60). In another review, the most used components of frailty were identified within the physical, psychological, and social domains. Across all three domains, the most common identified factors of frailty were mobility, nutrition, and cognitive function (62). A multidimensional definition of frailty requires looking beyond adverse outcomes that are mainly related to the physical domain, and it is important to include QOL as an adverse outcome (17). Quality of life is defined as an individual’s perception of their position in life in the context of the culture and value system in which they live, and in relation to their goals, expectations, standards and concerns (67). Hence, the ICMF which includes the physical, psychological, and social domains of frailty chosen as the most appropriate model to guide this study (64,68) (Figure 2).

**Environmental:**

- Living arrangement
- Health facility visit

**Physical health status**

- Presence of comorbidities
- Appetite
- Nutritional status
- Fall history

**Sociodemographic factors**

**Social support**

**Personal lifestyle and activity:**

- Physical functioning (Activity of daily living)
- Lifestyle

**Psychological health status**

- Depression

**Adverse outcomes:**

- Poor **QOL**


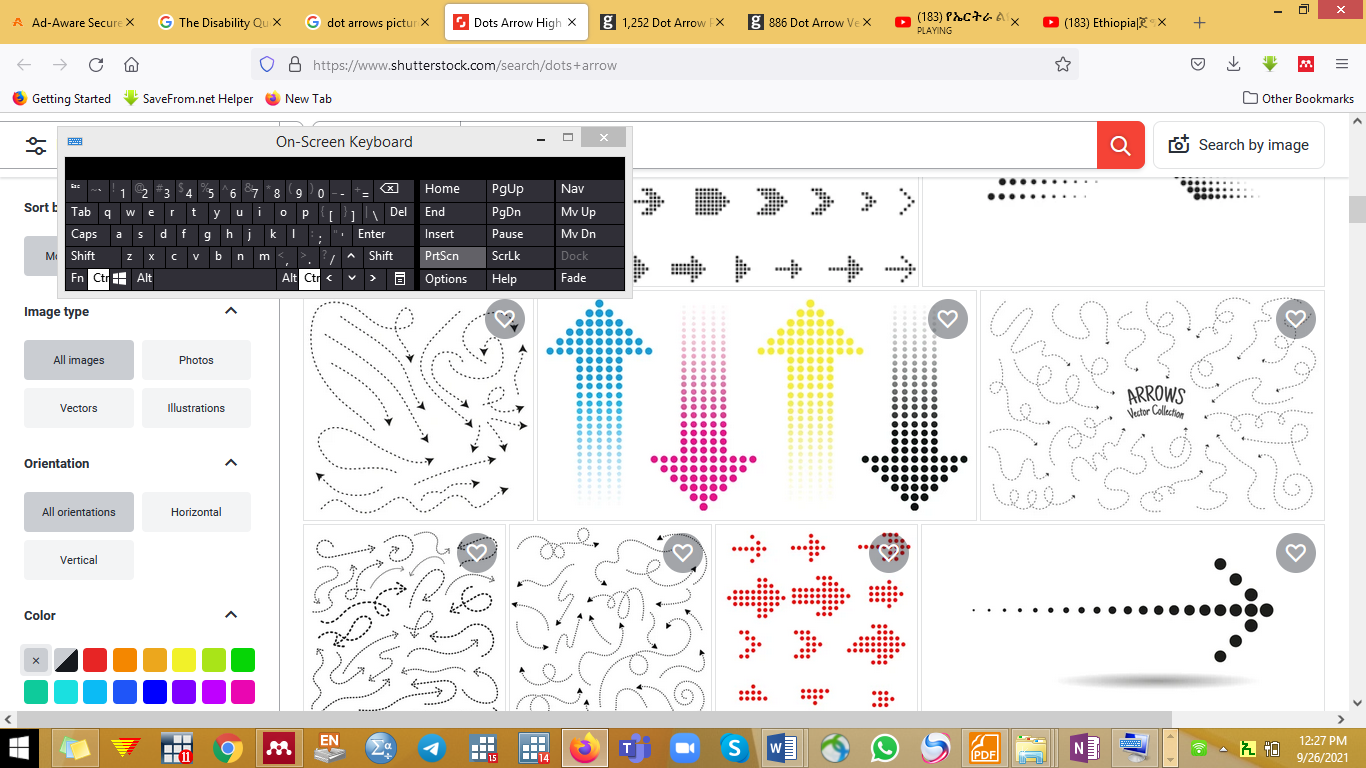

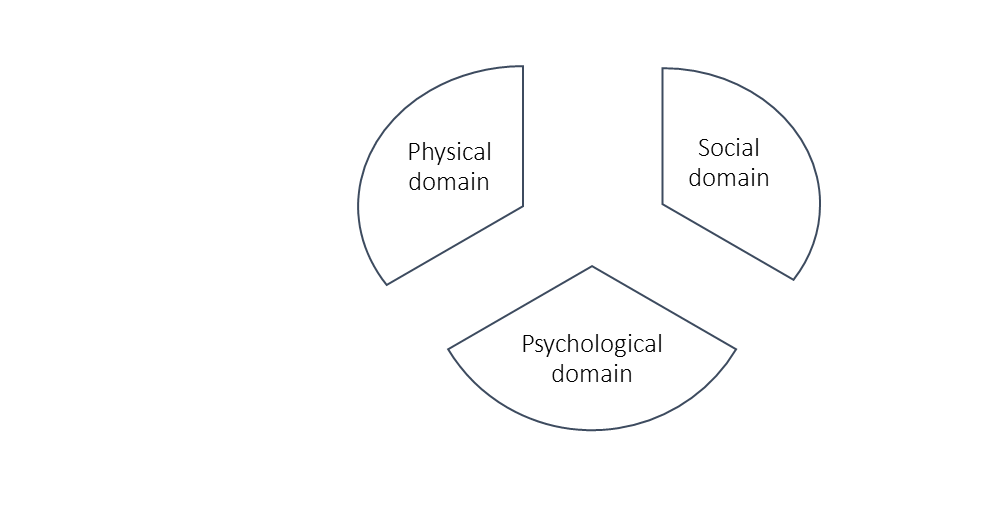


**Frailty**

Figure 2: Conceptual model of the study adapted from the integral conceptual model of frailty.

(17,43,52,64,69).

## Methodological approach

A quasi-experimental study design will be adopted to examine the effect of a nurse-led intervention on frailty among older persons living in Bahir Dar City, Amara Region, Ethiopia. This study will adhere to the Transparent Reporting of Evaluations with Nonrandomized Designs (TREND) guidelines (70).

## Study setting, design, and sample

**Study setting**

The study will be conducted in Bahir Dar, Ethiopia. Bahir Dar is the capital city for the regional state of Amhara situated on the outskirts of the famous Lake Tana, 552km north of Addis Ababa, the capital city of Ethiopia. The city has six administrative sub-cities with over 400,000 population. Based on a survey conducted by Bahir Dar City Labour and Social Affairs Administration Office in 2018 revealed that there were over 3,300 older persons in Bahir Dar City administration (71).

In Bahir Dar City there are three public and four private hospitals, 10 health centers and 15 health posts. In the Ethiopian health tier system health posts, health centres and primary hospitals are regarded under primary level health care whereas general hospitals and specialized hospitals regarded as secondary and tertiary level healthcare respectively (72). Bahir Dar city administration health office oversees all primary level healthcare facilities in the city administration (73).

**Sample size**

To determine the sample size, a two-tailed test with a significance level (α) of 0.05, power (1-β) of 0.8, and effect size (d) of 1.12 based on a previous study on frailty of nurse-led education intervention (32) were assumed. However, the sample size become very small and as a result the study sample size is calculated using a priori computation of sample size using G* Power version 3.1.9.4 (74) with assumption of a two-tailed test with an alpha value of 0.05, effect size (f) of 0.5, and a power of 0.95 revealed that 55 participants are required. By considering a 10 to 20% (31,32) withdrawal rate during the intervention, at least 68 study participants will be required.

### Recruitment strategies

One sub-city from Bahir Dar city will be selected using the lottery method. The list of older persons in the selected sub-city will be selected from the household’s registration of the city’s administration health office or from the health post. Initially, study participants will be contacted by the Community Health Workers (CHWs) through home-to-home visit for explaining the aim of the study, getting consent, screening frailty status, and their interest to participate in the intervention/study. CHWs are registered nurses working in the same area where study participants residing and work at the health post and home visiting.

After confirming study participant eligibility and willingness to join the study, a baseline questionnaire will be administered prior to the start of the nurse-led intervention. At the end, study participants who received all the nurse-led intervention sessions will be included in the final analysis to determine the effectiveness of the nurse-led intervention in reducing frailty among older persons in Ethiopia (Figure 3).

Assessed for eligibility/screening (n=?) Required sample (n=68)

Baseline data (n=TBD)

Study participants who provided consent for participation (n=TBD)

Follow-up status (n=TBD), with reason. Eg. LTFU (Transfer out, accident, death etc.)

Study participants for analysis (n=TBD)

Eligibility checks

Contacted at baseline for consent

Baseline data collection

Follow-up

End line

Figure 3: The flow chart of educational intervention of the study.

TBD: To be determined

**Eligibility**

**Inclusion and exclusion criteria**

The definition of old age depends on various countries’ settings to determine the old age cutoff point (75). In Ethiopia, the cut points of old age started from 60 years (76,77). Hence, older persons aged 60 years or above, whose frailty score ≥ 5 as measured by the Tilburg Frailty Indicator (TFI) and residing in Bahir Dar, Ethiopia will be included in the study. Participants will be excluded if they are unable to communicate, have cognitive impairment, are bed redden, not living at home, have been hospitalised with a known psychiatric problem within the past six months, and will remain in the selected area during the study period.

**Nurse-led education material and execution of the program**

A nurse-led education intervention handbook contextually relevant to frailty management for the older people will be developed. The content of the training handbook will be based on the multidimensional concept of frailty (30,62,64) and will be customized to the local settings. The training handbook will be accompanied by illustrative pictures. For appropriateness in context, the training handbook will be reviewed by community nurses with experience in community health care services and older people. The training handbook will be translated into the local language, Amharic, and reviewed by a bilingual expert from Bahir Dar University, Ethiopia. A booklet on frailty management education will be disseminated to the study participants during the first session of the nurse-led education. For their convenience and to facilitate the participation of the older people in the study, the educational sessions will be conducted at participants’ own home.

**Intervention**

Based on the ICMF framework, physical, psychological, and social domains are key components to ensure the health of frail older persons. The intervention comprises six components (modules) offered each month for six consecutive months each session lasting approximately 30 to 40 minutes. All the six sessions will be delivered through a face-to-face approach. Each session will deliver one module with a total of six modules including the topic of ageing and age-related changes, healthy nutrition, physical activity, mental health issues, social interaction and support and at the last of the session (at the 6^th^ month) discussion and reflection about the overall sessions will be done. In the intervention period, there will be also a fort nightly 5 to 10-minute follow-up phone calls with study participants to get feedback on their training or to provide counseling (Figure 4).

Study participants will be offered a home-based intervention by a PhD candidate in nursing in collaboration with CHWs. CHWs working in the community, where older persons live, will assist the researcher in recruiting participants and facilitating the educational sessions. CHWs are registered nurse who are employees of the local government and work closely with the local community home to home and at health post (78). CHWs know the culture, life style and norms of the community and they provide culturally appropriate health education and information, help people get the care they need, counsel and guide on health behaviours, and for the health needs of individuals and communities (79).

Before the intervention is started, two CHWs will undergo three days of training on the study aim, training handbook content and procedure, and how study participants will be approached ethically. During each educational session, the PhD candidate will lead the intervention and CHW will facilitate training on the session topic of the day, conclude the educational session, and provide participants with take-home message. At the next session, after reflecting on how the participants undertook their take-home message the new educational session of the day will begin. If a participant asks questions beyond the scope of the intervention material, depending on the nature of the question they will be advised to meet with a medical expert at a nearby healthcare facility. To reduce lost to follow-up (LTFU), and increase their adherence in implementing training content, participants will be encouraged and reminded by phone to attend upcoming sessions. The study construct of the Nurse-led Intervention (NLI) is indicated below (Figure 4).

**Data Collection Process**

The data will be collected through a face-to-face administered structured survey questionnaire, and anthropometrical physical measurements. To reduce assessor bias, CHWs will not be involved in the data collection process. Hence, two professional nurses from Bahir Dar city will be recruited for data collection. The data collectors will not be involved in providing the intervention. They will be trained for two days about measurement tools, and how the study participants will be approached ethically. To determine the effect of a nurse-led intervention on frailty status among older persons, data will be collected at baseline (before intervention) (T0), immediately after the intervention (T1) and at twelfth week of the intervention (T2) (Figure 4) (32,80,81).


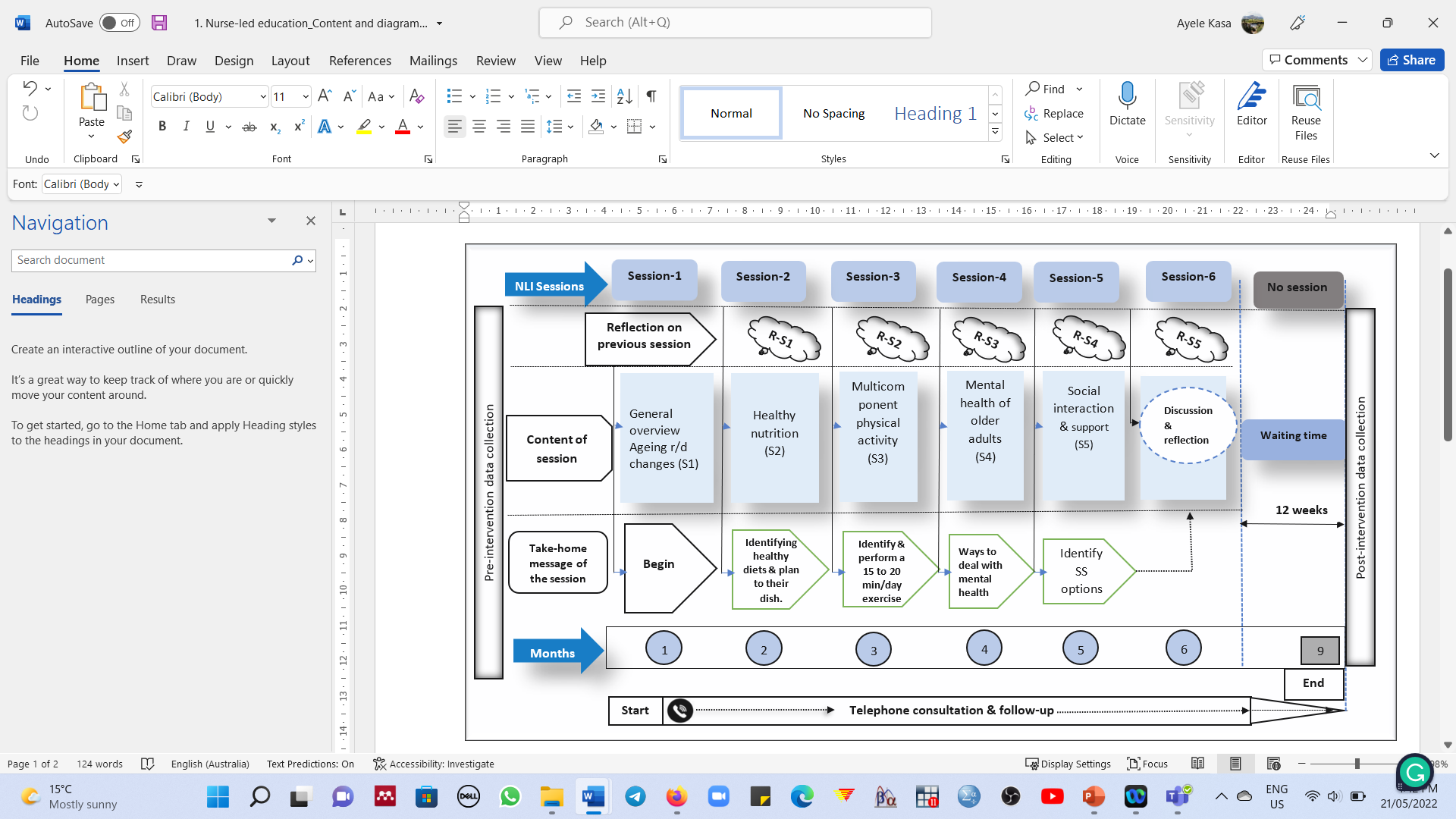


Figure 4: Construct of the Nurse-led Intervention (NLI) program.

R-S1: Reflection on session 1

**Data collection tool and Technique**

Different literatures stated that frailty in older adult is influenced by physical, psychological, and social health factors (17–19). Studies also showed that interventions have beneficial effects on physical functioning, nutritional status, and depression among community-dwelling frail older people (82–84). Therefore, to answer the primary and secondary outcomes of this study, different data collection instruments will be utilised. This study will employ both structured questionnaire, and anthropometrical physical measurement as data collection methods. The structured questionnaire comprises nine parts.

**Part I:** Participant’s socio-demographic information.

**Part II:** Participant health related information including medical history, health care service utilisation, living arrangement, lifestyles, or personal related behaviours such as Khat chewing, smoking/drinking habit.

**Part III: Primary outcome measurement**

The primary outcome will be measured using the Tilburg Frailty Indicator (TFI). TFI is a well-known and user-friendly instrument for assessing frailty in older people (85). It is valid and reliable instrument for assessing frailty in community-dwelling older persons (85–87). The TFI comprises 15 self-reported questions, divided into three distinct domains. The physical, the psychological and the social domains are the three distinct domains that constitutes the TFI. The physical domain consists of eight questions related to different physical health of the client. The psychological domain contains four items related to psychological health of the clients. The last domain, social domain has three questions related to social relation. Eleven of the TFI items have two response categories as “yes” or “no” options, while three items from the psychological domain and one item from the social domain have three response categories as “yes”, “no,” or “sometimes”. “Yes” or “sometimes” responses are scored 1 point each, while “no” responses are scored 0. The instrument’s overall score ranges from 0 to 15: the higher the score, the higher one’s frailty. Frailty is diagnosed when the total TFI score is ≥5 (87,88).

**Secondary outcome measurements**

**Part IV:** To assess nutritional status of the study participants Mini Nutritional Assessment (MNA) tool will be utilised (89,90). MNA is widely used and was developed specifically for use in older adult (91,92). The tool appears to be the most appropriate nutrition screening tool for use in community-dwelling older persons (93). Using the MNA score, participants will be classified as malnourished (<17), at risk of malnutrition (17–23.5) or normal nutritional status (24–30) (90). Anthropometric measurements (height and weight) will be taken without heavy outdoor clothing. Height will be measured to the nearest millimeter using standard and caliber anthropometric rod. Weight will be measured on a pre-standardized body weighing scale in kilograms. In addition, calf circumference will be measured to the nearest millimeter using a tape meter. The study participant will sit on a chair and hold his/her bare foot down, holding the leg folded to 90 degrees. Using a tape measure the circumference of the calf at its widest point will be measured, laying the tape on the skin without tightening. The measurement will be taken at more than one point to ensure measurement is taken at the widest part.

**Part V:** The Simplified Nutritional Appetite Questionnaire (SNAQ) will be used to assess the recent appetite status of the older persons. The SNAQ is a brief, valid and reliable four-item survey tool with a maximum score of 20 points and a score of <14 points indicate significant risk of at least 5% weight loss within six months (94,95).

**Part VI:** This section comprises questions that measures mood and depression of the participants. The Geriatric depression rating scales-15 (GDS-15) (91,96,97) will be utilised to assess the depression status of older persons. The scale has been extensively tested and validated in low and middle-income countries and across cultures (98). Depression is considered using a cutoff point greater than or equal to five. The outcome will be coded depending on the cutoff point 0–4 as normal, 5–8 as mild depression, 9–11 as moderate depression, and 12–15 as severe depression (75,96).

**Part VII:** This section encompasses questions that measure participants’ social support. Participants’ social support will be assessed with the short version of Social Support Questionnaire (SSQ-6). The SSQ-6 is a six-item instrument used to determine the number of people involved in providing support and to measure the level of satisfaction with the support. The satisfaction items are to be rated on a 6-point Likert scale, with the possible total score of 6-36; a higher score indicating more satisfaction with the available social support. This tool was found to have strong internal consistency and showed satisfactory validity (99).

**Part VIII:** The Katz Index of Independence in Activities of Daily Living, commonly referred to as the Katz ADL will be utilised to measure activity of daily living and functional independence (physical functioning) of older persons. The Katz ADL is the most appropriate instrument to assess functional status of older persons as a measurement of the client’s ability to perform activities of daily living independently (100). It is consistently demonstrated as valid and reliable tool in evaluating functional status in the elderly population (101).

The Index ranks adequacy of performance in the six functions of bathing, dressing, toileting, transferring, continence, and feeding. Older persons are scored yes/no for independence in each of the six functions. A score of 6 indicates full function, 4 indicates moderate impairment, and 2 or less indicates severe functional impairment (102,103).

**Part IX:** Quality of life of the study participants will be measured using the World Health Organization’s Quality of Life Questionnaire (WHOQOL-BREF) (75,104). The WHOQOL-BREF is reliable and valid instrument that would applicable cross-culturally (105). WHOQOL-BREF is a self-report questionnaire or interviewer-administered which contains 26 questions categorized into four domains that are scored on a 5-point Likert scale. The four domains are physical health (7 items), psychological health (6 items), social relationships (3 items), and environment (8 items). In addition, there are 2 items that measure overall QOL and general health. The tool can provide data for both research and clinical purposes. Although it is a relatively brief instrument, its structure allows one to acquire specific information covering many aspects of life (106).

**Reliability/validity**

The TFI questionnaire will be culturally adapted as it was not utilized in Ethiopia before. According to the literature, the minimum sample size for tool adaptation and reliability testing the sample should be at least five times larger than the number of variables being analysed (88). Hence, the TFI adaptation and reliability testing will be conducted on 75 older persons living in Bahir Dar city community settings. This test will be conducted before the start of the actual study on older persons who will not be included in the main study.

### Data Management

All data will be collected as hard copy print outs and kept in a locked filing cabinet for a period of five years by the PhD candidate. Collected data will be entered into EpiData software manager and individual paper ID number coding will be given instead of personal identifiable information. Furthermore, a securely password protected USB drive will be used to store the data set and statistical analysis outputs.

## Data Analyses

Fully completed and cleaned questionnaires will be entered in EpiData Manager software and exported to the IBM SPSS 26.0 (IBM Corp., Armonk, NY, USA) for analyses. To understand the correlates of frailty among older persons, Pearson correlation analysis will be utilised. Numerical and categorical data will be summarized as mean (±*SD*) and frequencies, respectively. Normality of the data will be checked. To measure the effect of a nurse-led intervention on the level of frailty among older persons Fisher's exact test, paired sample *t* test, and repeated measures ANOVA will be used. If the data is not normal, their nonparametric equivalents (Wilcoxon and Friedman tests) will be applied. Statistical significances will be set at *p*-values < 0.5.

## Ethics

Ethical approval to conduct the study will be obtained from both the University of Wollongong and Bahir Dar University Ethical review boards. In addition, permission will be requested from the study site administrations. The study participants will be contacted by the Community Health Workers (CHWs) who most of the study participants know CHWs. Then, discussion with the study participants about the nature of the study, frequency of nurse-led intervention, duration of each session, telephone follow-up, and data collection plans will be assured. In addition, they will be informed about who will collect the data and provide the intervention.

Informed consent will be obtained after full explanation of the objective and procedures of the study. For the participants, no risks are anticipated from being involved in participating to the study. The voluntary nature of participation will be emphasised. Participants will be given the opportunities to ask questions, and full responses will be given to the questions until no further queries raised. Participants will be also assured their right to withdraw from the study at any time.

Confidentiality of the personal identity of the participants will be assured by using anonymous codes and securely stored data. Secure storage of data will be ensured by delivering data directly from data collectors to researchers, securely storing the completed questionnaire in a locked cabinet and restricting access to the PhD candidate only.

### Research Merit and Integrity

The widespread issues such as scientific fraud, questionable research practices, and the reliability of scientific results have led to growing concerns about research integrity (107). Researchers must protect and uphold the integrity of the research, including managing bias and responding to any complaints effectively (108). Researchers who meet their research responsibilities are considered to have integrity, which is sometimes equated with ethical conduct (109).

To ensure the research merit and integrity, the responsible conduct of research process has been taken into consideration. At every stage of the research work, the necessary actions will be taken to comply with the rules of research merit and integrity. Research merit and integrity is considered in all the stages from research question framing, literature searching, proposal development, data collection, interpretation, writing up and communication phases.

This study aims to answer frailty status and the effects of nurse-led intervention on the frailty among older persons in a resource limited setting using a quasi-experimental study design. Although frailty among older persons is an issue in many countries, no study has been conducted in Ethiopia. The potential benefit of this study is to show how the problem is prevalent in older persons, and how nurse-led intervention is effective to decrease frailty among older persons in resource limited settings. All these findings will influence healthcare professionals, healthcare administrators and policy makers. Finally, the study will demonstrate how nurse-led intervention in managing older persons with frailty be integrated with existing health extension package in Ethiopia

**Consent**

Informed consent is the process of informing potential research participants about the key elements of a research study and what their participation will involve.  The informed consent process is one of the central components of the ethical conduct of research on human subjects (108).  The consent process usually includes providing a written consent document containing the required information (110).

In this study participants will be informed about the aims, frequency, duration, and type of intervention of the study. Study participants will also be informed that participation in the study is entirely voluntary. Reasonable expected benefits and foreseeable risk will also be informed. Study participants will be informed that if they have any question to ask, they will have the right to ask the data collectors or the researcher during anytime of the study. In addition, study participants will be informed they have the right to withdraw or refuse from the study at any time. Refusal to participate in the study will not have negative impact on them.

Depending on the literacy level of the study participants, both written and oral consents will be considered. Researchers prefer to have a written consent from study participants. However, written consent is not possible from illiterate participants and in certain cultural settings (111).

### Risk and Benefit of the Study

The risks of the research should be assessed for the potential harm, discomfort, inconvenience of people who involved in the research. Assessment of risk, including identify any risks, gauging their probability and severity, assessing the extent to which they can be minimised, determine whether they are justified by the potential benefits of the research, and determining how they can be managed (109). The current research doesn’t have a known risk or harms to the study participants. During the intervention, participants will be asked to explain if they feel any discomfort imposed by the study.

**Confidentiality**

Confidentiality in the context of human research is a statement that declares about how the study participants identifiable private information will be handled, managed, and disseminated (112). Confidentiality involves understanding between the researcher and participant that guarantees sensitive or private information will be handled with the utmost care. Ultimately, confidentiality is rooted in trust (113). The information collected from the study participants will be kept in a secured place and handled strictly confidential. Confidentiality will be insured in all the stages of the research, and it is designed to protect the privacy of the study participants during data collection, analyzing, and reporting data. Anonymity will be insured by collecting data without obtaining any personal identifying information.

# Project Planning

## Dissemination strategy

The finding of the study will be submitted to UOW, Bahir Dar University College of Medicine and Health Science, and Amhara National Regional Health Bureau. Oral presentation will be made in different national and international conferences. The findings from the research work will be published in age and ageing related scientific and peer reviewed journals.

The following journals will be considered to submit the manuscript produced from the research work for publication.

- Geriatrics and gerontology international
- Age and Ageing
- Psychogeriatrics
- Annals of geriatric medicine and research
- Geriatric nursing
- BMC geriatrics

**Impact**

- Decreased frailty status of older persons after implementing the intervention.
- Developed the educational intervention handbook to deliver the intervention and translate into further practice.
- Handbook and booklet on frailty accessed by the healthcare professionals across city.
- Healthcare professionals, researchers and policy maker will pay more attention on frailty issues.
- Create opportunity for healthcare professionals working in the community to assess frailty.
- Template for trialing the intervention for frailty prevention and management in other clinical settings or other low-income countries.

## Timeline

The study timeline will encompass different activities. In the first year of the study, the following activities will be done: attending different research training webinar and seminars, completing literature review, and writing the research proposal. In the second year of the study, ethical clearance application, communication with relevant governmental bodies, providing training for data collectors and CHWs, recruiting the study participants and data collection will be completed. In the third year of the study, data entry and analysing and writing up the results will take place and in the final year of the study writing thesis and submitting manuscripts to peer-review journals will be achieved.

Table 1: Timeline of the study.

## Resources

To carry out the study, different resources will be purchased and utilized.

- Financial resource
- Anthropometric measuring devices (Hight, weight and circumference measuring tapes)
- TheraBand
- Stationaries
- Hard disk
- Bags

**Discussion**

## Strengths of the study

This study is a first in its kind in resource limited settings. No prior studies attempted to examine the effectiveness of nurse-led intervention in lowering the frailty status of older persons in African. Quasi-experimental research designs, like experimental designs, test causal hypotheses and these designs are also used to test the effectiveness of a program. Both the primary and secondary outcome variables will be collected before and after implementing the intervention.

In resource limited settings, there is limited focus on the health of older persons. Researchers also didn’t address the health need and healthcare of older persons in their research in resource limited settings. This study will bridge the existing gap on the outlook of healthcare managers, researchers and healthcare providers towards the healthcare and need of the older persons.

Having strong connection with local health office administrators will help the researcher to conduct the research in the study area.

## Limitations of the study

Home based data collection and intervention will be time consuming for the research. As a type of experimental study with relatively a longer follow-up, lost to follow-up may be an issue.

## Implications for practice

The research will show ways on how to integrate the care of older persons with the existing health extension work in Ethiopia.

Healthcare professionals, especially nurses who work in the community will be familiar with how to use the screening tool for frailty when they assess older persons during their home visits. They will also use the interventional handbook developed by the researchers as a reference material in providing similar tasks in the community.

The finding will open the door for researchers and concerned government officials to look at the multidimensional healthcare need of older persons living in resource-limited settings.

# Conclusion

Frailty in older population is influenced by a wide range of physical, behavioral, and psychosocial health factors. The recent recognition of the multidimensional nature of frailty has highlighted the need for individualized multifactorial interventions. Studies from developed countries have recognized the importance of frailty in older persons and have developed a number of frailty interventions with positive outcomes from community settings. The proposed study is aimed to examine the effect of nurse-led intervention on frailty status among older persons living in low-income settings.

# Acknowledgments

I would like to acknowledge the University of Wollongong (UOW) for granted the University Postgraduate Award (UPA) Scholarship and International Postgraduate Tuition Award (IPTA) to attend my study and develop this study proposal. This achievement will not happen if this support was not given by the UOW.

# References

1. Velkoff VA KP. Aging in Sub-Saharan Africa: The Changing Demography of the Region. National Academies Press (US). 2018. 55–92 p.

2. Jacksonvilli University. Nurses Caring for Geriatric Patients. 2008. p. 3.

3. United Nations Population Fund. Ageing in the Twenty-First Century : A Celebration and A Challenge. 2012.

4. Commissioner UH rights O of the high. United Nations Principles for Older Persons. 1991.

5. Kelly G, Mrengqwa L, Geffen L. “They don’t care about us”: older people’s experiences of primary healthcare in Cape Town, South Africa. BMC Geriatr [Internet]. 2019 Dec 4;19(1):98. Available from: https://bmcgeriatr.biomedcentral.com/articles/10.1186/s12877-019-1116-0

6. Pillemer K, Burnes D, Riffin C et al. E. Elder abuse: global situation, risk factors, and prevention strategies. Gerontol 2004;44469-78. 2016;56:196–205.

7. Evans JM, Kiran PR BOA. Activating the knowledge-to-action cycle for geriatric care in India. Heal Res Policy Syst. 2011;9(42).

8. Yaya S, Idriss-wheeler D, Sanogo NA, Vezina M, Bishwajit G. Self-reported activities of daily living , health and quality of life among older adults in South Africa and Uganda : a cross sectional study. 2020;1–11.

9. Smith M, Saunders R, Stuckhardt L et al. Imperative: Managing Rapidly Increasing Complexity. National Academies Press. National Academies Press (US). 2013.

10. Guarinoni M, Petrucci C, Lancia L et al. The Concept of Care Complexity: A Qualitative Study. J Public Heal Res. 2015;13(3).

11. Aging in Sub-Saharan Africa: Recommendations for Furthering Research. Aging in Sub-Saharan Africa. 2006.

12. Schatz E, Seeley J. Gender. Gender, ageing & carework in east and southern Africa: a review. Glob Public Heal. 2015;10:1185–200.

13. Fried LP, Tangen CM, Walston J et al. Frailty in older adults: evidence for a phenotype. J Gerontol Ser. 2001;56(3):2001.

14. Luciana Correia Alves, Yeda Aparecida de Oliveira Duarte JLFS. Factors Associated the Transitions in the Frailty States Among Elderly in Brazil 2006-2010. 2018. p. 1–16.

15. Espinoza SE, Jung I, Hazuda H. Frailty transitions in the San Antonio Longitudinal Study of Aging. J Am Geriatr Soc. 2012 Apr;60(4):652–60.

16. Uchmanowicz I, Chudiak A, Jankowska-pola B, Gobbens R. Hypertension and Frailty Syndrome in Old Age : Current Perspectives. Card Fail Rev. 2017;3(2):102–7.

17. Gobbens RJ, Assen MA Van, Luijkx KG, Schols JM. Testing an integral conceptual model of frailty. J Adv Nurs. 2011;1–14.

18. Theou O, O’Connell MDL, King-Kallimanis BL, O’Halloran AM, Rockwood K, Kenny RA. Measuring frailty using self-report and test-based health measures. Age Ageing. 2015 May;44(3):471–7.

19. Chamberlain AM, Sauver JLS, Jacobson DJ, Manemann SM, Fan C, Roger VL, et al. Social and behavioural factors associated with frailty trajectories in a population-based cohort of older adults. 2016;1–10.

20. Siriwardhana DD, Hardoon S, Rait G, Weerasinghe MC, Walters KR. Prevalence of frailty and prefrailty among community-dwelling older adults in low-income and middle- income countries : a systematic review and meta-analysis. BMJ Open. 2018;8:1–17.

21. Fairhall N, Kurrle SE, Sherrington C, Lord SR, Lockwood K, John B, et al. Effectiveness of a multifactorial intervention on preventing development of frailty in pre-frail older people : study protocol for a randomised controlled trial. 2015;1–8.

22. Kim C.O., Lee H.Y., Ho S.H., Park H.S. PCW. Effects of visiting prehabilitation program against functional decline in the frail elderly: A prospective randomized community trial. J Korean Gerontol. 2010;30:1293–309.

23. Lee I.S., Ko Y., Lee K.O. YES. Evaluation of the effects of a frailty preventing multi-factorial program concentrated on local communities for high-risk younger and older elderly people. J Korean Acad Community Heal Nurs. 2012;23:201–11.

24. Marcus-Varwijk AE, Peters LL, Visscher TLS, Smits CHM, Ranchor A V., Slaets JPJ. Impact of a Nurse-Led Health Promotion Intervention in an Aging Population: Results From a Quasi-Experimental Study on the “Community Health Consultation Offices for Seniors.” J Aging Health. 2020;32(1):83–94.

25. Kuhirunyaratn P, Prasomrak P, Jindawong B. Effects of a health education program on fall risk prevention among the urban elderly: A Quasi-experimental study. Iran J Public Health. 2019;48(1):103–11.

26. Viggars RJ, Finney A, Panayiotou B. Educational programmes for frail older people, their families, carers and healthcare professionals. Wien Klin Wochenschr. 2021;(September).

27. Thea Dunn, Julie Bliss IR. The impact of community nurse-led interventions on the need for hospital use among older adults: An integrative review. Int J Older People Nurs. 2021;

28. Goodman C, Davies SL, Dinan S, Tai SS, Iliffe S. Activity promotion for community-dwelling older people: a survey of the contribution of primary care nurses. Br J Community Nurs,. 2011;16(1):12–7.

29. Lee Jong-wook. Chronic Disease and Nursing : What ’ s the issue? 2005.

30. Song MS, Boo S. Effects of a nurse-led multicomponent intervention for frail older adults living alone in a community: a quasi-experimental study. BMC Nurs. 2022;21(1):1–9.

31. Markle-Reid M, Browne G, Gafni A. Nurse-led health promotion interventions improve quality of life in frail older home care clients: Lessons learned from three randomized trials in Ontario, Canada. J Eval Clin Pract. 2013;19(1):118–31.

32. Ha J, Park YH. Effects of a person-centered nursing intervention for frailty among prefrail community-dwelling older adults. Int J Environ Res Public Health. 2020;17(18):1–19.

33. Markle-Reid M, Weir R, Browne G, Roberts J, Gafni A, Henderson S. Health promotion for frail older home care clients. J Adv Nurs. 2006;54(3):381–95.

34. Rice H, Say R, Betihavas V. The effect of nurse-led education on hospitalisation, readmission, quality of life and cost in adults with heart failure. A systematic review. Patient Educ Couns [Internet]. 2018;101(3):363–74. Available from: http://www.ncbi.nlm.nih.gov/pubmed/29102442

35. Corley AG, Thornton CP, Glass NE. The Role of Nurses and Community Health Workers in Confronting Neglected Tropical Diseases in Sub-Saharan Africa: A Systematic Review. PLoS Negl Trop Dis. 2016;10(9):1–24.

36. Semachew A, Belachew T, Tesfaye T, Adinew YM. Predictors of job satisfaction among nurses working in Ethiopian public hospitals, 2014: institution-based cross-sectional study. Hum Resour Health [Internet]. 2017;15(1):31. Available from: http://www.ncbi.nlm.nih.gov/pubmed/28438214

37. PEPFAR F& I. The Global Nurse Capacity Producing and Maintaining a Skilled Nursing Workforce in Ethiopia. 2017.

38. Vellas B, Sourdet S. PREVENTION OF FRAILTY IN AGING. J Frailty Aging. 2017;6(4):174–7.

39. Cameron ID, Fairhall N, Langron C, Lockwood K, Monaghan N, Aggar C, et al. A multifactorial interdisciplinary intervention reduces frailty in older people : randomized trial. 2013;

40. Lohman M, Dumenci L, Mezuk B. Depression and Frailty in Late Life: Evidence for a Common Vulnerability. Journals Gerontol Ser B Psychol Sci Soc Sci [Internet]. 2016 Jul;71(4):630–40. Available from: https://academic.oup.com/psychsocgerontology/article-lookup/doi/10.1093/geronb/gbu180

41. Soysal P, Veronese N, Thompson T, Kahl KG, Fernandes BS, Prina AM, et al. Relationship between depression and frailty in older adults: A systematic review and meta-analysis. Ageing Res Rev [Internet]. 2017 Jul;36:78–87. Available from: https://linkinghub.elsevier.com/retrieve/pii/S1568163717300247

42. Frost R, Nair P, Aw S, Gould RL, Kharicha K, Buszewicz M, et al. Supporting frail older people with depression and anxiety: a qualitative study. Aging Ment Health [Internet]. 2020 Dec 1;24(12):1977–84. Available from: https://www.tandfonline.com/doi/full/10.1080/13607863.2019.1647132

43. Markle-reid M, Browne G. Conceptualizations of frailty in relation to older adults. J Adv Nurs. 2003;44(1):1–11.

44. Kivunja C. Distinguishing between theory, theoretical framework, and conceptual framework: A systematic review of lessons from the field. Int J High Educ. 2018;7(6):44–53.

45. Jabareen Y. Building a Conceptual Framework: Philosophy, Definitions, and Procedure. Int J Qual Methods. 2009;8(4):49–62.

46. David B. Hogan., Chris MacKnight HB et al. Models, definitions, and criteria of frailty. Aging Clin Exp Res. 2003;15(3):3–29.

47. Rockwood K, Mitnitski A. Frailty in Relation to the Accumulation of Deficits. J Gerontol Med Sci. 2007;62(7):722–7.

48. Ii WMB. A Conceptual Framework of Frailty : A Review. J Gerontol Med Sci. 2002;57(5):283–8.

49. Jhon E. et al. Frailty consensus: A call to action. J Am Med Dir Assoc. 2013;14(6):1–16.

50. Walston J, Hadley EC, Ferrucci L, Guralnik JM, Newman AB, Studenski SA, et al. Research agenda for frailty in older adults: Toward a better understanding of physiology and etiology: Summary from the American Geriatrics Society/National Institute on Aging research conference on frailty in older adults. J Am Geriatr Soc. 2006;54(6):991–1001.

51. Wang H, Wang J, Xie B, Liu B, Wang J. Multi-dimensional frailty and its risk factors among older residents in long-term care facilities in Shanghai, China. Int J Nurs Sci. 2021;8(3):298–303.

52. Gobbens RJJ, Luijkx KG, Wijnen-Sponselee MT, Schols JMGA. Towards an integral conceptual model of frailty. J Nutr Health Aging. 2009 Mar;14(3):175–81.

53. Clegg A et al. Frailty in elderly people. Lancet. 2013;381(9868):752–62.

54. Morley J. et al. Frailty consensus: a call to action. J Am Med Dir Assoc. 2013;14(6):392.

55. Bergman H et al. Frailty: an emerging research and clinical paradigm--issues and controversies. The Journals of Gerontology Series. Biol Sci Med Sci. 2007;62(7):731–7.

56. Fried LP et al. Frailty in older adults: evidence for a phenotype. The Journals of Gerontology Series A: Biol Sci Med Sci. 2001;56(3):146–56.

57. Bieniek J, Wilczyński K, Szewieczek J. Fried frailty phenotype assessment components as applied to geriatric inpatients. Clin Interv Aging. 2016;11:453–9.

58. Alves S, Teixeira L, Ribeiro O, Paúl C. Examining Frailty Phenotype Dimensions in the Oldest Old. Front Psychol. 2020;11(March):1–8.

59. Gobbens R.J., Luijkx K.G. W-SMT& SJM. towards an integral conceptual model of frailty. J Nutr Health Aging. 2010;14(175–181).

60. Collard RM, Boter H, Schoevers RA OVR. Prevalence of frailty in community- dwelling older persons: A systematic review. J Am Geriatr Soc. 2012;60:1487–92.

61. Levers M-J, Estabrooks CA, Ross Kerr JC. Factors contributing to frailty: literature review. J Adv Nurs. 2006 Nov;56(3):282–91.

62. Xie B, Larson JL, Gonzalez R, Pressler SJ, Lustig C, Arslanian-Engoren C. Components and Indicators of Frailty Measures: A Literature Review. J frailty aging. 2017;6(2):1–7.

63. Ravaglia G, Forti P, Lucicesare A, Pisacane N, Rietti E, Patterson C. Development of an easy prognostic score for frailty outcomes in the aged. Age Ageing. 2008;37(2):161–6.

64. Oostrom SH Van, A DL Van Der, Rietman ML, Picavet HSJ, Lette M, Verschuren WMM, et al. A four-domain approach of frailty explored in the Doetinchem Cohort Study. BMC Geriatr. 2017;17(196):1–12.

65. F. Béland, H. Bergman, P. Lebel, L. Dallaire, J. Fletcher A. Integrated services for frail elders (SIPA): a trial of a model for Canada. Can J Aging. 2006;25(1):2006.

66. Gobbens RJ et al. Towards an integral conceptual model of frailty. J Nutr Health Aging. 2010;14(3):175–81.

67. WHO. The World Health Organization Quality of Life (WHOQOL). 2012. p. 1–12.

68. Gobbens RJ, Luijkx KG, Wijnen-Sponselee MT et al. Toward a conceptual definition of frail community dwelling older people. Nurs Outlook. 2010;58:76–86.

69. Mousavi Sisi M, Shamshirgaran SM, Rezaeipandari H, Matlabi H. Multidimensional Approach to Frailty among Rural Older People: Applying the Tilburg Frailty Indicator. Elder Heal J. 2019;5(2):92–101.

70. Des Jarlais et. Standards for reporting non-randomized evaluations of behavioral and public health interventions: The TREND statement. Am J Public Health. 2004;94(3):361–6.

71. Birhanie G, Melese H, Solomon G, Fissha B, Teferi M. Fear of falling and associated factors among older people living in Bahir Dar City, Amhara, Ethiopia- a cross-sectional study. BMC Geriatr [Internet]. 2021 Dec 21;21(1):586. Available from: https://bmcgeriatr.biomedcentral.com/articles/10.1186/s12877-021-02534-x

72. Argaw MD, Desta BF, Bele TA, Ayne AD. Improved performance of district health systems through implementing health center clinical and administrative standards in the Amhara region of Ethiopia. BMC Health Serv Res [Internet]. 2019 Dec 19;19(1):127. Available from: https://bmchealthservres.biomedcentral.com/articles/10.1186/s12913-019-3939-y

73. Bahir Dar city administration health office. Bahir Dar City Administration demographic profile. 2021. p. 1.

74. Faul F, Erdfelder E, Buchner A LA. Statistical power analyses using G*power 3.1: tests for correlation and regression analyses. Behav Res Methods [Internet]. 2009;41(4):1149–60. Available from: https://doi.org/10.3758/BRM.41.4.1149

75. Jemal K, Hailu D, Tesfa B, Lama T, Kinati T, Mengistu E. Geriatric depression and quality of life in North Shoa Zone, Oromia region: a community cross-sectional study. Ann Gen Psychiatry. 2021;20(1):1–10.

76. Federal Negarit Gazeta of the Federal Democratic Republic Of Ethiopia. A Proclamation to Provide For Public Servants’ Pension: Proclamation No. 714/2011. 2011.

77. HelpAge International. Vulnerability of Older People in Ethiopia: The Case of Oromia, Amhara and SNNP Regional States. 2013.

78. Federal Ministry of Health, USAID & JOHN SNO W I. Ethiopian Urban Health Extension Program [Internet]. 2018. p. 4. Available from: https://publications.jsi.com/JSIInternet/Inc/Common/_download_pub.cfm?id=22119&lid=3

79. Tilahun H, Fekadu B, Abdisa H, Canavan M, Linnander E, Bradley EH, et al. Ethiopia’s health extension workers use of work time on duty: Time and motion study. Health Policy Plan. 2017;32(3):320–8.

80. Lee IFK, Yau FN, Yim SSH, Lee DTF. Evaluating the impact of a home-based rehabilitation service on older people and their caregivers: A matched-control quasi-experimental study. Clin Interv Aging. 2018;13:1727–37.

81. Haider S, Dorner TE, Luger E, Kapan A, Titze S, Lackinger C, et al. Impact of a home-based physical and nutritional intervention program conducted by lay-volunteers on handgrip strength in prefrail and frail older adults: A randomized control trial. PLoS One. 2017;12(1):1–15.

82. Yu R, Tong C, Ho F, Woo J. Effects of a Multicomponent Frailty Prevention Program in Prefrail Community-Dwelling Older Persons: A Randomized Controlled Trial. J Am Med Dir Assoc [Internet]. 2020 Feb;21(2):294.e1-294.e10. Available from: https://linkinghub.elsevier.com/retrieve/pii/S1525861019306401

83. Wong AKC, Wong FKY. The psychological impact of a nurse-led proactive self-care program on independent, non-frail community-dwelling older adults: A randomized controlled trial. Int J Nurs Stud [Internet]. 2020 Oct;110:103724. Available from: https://linkinghub.elsevier.com/retrieve/pii/S0020748920302108

84. Dedeyne L, Deschodt M, Verschueren S, Tournoy J, Gielen E. Effects of multi-domain interventions in (pre)frail elderly on frailty, functional, and cognitive status: a systematic review. Clin Interv Aging [Internet]. 2017;12:873–96. Available from: http://www.ncbi.nlm.nih.gov/pubmed/28579766

85. Gobbens RJ, Uchmanowicz I. Assessing frailty with the tilburg frailty indicator (TFI): A review of reliability and validity. Clin Interv Aging. 2021;16:863–75.

86. Ma L. Current Situation of Frailty Screening Tools for Older Adults. J Nutr Heal Aging. 2019;23(1):111–8.

87. Gobbens RJ, van Assen MA, Luijkx KG, Wijnen-Sponselee MT SJ. The Tilburg frailty indicator: psychometric properties. J Am Med Dir Assoc. 2010;11(5):2010.

88. Uchmanowicz I, Jankowska-Polańska B, Łoboz-Rudnicka M, Manulik S, Łoboz-Grudzień K, Gobbens RJJ. Cross-cultural adaptation and reliability testing of the Tilburg frailty indicator for optimizing care of polish patients with frailty syndrome. Clin Interv Aging. 2014;9:997–1001.

89. Hailemariam H, Singh P, Fekadu T. Evaluation of mini nutrition assessment (MNA) tool among community dwelling elderly in urban community of Hawassa city, Southern Ethiopia. BMC Nutr. 2016;2(11):1–6.

90. Yordanos Mezemir, Gudina Egata DG and AL. Nutritional status and associated factors among community-dwelling elderly. Nutr Diet Suppl. 2020;12:1–11.

91. Abate T, Mengistu B, Atnafu A, Derso T. Malnutrition and its determinants among older adults people in Addis Ababa, Ethiopia. BMC Geriatr. 2020;20(1):1–9.

92. Institute NN. Nutrition screening - a guide to completing the mini nutritional assessment. 2011.

93. Phillips MB, Foley AL, Barnard R, Isenring EA, Miller MD. Nutritional screening in community-dwelling older adults: A systematic literature review. Asia Pac J Clin Nutr. 2010;19(3):440–9.

94. Mesfin Agachew and DH. Faculty of Health Sciences School of Public Health [Internet]. 2017. Available from: http://146.141.12.21/handle/10539/14452

95. John E and David R. Geriatric nutrition Vol. 8. 2007, taylor and francis group: new york. 602. Taylor Fr Gr. 2007;8(602):2007.

96. Amha H, Fente W, Sintayehu M, Tesfaye B, Yitayih M. Depression and associated factors among old age population in Dega damot district, North West Ethiopia. A cross-sectional study. J Affect Disord Reports. 2020;2(October):100034.

97. Girma M, Hailu M, Wakwoya DA, Yohannis Z, Ebrahim J. Geriatric Depression in Ethiopia: Prevalence and Associated Factors. J Psychiatry. 2016;20(1):1–5.

98. Habte E, Tekle T. Cognitive Functioning among Elders with Symptoms of Depression: The Case of Two Selected Institutionalized Care Centers in Addis Ababa, Ethiopia. Heal Sci J. 2018;12(03):1–7.

99. Sarason IG, Sarason BR, Shearin EN and PG. A brief measure of social support: Practical and theoretical implications. J Soc Pers Relat. 1987;4(4):497–510.

100. Nursing THI for G. Katz Index of Independence in Activities of Daily Living. Best Pract Nurs Care to Older Adults,. 2007;25(2):8–9.

101. Donna McCabe. Katz Index of Independence in Activities of Daily Living (ADL). Director [Internet]. 2019;8(2):1–2. Available from: https://hign.org/consultgeri/try-this-series/katz-index-independence-activities-daily-living-adl%0A

102. Shelkey M, Wallace M. Katz Index of Independence in Activities of Daily Living (ADL). Director [Internet]. 2000;8(2):72–3. Available from: https://www.researchgate.net/publication/12246793_Katz_Index_of_Independence_in_Activities_of_Daily_Living_ADL

103. Birhanie G, Melese H, Solomon G, Fissha B, Teferi M. Fear of falling and associated factors among older people living in Bahir Dar City, Amhara, Ethiopia- a cross-sectional study. BMC Geriatr [Internet]. 2021;21(1):1–12. Available from: https://doi.org/10.1186/s12877-021-02534-x

104. Reba K, Birhane BW, Gutema H. Validity and reliability of the Amharic version of the world health organization’s quality of life questionnaire (whoqolbref) in patients with diagnosed type 2 diabetes in felege hiwot referral hospital, Ethiopia. J Diabetes Res. 2019;2019:1–7.

105. Temesgen WA. Progress of recovery and its associated factors in recent onset Psychosis: a mixed-methods study Worku Animaw Temesgen. 2020.

106. World Health Organization (WHO). WHOQOL_ Measuring Quality of Life [Internet]. WHO. 2012. p. 1–88. Available from: https://apps.who.int/iris/rest/bitstreams/110129/retrieve

107. Sørensen MP, Ravn T, Marušić A, Elizondo AR, Kavouras P, Tijdink JK, et al. Strengthening research integrity: which topic areas should organisations focus on? Humanit Soc Sci Commun. 2021;8(1):1–15.

108. Impact R for development. Effective and ethical research and evaluation [Internet]. 2022. p. 1–5. Available from: https://rdinetwork.org.au/effective-ethical-research-evaluation/ethical-practice-starter- kit/research-merit-integrity/

109. Australian Government: National Health and Medical Research Council. Conduct in human research national statement on ethical conduct in human research [Internet]. Vol. 2007. 2018. 104 p. Available from: www.nhmrc.gov.au/guidelines/publications/e72%0Ahttps://www.nhmrc.gov.au/about-us/publications/national-statement-ethical-conduct-human-research-2007-updated-2018

110. University of Michigan. Research ethics and compliant: Informed Consent Guidelines & Templates [Internet]. 2022. p. 1–4. Available from: https://research-compliance.umich.edu/informed-consent-guidelines

111. The Australian National University. Information Sheets & Consent Forms [Internet]. 2022. p. 1–4. Available from: https://services.anu.edu.au/research-support/ethics-integrity/getting-ethics-approval/information- sheets-consent-forms%0A

112. University of Nevada Research. Research integrity: Maintaining Data Confidentiality [Internet]. 2021. p. 6. Available from: https://www.unr.edu/research-integrity/human-research/human-research-protection-policy- manual/410-maintaining-data-confidentiality

113. Bos J. Research Ethics for Students in the Social Sciences [Internet]. Research Ethics for Students in the Social Sciences. 2020. 1–287 p. Available from: https://link.springer.com/chapter/10.1007/978-3-030-48415-6_7

114. Marina Marcus, M. Taghi Yasamy et al. A Global Public Health Concern: WHO Department of Mental Health and Substance Abuse. 2012.

115. J. Cordes. “Depression” in Encyclopedia of Sciences and Religions,. 2013.

116. Debra Fulghum Bruce. Depression in Older People,. WebMD. 2020. p. 2020.

117. WHO. Depression. Vol. 4. 2021.

118. World Federation for Mental Health. Depression: A global crisis? 2012.

119. Woledesenbet MA, Shumet Mekonen S, Sori LM, Abegaz TM. Epidemiology of Depression and Associated Factors among Asthma Patients in Addis Ababa, Ethiopia. Psychiatry J. 2018;2018:1–7.

120. WHO. Integrating mental health into primary care-A global perspective. Geneva, Switzerland:; 2008.

121. World Health Organization (WHO). Depression. 2021. p. 1–3.

122. Paukert LA, LeMaire A CA. Predictors of depression episodes in older veterans with heart failure. Aging Ment Heal. 2009;13(4):601–10.

123. Quan CH, Rong BD, Chan ZL, Rong JY XO. Chronic diseases and risk for depression in old age: A meta- analysis of published literature. Ageing Res Rev. 2009;10:1016–27.

124. Institute of Health Metrics and Evaluation. Institute of Health Metrics and Evaluation. Global Health Data Exchange (GHDx). 2021.

125. Mirkena Y, Reta MM, Haile K, Nassir Z, Sisay MM. Prevalence of depression and associated factors among older adults at ambo town, Oromia region, Ethiopia. BMC Psychiatry. 2018;18(1):1–7.

126. National Institute of Health. Depression and Older Adults. 2022. p. 1–4.

127. Sparrow, E. P., & Erhardt D. How Do I Know If Is Adhd? Essentials of ADHD assessment for children and adolescents. 2014.

128. Fiske A, Wetherell JL GM. Depression in older adults. Annu Rev Clin Psychol. 2009;5:363–89.

129. WHO. Depression and Other Common Mental Disorders: Global Health Estimates, Geneva. 2017.

130. Peltzer K, Phaswana-Mafuya N. Depression and associated factors in older adults in South Africa. Glob Health Action. 2013;6(1):1–9.

131. Thapa SB, Martinez P, Clausen T. Depression and its correlates in South Africa and Ghana among people aged 50 and above: Findings from the WHO study on global ageing and adult health. African J Psychiatry (South Africa). 2014;17(6):1–11.

132. Gureje O, Kola L AE. Epidemiology of major depressive disorder in elderly Nigerians in the Ibadan Study of Ageing: a community-based survey. Lancet. 2007;370:957–64.

133. Bitew T. Prevalence and risk factors of depression in Ethiopia: a review. Ethiop J Health Sci. 2014;24(2):161–9.

134. Hailemariam S, Tessema F, Asefa M, Tadesse H, Tenkolu G. The prevalence of depression and associated factors in Ethiopia: findings from the National Health Survey. Int J Ment Health Syst. 2012;6:1–11.

135. Moledina SM, Bhimji KM, Manji KP. Prevalence and Associated Factors of Depression in an Asian Community in Dar es Salaam, Tanzania. Psychiatry J. 2018;2018:1–5.

136. Yunming L, Changsheng C, Haibo T, Wenjun C, Shanhong F, Yan M et al. Prevalence and risk factors for depression in older people in Xi’an China: a community-based study. Int J Geriatr Psychiatry. 2012;27:31–9.

137. Li N, Pang L, Chen G, Song X, Zhang J ZX. Risk factors for depression in older adults in Beijing. Can J Psychiatry. 2011;56:466–73.

138. Misganaw A, Melaku YA, Tessema GA, Deribew A, Deribe K, Abera SF et al. National disability-adjusted life years (DALYs) for 257 diseases and injuries in Ethiopia, 1990–2015: findings from the global burden of disease study 2015. Popul Heal Metrics. 2017;15(1):28.

139. Edmealem A, Olis CS. Factors Associated with Anxiety and Depression among Diabetes, Hypertension, and Heart Failure Patients at Dessie Referral Hospital, Northeast Ethiopia. Behav Neurol. 2020;2020.

140. Mossie TB, Berhe GH, Kahsay GH, Tareke M. Prevalence of depression and associated factors among diabetic patients at Mekelle City, North Ethiopia. Indian J Psychol Med. 2017;39(1):52–8.

141. Gebre BB, Anand S, Assefa ZM. Depression and Its Predictors among Diabetes Mellitus Patients Attending Treatment in Hawassa University Comprehensive Specialized Hospital, Southern Ethiopia. J Diabetes Res. 2020;2020.

142. Asmare Y, Ali A. Magnitude and Associated Factors of Depression Among People With Hypertension in Addis Ababa , Ethiopia : A Hospital Based Cross-Sectional Study. Res Sq. 2021;

143. Mulat N, Gutema H, Wassie GT. Prevalence of depression and associated factors among elderly people in Womberma District, north-west, Ethiopia. BMC Psychiatry. 2021;21(1):1–9.

144. Yimer YM, Buli MB, Nenko G, Mirkena Y, Kassew T. The prevalence and determinant factors of self-reported depressive symptoms among elderly people with visual impairment attending an outpatient clinic in Ethiopia. Clin Optom. 2021;13:63–72.

145. Moher D, Liberati A, Tetzlaff J, Altman DG. Preferred reporting items for systematic reviews and meta-analyses: The PRISMA statement. BMJ. 2009;339(7716):332–6.

146. Medicare I of M (US) C to D a S for QR and A in, Lohr KN. The Elderly Population. National Academies Press (US); 1990. 12 p.

147. Orimo H, Ito H, Suzuki T, Araki A, Hosoi T, Sawabe M. Reviewing the definition of “elderly.” Geriatr Gerontol Int. 2006;6(3):149–58.

148. Hoy D, Brooks P, Woolf A, Blyth F, March L, Bain C, et al. Assessing risk of bias in prevalence studies: Modification of an existing tool and evidence of interrater agreement. J Clin Epidemiol. 2012;65(9):934–9.

149. The Joanna Briggs Institute Critical Appraisal tools for use in JBI Systematic Reviews. Checklist for Prevalence Studies. 2016.

150. Adane T, Getawa S. Anaemia and its associated factors among diabetes mellitus patients in Ethiopia : A systematic review and meta- ­ analysis. Endocrinol Diabetes Metab. 2021;1–10.

151. JBI. Joanna Briggs Institute-System for the Unified Management, Assessment and Review of Information (JBI-SUMARI). [Online software program]. Adelaide; JBI; Copyright 2016. 2016.

152. Lin L, Chu H. Quantifying publication bias in meta-analysis. Biometrics. 2018;74(3):785–94.

153. Huedo-Medina TB, Sánchez-Meca J, Marín-Martínez F, Botella J. Assessing heterogeneity in meta-analysis: Q statistic or I 2 Index? Psychol Methods. 2006;11(2):193–206.

154. Lee YH. Overview of the Process of Conducting Meta-analyses of the Diagnostic Test Accuracy. J Rheum Dis. 2018;25(1):3.

155. Rücker G, Schwarzer G, Carpenter JR, Schumacher M. Undue reliance on I 2 in assessing heterogeneity may mislead. BMC Med Res Methodol. 2008;8(79).

156. Egger M, Smith GD, Schneider M, Minder C. Bias in meta-analysis detected by a simple, graphical test. BMJ. 1997 Sep;315(7109):629–34.

157. Soeken, Karen L.; Sripusanapan A. Nursing research: Assessing Publication Bias in Meta- Analysis. Rec Manag J. 2003;52(1):57–60.

158. Mezemir Y, Egata G, Geset D LA. Nutritional Status and Associated Factors Among the Community-Dwelling Elderly Population in. Nutr Diet Suppl. 2020;12:289–99.

159. Bekele GT, Allene MD, Getnet MG, Hunegnaw MT, Janakiraman B. Assessing falls risk and associated factors among urban community dwellers older adults in Gondar town, Northwest Ethiopia 2019: A cross sectional study. Int J Surg Open. 2020;24:177–84.

160. Abdu AO, Yimamu ID, Kahsay AA. Predictors of malnutrition among older adults aged above 65 years in eastern Ethiopia: neglected public health concern. BMC Geriatr. 2020;20(1):1–11.

161. Higgins JPT, Thompson SG. Quantifying heterogeneity in a meta-analysis. Stat Med. 2002 Jun;21(11):1539–58.

162. Sterne JA, Egger M. Funnel plots for detecting bias in meta-analysis: guidelines on choice of axis. J Clin Epidemiol. 2001 Oct;54(10):1046–55.

163. Redina-Gobioff et al. Detecting Publication Bias in Random Effects Meta-Analysis: An Empirical Comparison of Statistical Methods. 2006. p. 1–6.

164. Barua A, Ghosh M, Kar N, Basilio M. Prevalence of depressive disorders in the elderly. Ann Saudi Med. 2011;31(6):620–4.

165. Assil SM, Zeidan ZA. Prevalence of depression and associated factors among elderly Sudanese : a household survey in Khartoum State. EMHJ. 2013;19(5):435–40.

166. Nuworza. Prevalence of geriatric depression in a community sample in Ghana : Analysis of associated risk and protective factors. Arch Gerontol Geriatr. 2018;78:171–6.

167. Elkhawaga GO, Sarraf BB. Depression and its associated factors among elderly : A community-based study in Egypt. Arch Gerontol Geriatr. 2018;77:1–10.

168. Adams DJ, Ndanzi T, Rweyunga AP, George J, Mhando L, Ngocho JS, et al. Aging & Mental Health Depression and associated factors among geriatric population in Moshi district council , Northern Tanzania. Aging Ment Health. 2021;25(6):1–10.

169. Sarokhani D, Parvareh M, Dehkordi AH, Sayehmiri K. Prevalence of Depression among Iranian Elderly : Systematic Review and Meta-Analysis. Iran J Psychiatry. 2018;13(1):55–64.

170. Akosile et al. Depression , functional disability and quality of life among Nigerian older adults : Prevalences and relationships. Arch Gerontol Geriatr. 2018;74:39–43.

171. Igbokwe CC, Ejeh VJ, Agbaje OS, Ifeanachor P, Umoke C, Iweama CN, et al. Prevalence of loneliness and association with depressive and anxiety symptoms among retirees in Northcentral Nigeria : a cross-sectional study. BMC Geriatr. 2020;20(153):1–10.

172. gang hao et al. Social participation and perceived depression among elderly population in South Africa. Clin Interv Aging. 2017;12:971–6.

173. Mckinnon B, Harper S, Moore S. The relationship of living arrangements and depressive symptoms among older adults in sub-Saharan Africa. BMC Public Health. 2013;13:1–9.

174. Mlaki DA, Asmal L, Paddick S, Gray WK, Dotchin C. Prevalence and associated factors of depression among older adults in rural. Int J Geriatr Psychiatry. 2021;36(10):1559–66.

175. Ndetei DM. Prevalence and determinants of depression among patients under the care of traditional health practitioners in a Kenyan setting : Policy implications. Transcult Psychiatry. 2017;54(3):1–8.

176. Peltzer K, Phaswana-mafuya N. Depression and associated factors in older adults in South Africa. Glob Heal Action. 2013;18:23336621.

177. Dao ATM, Nguyen VT, Nguyen H V. Urban Vietnam. Biomed Res Int. 2018;2018:1–10.

178. F.B. Van der Wurff et al. Prevalence and risk-factors for depression in elderly Turkish and Moroccan migrants in the Netherlands. J Affect Disord. 2004;83(1):2004.

179. Badrasawi M, Zidan S. Prevalence and correlates of depressive symptoms in older people in the West Bank , Palestine : cross-sectional study. EMHJ. 2021;27(3):1–9.

180. Chalise HN. Depression among elderly living in Briddashram ( old age home ). Adv Aging Res. 2014;3(1):6–11.

181. Etal JRM. Depression among older people in Europe : the EURODEP studies. World Psychiatry 31. 2004;3(1):45–9.

182. Sherina MS, Rampal L, Mustaqim A. The Prevalence of Depression Among the Elderly in. Med J Malaysia. 2004;59(June 2014):1–6.

183. Ryuta FUKUNAGA et al. Living alone is associated with depression among the elderly in a rural community in Japan. Psychogeriatrics ©. 2012;12:179–85.

184. Li N, Pang L, Chen G, Song X, Zhang J. Risk Factors for Depression in Older Adults in Beijing. Can J Psychiatry. 2020;56(8):1–8.

185. Lloyd-sherlock P, Agrawal S, Amoakoh-coleman M, Adom S. Old age and depression in Ghana : assessing and addressing diagnosis and treatment gaps. Glob Health Action. 2019;12(1).

186. Aly HY, Hamed AF, Mohammed NA. governorate. Saudi Med J. 2018;39(2):185–90.

187. Panza F, Frisardi V, Capurso C, D’Introno A, Colacicco AM, Imbimbo BP, et al. Late-life depression, mild cognitive impairment, and dementia: possible continuum? Am J Geriatr Psychiatry. 2010 Feb;18(2):98–116.

# Appendices

## Appendix A: Literature review

## Appendix A 1: Review one

Manuscript ID: NOP-2022-Mar-0362

Title: The extent of frailty in older adults living in Africa: Systematic review and meta-analysis

**The extent of frailty in older adults living in Africa: Systematic review and meta-analysis**

| Journal: | *Nursing Open* |
| --- | --- |
| Manuscript ID | Draft |
| Wiley - Manuscript type: | Review Article |
| Search Terms: | Aged Care, Epidemiology, Geriatric, Gerontology |
| Abstract: | Aim: This study was aimed to determine pooled prevalence of frailty among older adults in African and to identify factors associated with frailty.  Design: systematic review and meta-analysis.  Methods: Data were extracted using a standardized data extraction checklist, and analysis was conducted using STATA 14 statistical software. The Cochran Q test and inverse variance weighting were used to assess heterogeneity.  Results: The overall pooled prevalence of frailty among older adults in Africa was 38.64% (95% [CI]: 26.11, 51.17). Subgroup analysis indicated the highest prevalence of frailty in West Africa, with a prevalence of 57.23% (95% CI: 45.47, 68.99). The presence of a  comorbidity (AOR = 1.6, 95% CI: 1.77, 2.18) was associated with a status of frailty among older adults.  Conclusions: The finding revealed that frailty is highly prevalent among older adults in Africa. Moreover, further research that addresses the health of frail older adults in Africa must be conducted. |
|  | |

**Abstract**

**Aim:** This study was aimed to determine pooled prevalence of frailty among older adults in African and to identify factors associated with frailty.

**Design:** systematic review and meta-analysis.

**Methods**: Data were extracted using a standardized data extraction checklist, and analysis was conducted using STATA 14 statistical software. The Cochran Q test and inverse variance weighting were used to assess heterogeneity.

**Results:** The overall pooled prevalence of frailty among older adults in Africa was 38.64% (95% [CI]: 26.11, 51.17). Subgroup analysis indicated the highest prevalence of frailty in West Africa, with a prevalence of 57.23% (95% CI: 45.47, 68.99). The presence of a comorbidity (AOR = 1.6, 95% CI: 1.77, 2.18) was associated with a status of frailty among older adults.

**Conclusions:** The finding revealed that frailty is highly prevalent among older adults in Africa. Moreover, further research that addresses the health of frail older adults in Africa must be conducted.

**Keywords:** Frailty, elderly, aged, older adults, Africa

**Introduction**

Globally, the fastest growth rate of older individuals will occur in Africa (Kamiya 2016). According to a 2017 United Nations (UN) report on population aging, the African population aged ≥60 years is projected to increase more than threefold between 2017 and 2050, from 69 to 225 million people. The lack of a responsible body for the comprehensive data collection and monitoring of the older population at continental and national levels is an indication of how little attention is given to older people (African Union 2020).

Frailty is becoming a major health concern of aging societies (Luciana Correia Alves, Yeda

Aparecida de Oliveira Duarte 2018). The literature in developed nations has indicated that frailty is a prime modulator of an individual’s health trajectory later in life. Therefore, understanding the extent of frailty constitutes a basic awareness of physiological reserves in old age (Ribeiro et al. 2019). Many older adults with frailty struggle to manage their condition. More than 75% of older adults have at least one chronic condition that requires continuous care (Uchmanowicz et al. 2017). Frailty coupled with chronic conditions affect the quality of daily life of the aging population.

**Background**

Frailty is a geriatric clinical syndrome that increases an individual’s vulnerability to many adverse outcomes, including falls, disabilities, institutionalization, and mortality; it is caused by the cumulative decline of multiple physiological systems and decreased resistance to stressors (Duzgun, Ustundag, and Karadakovan 2021; Fried et al. 2001). Because of limitations in basic daily activities, decreased independence, and progressive disabilities, frail older adults have difficulty maintaining their daily lifestyle (Espinoza, Jung, and Hazuda 2012), which diminishes their overall well-being and quality of life.

Frailty is not caused by a single factor but is rather commonly affected by multiple factors that can be interrelated or independent (Espinoza et al. 2012; Luciana Correia Alves, Yeda Aparecida de Oliveira Duarte 2018). Frailty and chronic diseases represent the clinical manifestations of the accumulation of biological deficits that occur with aging. However, assessment of frailty is yet to be included in routine clinical practice.

Studies conducted in numerous settings in diverse countries have indicated various degree of frailty among older adults. Some studies recruited older adults from community settings, and others recruited older adults from institutional settings. Additionally, multiple measurement tools have been used to determine the outcome variable.

In the literature, female sex, advanced age, living alone, low education level, low income level, poor self-rated health, cognitive impairment, obesity, and multimorbidity are factors associated with frailty in older adults (Ávila-Funes et al. 2009; Chen CY, Wu SC, Chen LJ 2010). Studies suggested that frailty occurs frequently in older people and is associated with adverse outcomes (Vu et al. 2017). Frailty is correlated with morbidity and mortality in individuals with chronic conditions, and the recognition of frailty can help healthcare providers determine prognoses, procedural risks, and treatments (Graziano Onder, Davide L 2018). Recognizing the needs of frail older adults will eliminate many of the problems they encounter and improve their quality of life, safety, and overall health (Maresova et al. 2019).

A narrative review of frailty in developing countries published in 2015 indicated that the relevant studies are limited and revealed that frailty occurs more frequently in developing countries than in developed countries (Nguyen, Cumming, and Hilmer 2015). A meta-analysis of studies published in 2021 across 62 countries on the prevalence of frailty reported an increasing global prevalence of frailty (O’Caoimh et al. 2021). However, these reviews and meta-analyses contained only one study conducted in Africa.

Although considerable research on frailty has been conducted across other continents, research on frailty in Africa is quite fragmented. Studies also revealed that Africans have a higher risk of frailty than people of European descent do (Papathanasiou et al. 2021). The existing studies about frailty are not meticulous; therefore, they do not provide a comprehensive estimate of the frailty of older adults living in Africa.

Hence, providing and summarizing existing epidemiological data and evidence on frailty among older adults in the African context are critical for healthcare policymakers and high-level government agencies. This systematic review summarized the available epidemiological data on frailty and measured the pooled prevalence of frailty on the African continent through a meta- analysis.

**Design**

This review was conducted according to standards outlined in the Preferred Reporting Items for Systematic Reviews and Meta-Analyses (PRISMA) statement (Moher et al. 2009).

**Ethics**

This systematic review summarized the existing epidemiological data and evidence on frailty among older adults in the African context. The results are to be disseminated through publication in a peer-reviewed journal. Data used were obtained from previously published studies, and there- fore, ethics approval was not necessary to be obtained for this systematic review and meta-analysis. **Methods**

**Reporting**

This review was registered at the National Institute for Health Research (NIHR) International prospective register of systematic reviews with a PROSPERO ID of CRD42020152299. The Preferred Reporting Items of Systematic Reviews and Meta-Analysis (PRISMA) checklist was used to report the results of this review (Appendix 1), and the PRISMA flowchart (Figure 1) was used to illustrate the selection process of the studies included in this analysis (Moher et al. 2009).

**Search strategy and selection criteria**

**Search strategy**

This systematic review and meta-analysis was conducted to estimate the prevalence of frailty in older adults living in Africa. The PubMed, Web of Science, SCOPUS, CINAHL, Science Direct, African Index Medicus, African Journals Online, WHO Global Health Library, and HINARI databases were searched between August 22 and October 22, 2021, using the following search terms: “extent,” “magnitude,” “prevalence,” “epidemiology,” “factors,” “predictors,” “frailty,”

“frail,” “frail elderly,” “frail older adults,” “frail geriatric,” “frail oldest people,” “frail older people,” “frail aged people,” and “Africa.” Search strings were developed using “AND” and “OR” Boolean operators. The reference lists of the retrieved articles were also examined for additional relevant studies to be included in the current review. Manual searches were also performed using the name of each African country.

**Selection criteria**

Original research articles that reported the prevalence of frailty in older adults living in Africa were included. Studies were not limited by the time of the study or the year of publication but must be written or published in English. Thesis reports, dissertations, and proceedings and conference reports that stated the outcome variable were also considered in our search. An article was excluded if it only included partial text or was a program evaluation, review article, conference abstract, editorial, or commentary.

**Participants**

Study participants were older adults aged ≥60 years.

**Outcomes**

Studies were only included if they contained information on frailty prevalence in older adults living in African countries.

**Context**

The current review considered studies conducted with older adults who lived within a community or rural area, visited healthcare institutions, were admitted to hospitals, or received services in long- term care settings or nursing homes.

**Quality assessment and data extraction**

To ensure methodological quality, two authors (ASK and SCL) independently assessed the quality of the included studies. To assess methodological quality, we used the JBI critical appraisal checklist for cross-sectional (Aromataris et al. 2020) and cohort (JBI 2016) studies. Studies scoring

≥50% according to the quality assessment checklist criteria were deemed low risk (Mulualem, Wondim, and Woretaw 2019). The authors methodically appraised all included studies before analysis. Data were extracted from the eligible studies by two reviewers (ASK and SCL). A standard data extraction form was designed and piloted before being applied to each study. The extracted data included specific details on the authors, year, country, continental region, setting, study design, sampling method, number and age of participants, reported comorbidities, and outcome of interest for the review question. Any disagreements between the reviewers were resolved through discussions with a third reviewer (HCC).

**Data synthesis**

The collected data were analyzed to determine the pooled prevalence of frailty among African older adults using statistical meta-analysis. The data were analyzed using STATA version 14.0 (STATA Corporation, College Station, TX, USA) software. Heterogeneity across studies was verified through inverse variance (*I*2) weighting and the Cochran Q test (Lin and Chu 2018a), (Higgins and Thompson 2002), (Rücker et al. 2008). Cutoffs of 25%, 50%, and 75% were used to denote low, moderate, and severe heterogeneity, respectively (Huedo-Medina et al. 2006). The original studies were summarized and are presented in a table and forest plot. Because the test statistics revealed considerable heterogeneity among the studies (*I*2 = 97.5%, P < 0.001), a random effects model was used to estimate the Der Simonian and Laird pooled effect. The funnel plot was examined for the absence of publication bias, as a subjective assessment. Potential publication bias was also

objectively evaluated using Egger's regression and Begg's rank tests with a 5% significance level (Lin and Chu 2018b). The test results indicated no significant publication bias (β = 2.07, SE = 2.04, P = 0.337). Additionally, to minimize the random variations between the point estimates of the original studies, subgroup analysis was conducted according to the regions in which the studies were conducted, study settings, and presence of comorbidities in relation to the outcome variable.

**Search results**

We retrieved 317 articles through a search of electronic databases and through manual searches. A total of 81 duplicate articles were excluded, and an additional 192 articles were excluded after assessing their titles and abstracts. The remaining 44 articles were further evaluated based on preset eligibility criteria, and 11 studies were ultimately eligible for this systematic review and meta- analysis.

**Description of the included studies**

All 11 studies were conducted between 2012 and 2021. Of the five studies in North Africa, one was conducted in Tunisia (Hammami et al 2020), and four were conducted in Egypt (Ebeid et al 2016; Khater & Mousa 2012; Naeem et al 2020; Sabbour et al 2018). Four studies were conducted in East Africa (K. Gray et al 2017; Lewis et al 2018b, 2018a, 2019), and two (Adebusoye et al 2019; Ajayi et al 2021) were conducted in West Africa. Two studies in Egypt (Khater & Mousa 2012; Sabbour et al 2018) examined older adults in nursing homes. The four studies (K. Gray et al 2017; Lewis et al 2018b, 2018a, 2019) in Tanzania and one study in Egypt (Ebeid et al 2016) examined older adults in a community setting, and the other four studies comprised older adults from hospitals. Except for three studies conducted in Nigeria (Adebusoye et al 2019), Tanzania (K. Gray et al 2017) and Egypt (Khater & Mousa 2012) that had a prospective cohort study design, the studies used a cross-sectional study design. Studies conducted in Tunisia (Hammami et al 2020) and Tanzania (K. Gray et al 2017) included adults aged >65 and >70 years, respectively, and the remaining studies included adults aged ≥60 years. As depicted in Table 1, four thousand one hundred twelve participants were included, and the sample sizes for each study ranged from 84 (Khater & Mousa 2012) to 1207 (Lewis et al 2018a).

The Brief Frailty Instrument for Tanzania, Comprehensive Geriatric Assessment, Canadian Study of Health and Aging Scale, Fried frailty criteria, Osteoporotic Fractures Frailty Index, Modified Short Emergency Geriatric Assessment Score, Study of Osteoporotic Fractures, and Survey of the Health, Aging, and Retirement in Europe were used to measure the outcomes variable across the studies.

The included articles were confirmed to be eligible for analysis and demonstrated quality scores between 72.7% and 100% (Table 1). Some studies did not explicitly address strategies to manage (Adebusoye et al 2019; Hammami et al 2020; K. Gray et al 2017; Sabbour et al 2018) or identify (Adebusoye et al 2019; Ajayi et al 2021; Hammami et al 2020; Sabbour et al 2018) confounding factors. The detailed quality appraisal evaluations and results for the cross-sectional and cohort studies are provided in Appendix 2 and Appendix 3, respectively, as supplementary data.

**Pooled prevalence of frailty**

In this systematic review and meta-analysis, a forest plot was used to estimate the pooled effect size and to examine the effect of each study with its respective confidence interval (CI) to provide a visual summary of the data. The forest plot indicated that the pooled prevalence of frailty among older adults in Africa was 38.64% (95% CI: 26.11, 51.17; Figure 2).

**Subgroup analysis**

Based on the presence of comorbidities, the region/country and setting where the studies were conducted, we conducted subgroup analyses to assess possible sources of heterogeneity. The results indicated the highest prevalence of frailty among older adults in West Africa (57.23%; 95% CI: 45.47, 68.99), followed by North Africa (43.44%; 95% CI: 22.34, 64.55; Appendix 4). In terms of setting, the highest prevalence of frailty was found in hospitals (53.04%; 95% CI: 38.28, 67.80), followed by nursing homes (47.96%; 95% CI: −8.97, 104.90; Appendix 5), and individuals with comorbidities had a higher prevalence of frailty than those without comorbidities (41.81%; 95% CI: 24.65, 58.96; Appendix 6). All subgroup analysis results are depicted in Table 2.

**Risk factors for frailty**

Of the included studies, three (Lewis et al. 2018; Lewis et al 2019; Sabbour et al 2018) identified risk factors using simple descriptive and chi-square statistics, two studies (Ebeid et al 2016; Gray et al. 2017) used correlational analysis to determine risk factors for frailty, and two studies (Hammami et al 2020; Khater & Mousa 2012) not did report risk factors for frailty. Although many risk factors were identified in some of the primary studies included in this systematic review and meta-analysis, as illustrated in Table 1, we identified the presence of comorbidities (AOR = 1.6, 95% CI: 1.77–2.18) as a predictor of or risk factor for frailty among older adults (Table 3).

**Discussion**

Studies revealed that Africans have a higher risk of frailty than people of European descent do (Papathanasiou et al. 2021). However, research on frailty in Africa is fragmented. Hence, using the available evidence, this systematic review and meta-analysis estimated the overall prevalence of frailty among older adults in Africa.

According to the results, the lowest and highest reported prevalence of frailty were 19% (Khater & Mousa 2012) and 77.1% (Sabbour et al 2018), respectively. The causes of the varied frailty prevalence across the studies might be disparities in study settings, characteristics of the study population, and presence or absence of comorbidities. Additionally, the included studies used multiple measurement tools to determine the frailty status of older adults. Our study revealed that the pooled prevalence of frailty among older adults in Africa was 38.64% (95% CI: 26.11, 51.17).

Compared with our study, studies in various countries revealed a higher prevalence of frailty among older adults. Studies in Indonesia (Rizka et al. 2021), China (Liu et al. 2020), the United States of America (Pandey et al. 2019), Taiwan (Weng et al. 2021), Greece (Papathanasiou et al. 2021), and Poland (Bąk et al. 2021) determined prevalence rates of 43.2%, 55.6%, 50%, 74.9%, 54.1% and 43.2%, respectively, for older adults. Studies in Spain also revealed that 68.8% (González-Vaca et al. 2014), 69.3% (De La Rica-Escuín et al. 2014), and 89.2% (Vidán et al. 2016) of older adults were frail. The discrepancy between the results of the current systematic review and meta-analysis and those of comparable studies might be due to differences in the inclusion criteria, study setting, or clinical characteristics of the study participants. The studies in Spain included senior older adults living in nursing homes (González-Vaca et al. 2014; De La Rica-Escuín et al. 2014) who were admitted to hospitals with a diagnosis of heart failure that was associated with other comorbidities (Vidán et al. 2016). The studies in Indonesia (Rizka et al. 2021) and China (Liu et al. 2020) included older adults from nursing homes, and the study in the United States recruited older hospitalized patients with acute decompensated heart failure (Pandey et al. 2019). The study in Greece (Papathanasiou et al. 2021) recruited older adults from care centers, and the studies in Taiwan (Weng et al. 2021) and Poland (Bąk et al. 2021) included older adults with diagnoses of chronic heart failure and diabetes mellitus, respectively, with various comorbidities. Additionally, a systematic review and meta-analysis revealed that older adults living in nursing homes had higher rates of frailty, in which the pooled prevalence of frailty was 52.3% (Kojima 2015). Older age and admittance to a healthcare facility because of various medical problems and the combined effects of multiple physical, metabolic, and psychological problems led to higher rates of frailty in older adults. However, compared with studies conducted in other countries, this pooled analysis of studies of frailty among older adults in Africa determined higher rates of frailty. For example, studies on older adults in Thailand (Wanaratna et al. 2019),(Wongtrakulruang et al. 2020), Japan (Tanikawa et al. 2019), Vietnam (Vu et al. 2017), and Spain (Otones Reyes et al. 2020) determined frailty prevalence rates of 9.4%, 15.9%, 21.4%, 31.9%, and 20.1%, respectively. Studies in China (Liu et al. 2021; Ma et al. 2018, 2020) also demonstrated lower rates of frailty among older adults, with prevalence rates of 19.6%, 16.5%, and 13.8%. Furthermore, systematic reviews conducted in the Netherlands, China, Europe, and Japan revealed that the overall prevalence of frailty in older adults was lower than our finding (Collard et al. 2012; Kojima et al. 2017; Verlaan et al. 2017; Xu et al. 2021), with rates of 10.7%, 18%, 19.1%, and 7.4%, respectively. This variation between our result and the reported lower prevalence of frailty across multiple studies might be due to several reasons. The low frailty status in these studies might be because the studies in Thailand recruited healthy older adults who visited outpatient clinics, used multiple frailty assessment tools (Wongtrakulruang et al. 2020), and included older adults with specific disease diagnoses but no comorbidities (Wanaratna et al. 2019). A study in Spain also recruited a small sample of older adults with a single diagnosis (Otones Reyes et al. 2020). Additionally, the study conducted in Japan applied strict exclusion criteria for disabilities, acute illnesses, and unstable chronic diseases (Tanikawa et al. 2019). The study in Vietnam recruited older adults from geriatric hospitals where patients received age-specific and organized care. The study in Vietnam excluded older adults with severe illnesses and included older adults with no known comorbidities (Vu et al. 2017), which might have resulted in a lower prevalence of frailty. Most older adults in our studies, however, might not receive the age-friendly care that older adults from high, middle, and lower-middle income countries often do. Many African countries lack a well-trained healthcare provider and healthcare facility specifically designed to provide age-friendly care to older adults. Moreover, older adults in Africa are prone to various biopsychosocial-related problems that might cause frailty.

The subgroup analysis in our study revealed that the highest prevalence of frailty occurred in West Africa. Compared with those in the other regions, the studies in West Africa (Ajayi et al 2021),(Adebusoye et al 2019) recruited relatively large samples of older adults with various comorbidities from institutions. Our study also revealed that older adults recruited from hospitals and nursing homes exhibited higher frailty levels than older adults recruited from communities. Adults recruited from these institutional settings had various chronic medical conditions that affected their daily lives and led them to become frail. Although several studies on frailty in Southern Africa (Barker et al. 2021; Edwards et al 2020; Leopold-George 2019) were identified, no study met the preset eligibility criteria.The few existing studies indicated various prevalence rates of frailty in Africa. These variations might be caused by several factors, including living with comorbidities, hospitalization, or setting and lifestyle differences across the African continent. A factor contributing to the variation might be the studies’ use of multiple outcome measurement tools, most of which measured the physical health of older adults. Frailty constitutes precise measurements of the symptoms of aging, which encompass the loss of physical, social, and cognitive abilities (Hammami et al. 2020; Jiao et al. 2020). Hence, tools that can address the multidimensional nature of frailty shall be used to measure the condition.

**Strength and limitation**

This is the first systematic review and meta-analysis to measure the extent of frailty among older adults in Africa. We conducted an inclusive literature search of nine electronic databases using a comprehensive search strategy to capture regionally published studies. No date limit was set in the search of the available literature. Our study was limited by the lack of representative studies from each region of Africa. No study from Southern Africa that met our eligibility criteria. This might present gaps in the estimations of the overall burden of frailty on the African continent. Hence, the finding should be interpreted with caution.

**Conclusion**

Although frailty of older adults has not yet received the attention of many academic studies, clinical services, or healthcare agendas, this systematic review and meta-analysis revealed that frailty among older adults in Africa is highly prevalent. Older adults who were hospitalized, living in nursing homes, and living with various chronic diseases exhibited a higher prevalence of frailty. This study will be a steppingstone for researchers, health professionals, concerned governmental agencies like ministry of health of each country in Africa, WHO, and policymakers in Africa to recognize this crucial health concern. Finally, comprehensive, and inclusive additional research on prevalence and its associated factors of frailty among older adults in each African region is highly imperative.

**List of abbreviations**

AOR: Adjusted odds ratio CI: Confidence interval

JBI: Joanna Briggs Institute

NIHR: National Institute for Health Research UN: United Nations

USA: United States of America WHO: World Health Organization

**Declarations**

**Ethics approval and consent to participate**: Not applicable

**Consent for publication:** Not applicable

**Availability of data and materials:** All data generated or analysed during this study are included in this published article

**Conflict of interests:** The authors declare that they have no competing interests.

**Funding**: No fund was received.

**Acknowledgement**

We would like to extend our acknowledgment to the Wallace Academic Editing for their editing service of this manuscript.

**References**

Adebusoye et al. 2019. “Frailty and Mortality among Older Patients in a Tertiary Hospital in Nigeria.” *Ghana Medical Journal* 53(3):210–16. doi: 10.4314/gmj.v53i3.5.

African Union. 2020. “Aljazeera Africa ’ s Population Is Aging and It Needs Social Protection.” 1–2.

Ajayi et al. 2021. “Prevalence and Correlates of Frailty Syndrome among Older Adults Attending Chief Tony Anenih Geriatric Centre, University College Hospital, Ibadan.” *West African Journal of Medicine* 38(3):255–67.

Aromataris, E., Z. Munn, S. Moola, C. Tufanaru, K. Sears, R. Sfetcu, M. Currie, R. Qureshi, P. Mattis, K. Lisy, and P. F. Mu. 2020. “The Joanna Briggs Institute Critical Appraisal Tools for Use in JBI Systematic Reviews: Checklist for Analytical Cross Sectional Studies.” *Joanna Briggs Institute Reviewer’s Manual* Capítulo 7.

Ávila-Funes, José Alberto, Hélène Amieva, Pascale Barberger-Gateau, Mélanie Le Goff, Nadine Raoux, Karen Ritchie, Isabelle Carrière, Béatrice Tavernier, Christophe Tzourio, Luis Miguel Gutiérrez-Robledo, and Jean François Dartigues. 2009. “Cognitive Impairment Improves the Predictive Validity of the Phenotype of Frailty for Adverse Health Outcomes: The Three-City Study.” *Journal of the American Geriatrics Society* 57(3):453–61. doi: 10.1111/j.1532-5415.2008.02136.x.

Bąk, E., A. Młynarska, C. Marcisz, R. Bobiński, D. Sternal, and R. Młynarski. 2021. “The Influence of Frailty Syndrome on Quality of Life in Elderly Patients with Type 2 Diabetes.” *Quality of Life Research* 30(9):2487–95. doi: 10.1007/s11136-021-02829-x.

Barker, Fred J., Justine I. Davies, F. Xavier Gomez-olive, Kathleen Kahn, Fiona E. Matthews, Collin F. Payne, Joshua A. Salomon, Stephen M. Tollman, Alisha N. Wade, Richard W. Walker, and Miles D. Witham. 2021. “Developing and Evaluating a Frailty Index for Older South Africans — Findings from the HAALSI Study.” *Age and Ageing* 1–7. doi: 10.1093/ageing/afab111.

Chen CY, Wu SC, Chen LJ, et al. 2010. “The Prevalence of Subjective Frailty and Factors Associated with Frailty in Taiwan.” *Arch Gerontol Geriatr* 50:543–57.

Collard, Rose M., Han Boter, Robert A. Schoevers, and Richard C. Oude Voshaar. 2012. “Prevalence of Frailty in Community-Dwelling Older Persons: A Systematic Review.” *Journal of the American Geriatrics Society* 60(8):1487–92. doi: 10.1111/j.1532- 5415.2012.04054.x.

Duzgun, Gonul, Sema Ustundag, and Ayfer Karadakovan. 2021. “Assessment of Frailty in the Elderly.” *Florence Nightingale Journal of Nursing* 29(1):2–8. doi: 10.5152/fnjn.2021.414736.

Ebeid et al. 2016. “Frailty Prevalence and Correlates among Free Living Elderly in an Egyptian Rural Elderly.” *International Journal Of Nternational Journal Of Recent Scientific Recent Scientific Research* 7(2):1–8.

Edwards et al. 2020. “HIV Serostatus, Inflammatory Biomarkers and the Frailty Phenotype among Older People in Rural KwaZulu-Natal, South Africa.” *Afr J AIDS Res.* 19(3):177–85.

Espinoza, Sara E., Inkyung Jung, and Helen Hazuda. 2012. “Frailty Transitions in the San Antonio Longitudinal Study of Aging.” *Journal of the American Geriatrics Society* 60(4):652–60. doi: 10.1111/j.1532-5415.2011.03882.x.

Fried, L. P., C. M. Tangen, J. Walston, A. B. Newman, C. Hirsch, J. Gottdiener, T. Seeman, and

R. Tracy. 2001. “Frailty in Older Adults : Evidence for a Phenotype.” 56(3). doi: 10.1093/gerona/56.3.m146.

González-Vaca, Julia, Marisa De La Rica-Escuín, Marta Silva-Iglesias, María Dolores Arjonilla- García, Rosana Varela-Pérez, José Luis Oliver-Carbonell, and Pedro Abizanda. 2014. “Frailty in Institutionalized Older Adults from Albacete. The FINAL Study: Rationale, Design, Methodology, Prevalence and Attributes.” *Maturitas* 77(1):78–84. doi: 10.1016/j.maturitas.2013.10.005.

Gray, William K., Golda Orega, Aloyce Kisoli, Jane Rogathi, Stella Maria Paddick, Anna R. Longdon, Richard W. Walker, Felicity Dewhurst, Matthew Dewhurst, Paul Chaote, and Catherine Dotchin. 2017. “Identifying Frailty and Its Outcomes in Older People in Rural Tanzania.” *Experimental Aging Research* 43(3):257–73. doi: 10.1080/0361073X.2017.1298957.

Graziano Onder, Davide L, Alessandra et al. 2018. “Accounting for Frailty When Treating Chronic Diseases.” *European Journal of Internal Medicine* 56(2):49–52.

Hammami et al. 2020. “Evaluation of Pro-Inflammatory Cytokines in Frail Tunisian Older Adults.” *PLoS ONE* 15(11 November):1–13. doi: 10.1371/journal.pone.0242152.

Hammami, Sonia, Amira Zarrouk, Cecile Piron, Ioana Almas, Nabil Sakly, and Veronique Latteur. 2020. “Prevalence and Factors Associated with Frailty in Hospitalized Older Patients.” *BMC Geriatrics* 20(1):1–9. doi: 10.1186/s12877-020-01545-4.

Higgins, Julian P. T., and Simon G. Thompson. 2002. “Quantifying Heterogeneity in a Meta- Analysis.” *Statistics in Medicine* 21(11):1539–58. doi: 10.1002/sim.1186.

Huedo-Medina, Tania B., Julio Sánchez-Meca, Fulgencio Marín-Martínez, and Juan Botella.

2006. “Assessing Heterogeneity in Meta-Analysis: Q Statistic or I 2 Index?” *Psychological Methods* 11(2):193–206. doi: 10.1037/1082-989X.11.2.193.

JBI. 2016. “The Joanna Briggs Institute Critical Appraisal Tools for Use in JBI Systematic Reviews: Checklist for Cohort Studies.” *Joanna Briggs Institute Reviewer’s Manual* 1–7.

Jiao, Jing, Yu Wang, Chen Zhu, Fangfang Li, Minglei Zhu, Xianxiu Wen, Jingfen Jin, Hui Wang, Dongmei Lv, Shengxiu Zhao, Xinjuan Wu, and Tao Xu. 2020. “Prevalence and Associated Factors for Frailty among Elder Patients in China: A Multicentre Cross-Sectional Study.” *BMC Geriatrics* 20(1):1–10. doi: 10.1186/s12877-020-1496-1.

K. Gray et al. 2017. “Identifying Frailty and Its Outcomes in Older People in Rural Tanzania.”

*Experimental Aging Research* 43(3):257–73. doi: 10.1080/0361073X.2017.1298957.

Kamiya, Yumiko. 2016. “Overview of Demographic Ageing in Africa.” (July):1–13.

Khater & Mousa. 2012. “Predicting Falls among Egyptian Nursing Home Residents: A 1-Year Longitudinal Study.” *Journal of Clinical Gerontology and Geriatrics* 3(2):73–76. doi: 10.1016/j.jcgg.2012.04.005.

Kojima, Gotaro. 2015. “Prevalence of Frailty in Nursing Homes: A Systematic Review and Meta- Analysis.” *Journal of the American Medical Directors Association* 16(11):940–45. doi: 10.1016/j.jamda.2015.06.025.

Kojima, Gotaro, Steve Iliffe, Yu Taniguchi, Hiroyuki Shimada, Hiromi Rakugi, and Kate Walters. 2017. “Prevalence of Frailty in Japan: A Systematic Review and Meta-Analysis.” *Journal of Epidemiology* 27(8):347–53. doi: 10.1016/j.je.2016.09.008.

De La Rica-Escuín, Marisa, Julia González-Vaca, Rosana Varela-Pérez, María Dolores Arjonilla- García, Marta Silva-Iglesias, José Luis Oliver-Carbonell, and Pedro Abizanda. 2014. “Frailty and Mortality or Incident Disability in Institutionalized Older Adults: The FINAL Study.” *Maturitas* 78(4):329–34. doi: 10.1016/j.maturitas.2014.05.022.

Leopold-George, G. D. Nethathe. 2019. “Frailty in Perioperative Patients in Three South African Academic Hospitals.” *S Afr Med J* 109(7):535–40.

Lewis, Emma Grace, Selina Coles, Kate Howorth, John Kissima, William Gray, Sarah Urasa, Richard Walker, and Catherine Dotchin. 2018. “The Prevalence and Characteristics of Frailty by Frailty Phenotype in Rural Tanzania.” *BMC Geriatrics* 18(1):1–11. doi: 10.1186/s12877-018-0967-0.

Lewis et al. 2018a. “Prevalence of Frailty in Older Community-Dwelling Tanzanians According to Comprehensive Geriatric Assessment.” *Journal of the American Geriatrics Society* 66(8):1484–90. doi: 10.1111/jgs.15433.

Lewis et al. 2018b. “The Prevalence and Characteristics of Frailty by Frailty Phenotype in Rural Tanzania.” *BMC Geriatrics* 18(283):1–11.

Lewis et al. 2019. “A Brief Frailty Screening Tool in Tanzania: External Validation and Refinement of the B-FIT Screen.” *Aging Clinical and Experimental Research* 32(10):1959– 67. doi: 10.1007/s40520-019-01406-0.

Lin, Lifeng, and Haitao Chu. 2018a. “Quantifying Publication Bias in Meta-Analysis.” *Biometrics*

74(3):785–94. doi: 10.1111/biom.12817.

Lin, Lifeng, and Haitao Chu. 2018b. “Quantifying Publication Bias in Meta-Analysis.”

*Biometrics* 74:785–94. doi: 10.1111/biom.12817.

Liu, Pan, Yaxin Zhang, Yun Li, Shijie Li, Ying Li, Yumeng Chen, Ou Zhao, Yu Song, Bixi Li, Tong Ji, Yiming Pan, and Lina Ma. 2021. “Association of Frailty with Quality of Life in Older Hypertensive Adults: A Cross-Sectional Study.” *Quality of Life Research* 30(8):2245–53. doi: 10.1007/s11136-021-02816-2.

Liu, Weiwei, Martine Puts, Fen Jiang, Chuyi Zhou, Siyuan Tang, and Sanmei Chen. 2020. “Physical Frailty and Its Associated Factors among Elderly Nursing Home Residents in China.” *BMC Geriatrics* 20(1):1–10. doi: 10.1186/s12877-020-01695-5.

Luciana Correia Alves, Yeda Aparecida de Oliveira Duarte, Jair Licio Ferreira Santos. 2018. “Factors Associated the Transitions in the Frailty States Among Elderly in Brazil 2006- 2010.” 1–16.

Ma, Lina, Jagadish K. Chhetri, Pan Liu, Tong Ji, Li Zhang, and Zhe Tang. 2020. “Epidemiological Characteristics and Related Factors of Frailty in Older Chinese Adults with Hypertension: A Population-Based Study.” *Journal of Hypertension* 38(11):2192–97. doi: 10.1097/HJH.0000000000002650.

Ma, Lina, Li Zhang, Fei Sun, Yun Li, and Zhe Tang. 2018. “Frailty in Chinese Older Adults with Hypertension: Prevalence, Associated Factors, and Prediction for Long-Term Mortality.” *Journal of Clinical Hypertension* 20(11):1595–1602. doi: 10.1111/jch.13405.

Maresova, Petra, Ehsan Javanmardi, Sabina Barakovic, Jasmina Barakovic Husic, Signe Tomsone, Ondrej Krejcar, and Kamil Kuca. 2019. “Consequences of Chronic Diseases and Other Limitations Associated with Old Age - A Scoping Review.” *BMC Public Health* 19(1). doi: 10.1186/s12889-019-7762-5.

Moher, David, Alessandro Liberati, Jennifer Tetzlaff, and Douglas G. Altman. 2009. “Preferred Reporting Items for Systematic Reviews and Meta-Analyses: The PRISMA Statement.” *BMJ (Online)* 339(7716):332–36. doi: 10.1136/bmj.b2535.

Mulualem, Getaneh, Amare Wondim, and Abere Woretaw. 2019. “The Effect of Pregnancy Induced Hypertension and Multiple Pregnancies on Preterm Birth in Ethiopia: A Systematic Review and Meta-Analysis.” *BMC Research Notes* 12(1):91. doi: 10.1186/s13104-019-4128- 0.

Naeem et al. 2020. “Prevalence of Frailty among Elderly Patients Attending Primary Health Care Centers in Sixth of October City.” *The Egyptian Journal of Geriatrics and Gerontology* 7(2):5–11. doi: 10.21608/ejgg.2020.139253.

Nguyen, T. N., R. G. Cumming, and S. N. Hilmer. 2015. “A Review of Frailty in Developing Countries.” *The Journal of Nutrition, Health & Aging* 19(9):941–46. doi: 10.1007/s12603- 015-0503-2.

O’Caoimh, Rónán, Duygu Sezgin, Mark R. O’Donovan, D. William Molloy, Andrew Clegg, Kenneth Rockwood, and Aaron Liew. 2021. “Prevalence of Frailty in 62 Countries across the World: A Systematic Review and Meta-Analysis of Population-Level Studies.” *Age and Ageing* 50(1):96–104. doi: 10.1093/ageing/afaa219.

Otones Reyes, Pedro, Eva García Perea, Milagros Rico Blázquez, and Azucena Pedraz Marcos.

2020. “Prevalence and Correlates of Frailty in Community-Dwelling Older Adults with Chronic Pain: A Cross-Sectional Study.” *Pain Management Nursing* 21(6):530–35. doi: 10.1016/j.pmn.2020.05.009.

Pandey, Ambarish, Dalane Kitzman, David J. Whellan, Pamela W. Duncan, Robert J. Mentz, Amy M. Pastva, M. Benjamin Nelson, Bharathi Upadhya, Haiying Chen, and Gordon R. Reeves. 2019. “Frailty Among Older Decompensated Heart Failure Patients: Prevalence, Association With Patient-Centered Outcomes, and Efficient Detection Methods.” *JACC: Heart Failure* 7(12):1079–88. doi: 10.1016/j.jchf.2019.10.003.

Papathanasiou, Ioanna V, Anna Rammogianni, Dimitrios Papagiannis, Foteini Malli, Dimitrios C. Mantzaris, Konstantinos Tsaras, Lamprini Kontopoulou, Evridiki Kaba, Martha Kelesi, and Evangelos C. Fradelos. 2021. “Frailty and Quality of Life Among Community-Dwelling Older Adults.” *Cureus* 13(2):1–9. doi: 10.7759/cureus.13049.

Ribeiro, Ingrid Alves, Luciano Ramos de Lima, Cris Renata Grou Volpe, Silvana Schwerz Funghetto, Tânia Cristina Maria Santa Barbara Rehem, and Marina Morato Stival. 2019. “Frailty Syndrome in the Elderly in Elderly with Chronic Diseases in Primary Care.” *Revista Da Escola de Enfermagem* 53:1–9. doi: 10.1590/S1980-220X2018002603449.

Rizka, Aulia, Andika Indrarespati, Noto Dwimartutie, and Muhadi Muhadi. 2021. “Frailty among Older Adults Living in Nursing Homes in Indonesia: Prevalence and Associated Factors.” *Annals of Geriatric Medicine and Research* 25(2):93–97. doi: 10.4235/agmr.21.0033.

Rücker, Gerta, Guido Schwarzer, James R. Carpenter, and Martin Schumacher. 2008. “Undue Reliance on I 2 in Assessing Heterogeneity May Mislead.” *BMC Med Res Methodol* 8(79). doi: 10.1186/1471-2288-8-79.

Sabbour et al. 2018. “Frailty and Malnutrition among Egyptian Elderly: Prevalence and Risk Factors in Nursing Home and Community Dwelling Elderly.” *The Egyptian Journal of Geriatrics and Gerontology* 5(2):1–5. doi: 10.21608/ejgg.2018.30909.

Tanikawa, Takahisa, Sayuri Sable-Morita, Haruhiko Tokuda, and Hidenori Arai. 2019. “Frailty Prevalence and Characteristics in Older Patients with Type 2 Diabetes.” *Journal of Diabetes Mellitus* 09(02):31–38. doi: 10.4236/jdm.2019.92004.

Uchmanowicz, Izabella, Anna Chudiak, Beata Jankowska-pola, and Robbert Gobbens. 2017. “Hypertension and Frailty Syndrome in Old Age : Current Perspectives.” *Cardiac Failure Review* 3(2):102–7. doi: 10.15420/cfr.2017.

Verlaan, Sjors, Gerdien C. Ligthart-Melis, Sander L. J. Wijers, Tommy Cederholm, Andrea B. Maier, and Marian A. E. de van der Schueren. 2017. “High Prevalence of Physical Frailty Among Community-Dwelling Malnourished Older Adults–A Systematic Review and Meta- Analysis.” *Journal of the American Medical Directors Association* 18(5):374–82. doi: 10.1016/j.jamda.2016.12.074.

Vidán, María T., Vendula Blaya-Novakova, Elísabet Sánchez, Javier Ortiz, José A. Serra-Rexach, and Héctor Bueno. 2016. “Prevalence and Prognostic Impact of Frailty and Its Components in Non-Dependent Elderly Patients with Heart Failure.” *European Journal of Heart Failure* 18(7):869–75. doi: 10.1002/ejhf.518.

Vu, Huyen Thi Thanh, Thanh Xuan Nguyen, Tu N. Nguyen, Anh Trung Nguyen, Robert Cumming, Sarah Hilmer, and Thang Pham. 2017. “Prevalence of Frailty and Its Associated Factors in Older Hospitalised Patients in Vietnam.” *BMC Geriatrics* 17(1):1–7. doi: 10.1186/s12877-017-0609-y.

Wanaratna, Kulthanit, Weerasak Muangpaisan, Vilai Kuptniratsaikul, Chalobol Chalermsri, and Apiwan Nuttamonwarakul. 2019. “Prevalence and Factors Associated with Frailty and Cognitive Frailty Among Community-Dwelling Elderly with Knee Osteoarthritis.” *Journal of Community Health* 44(3):587–95. doi: 10.1007/s10900-018-00614-5.

Weng, Shuo Chun, Chu Sheng Lin, Der Cherng Tarng, and Shih Yi Lin. 2021. “Physical Frailty

and Long-Term Mortality in Older People with Chronic Heart Failure with Preserved and Reduced Ejection Fraction: A Retrospective Longitudinal Study.” *BMC Geriatrics* 21(1):1– 12. doi: 10.1186/s12877-020-01971-4.

Wongtrakulruang, Panuwat, Weerasak Muangpaisan, Bubpha Panpradup, Aree Tawatwattananun, Monchai Siribamrungwong, and Sasinapha Tomongkon. 2020. “The Prevalence of Cognitive Frailty and Pre-Frailty among Older People in Bangkok Metropolitan Area: A Multicenter Study of Hospital-Based Outpatient Clinics.” *Journal of Frailty, Sarcopenia and Falls* 05(03):62–71. doi: 10.22540/jfsf-05-062.

Xu, Rui, Qiufang Li, Feifei Guo, Maoni Zhao, and Luyao Zhang. 2021. “Prevalence and Risk Factors of Frailty among People in Rural Areas: A Systematic Review and Meta-Analysis.” *BMJ Open* 11(4):1–8. doi: 10.1136/bmjopen-2020-043494.

**Appendix 1: PRISMA 2020 Checklist**

| **Section and Topic** | | **Item #** | | **Checklist item** | | **Location where item is reported** | |
| --- | --- | --- | --- | --- | --- | --- | --- |
| **TITLE** | | | | | |  | |
| Title | | 1 | | Identify the report as a systematic review. | | Page 1 | |
| **ABSTRACT** | | | | | |  | |
| Abstract | | 2 | | See the PRISMA 2020 for Abstracts checklist. | | Page 2 | |
| **INTRODUCTION** | | | | | |  | |
| Rationale | | 3 | | Describe the rationale for the review in the context of existing knowledge. | | Page 4 | |
| Objectives | | 4 | | Provide an explicit statement of the objective(s) or question(s) the review addresses. | | Page 4 | |
| **METHODS** | | | | | |  | |
| Eligibility criteria | | 5 | | Specify the inclusion and exclusion criteria for the review and how studies were grouped for the syntheses. | | Page 5 | |
| Information sources | | 6 | | Specify all databases, registers, websites, organisations, reference lists and other sources searched or consulted to identify studies. Specify the date when each source was last searched or consulted. | | Page 5 | |
| Search strategy | | 7 | | Present the full search strategies for all databases, registers and websites, including any filters and limits used. | | Page 5 | |
| Selection process | | 8 | | Specify the methods used to decide whether a study met the inclusion criteria of the review, including how many reviewers screened each record and each report retrieved, whether they worked independently, and if applicable, details of automation tools used in the process. | | Page 5 | |
| Data collection process | | 9 | | Specify the methods used to collect data from reports, including how many reviewers collected data from each report, whether they worked independently, any processes for obtaining or confirming data from study investigators, and if applicable, details of automation tools used in the process. | | Page 5 | |
| Data items | | 10a | | List and define all outcomes for which data were sought. Specify  whether all results that were compatible with each outcome domain in | | Page 6 | |
| **Section and Topic** | **Item #** | | **Checklist item** | | **Location where item is reported** | |  |
|  |  | | each study were sought (e.g. for all measures, time points, analyses), and if not, the methods used to decide which results to collect. | |  | |  |
|  | 10b | | List and define all other variables for which data were sought (e.g. participant and intervention characteristics, funding sources). Describe any assumptions made about any missing or unclear information. | | Page 6 | |  |
| Study risk of bias assessment | 11 | | Specify the methods used to assess risk of bias in the included studies, including details of the tool(s) used, how many reviewers assessed each study and whether they worked independently, and if applicable, details of automation tools used in the process. | | Page 6 | |  |
| Effect measures | 12 | | Specify for each outcome the effect measure(s) (e.g. risk ratio, mean difference) used in the synthesis or presentation of results. | | Page 7 | |  |
| Synthesis methods | 13a | | Describe the processes used to decide which studies were eligible for each synthesis (e.g. tabulating the study intervention characteristics and comparing against the planned groups for each synthesis (item #5)). | | Page 7 | |  |
|  | 13b | | Describe any methods required to prepare the data for presentation or synthesis, such as handling of missing summary statistics, or data conversions. | | Page 7 | |  |
|  | 13c | | Describe any methods used to tabulate or visually display results of individual studies and syntheses. | | Page 7 | |  |
|  | 13d | | Describe any methods used to synthesize results and provide a rationale for the choice(s). If meta-analysis was performed, describe the model(s), method(s) to identify the presence and extent of statistical heterogeneity, and software package(s) used. | | Page 7 | |  |
|  | 13e | | Describe any methods used to explore possible causes of heterogeneity among study results (e.g. subgroup analysis, meta-regression). | | Page 7 | |  |
|  | 13f | | Describe any sensitivity analyses conducted to assess robustness of the synthesized results. | | Page 7 | |  |
| Reporting bias assessment | 14 | | Describe any methods used to assess risk of bias due to missing results in a synthesis (arising from reporting biases). | | Page 7 | |  |
| **Section and Topic** | | **Item #** | | **Checklist item** | | **Location where item is reported** | |
| Certainty assessment | | 15 | | Describe any methods used to assess certainty (or confidence) in the  body of evidence for an outcome. | | Page 7 | |
| **RESULTS** | | | | | |  | |
| Study selection | | 16a | | Describe the results of the search and selection process, from the number of records identified in the search to the number of studies included in the review, ideally using a flow diagram. | | Page 7 | |
|  |  | 16b | | Cite studies that might appear to meet the inclusion criteria, but which were excluded, and explain why they were excluded. | | Page 10 | |
| Study characteristics | | 17 | | Cite each included study and present its characteristics. | | Page 7-8 | |
| Risk of bias in studies | | 18 | | Present assessments of risk of bias for each included study. | | Page 6 | |
| Results of individual studies | | 19 | | For all outcomes, present, for each study: (a) summary statistics for each group (where appropriate) and (b) an effect estimate and its precision (e.g. confidence/credible interval), ideally using structured tables or plots. | | Page 8 | |
| Results of syntheses | | 20a | | For each synthesis, briefly summarise the characteristics and risk of bias among contributing studies. | | Page 8 | |
|  |  | 20b | | Present results of all statistical syntheses conducted. If meta-analysis was done, present for each the summary estimate and its precision (e.g. confidence/credible interval) and measures of statistical heterogeneity. If comparing groups, describe the direction of the effect. | | Page 8 | |
|  |  | 20c | | Present results of all investigations of possible causes of heterogeneity among study results. | | Page 8 | |
|  |  | 20d | | Present results of all sensitivity analyses conducted to assess the robustness of the synthesized results. | | Page 8 | |
| Reporting biases | | 21 | | Present assessments of risk of bias due to missing results (arising from reporting biases) for each synthesis assessed. | | Page 8 | |
| Certainty of evidence | | 22 | | Present assessments of certainty (or confidence) in the body of evidence for each outcome assessed. | | Page 8 | |

| **Section and Topic** | **Item #** | **Checklist item** | **Location where item is reported** |
| --- | --- | --- | --- |
| **DISCUSSION** | | |  |
| Discussion | 23a | Provide a general interpretation of the results in the context of other evidence. | Page 9 |
|  | 23b | Discuss any limitations of the evidence included in the review. | Page 11 |
|  | 23c | Discuss any limitations of the review processes used. | Page 11 |
|  | 23d | Discuss implications of the results for practice, policy, and future  research. | Page 12 |
| **OTHER INFORMATION** | | |  |
| Registration and protocol | 24a | Provide registration information for the review, including register name and registration number, or state that the review was not registered. | Page 5 |
|  | 24b | Indicate where the review protocol can be accessed, or state that a protocol was not prepared. | Page 5 |
|  | 24c | Describe and explain any amendments to information provided at registration or in the protocol. | Page 5 |
| Support | 25 | Describe sources of financial or non-financial support for the review, and the role of the funders or sponsors in the review. | Page 12 |
| Competing interests | 26 | Declare any competing interests of review authors. | Page 12 |
| Availability of data, code and other materials | 27 | Report which of the following are publicly available and where they can be found: template data collection forms; data extracted from included studies; data used for all analyses; analytic code; any other materials used in the review. | Page 12 |

**Appendix 2**

**Critical appraisal result of the included studies done using cross sectional studies, 2021**

**Criterion No (items included to appraise cross-sectional**

**Included articles studies).**

|  | **1** | **2** | **3** | **4** | **5** | **6** | **7** | **8** | **%** |
| --- | --- | --- | --- | --- | --- | --- | --- | --- | --- |
| **Ajayi et al. 20219** | ✓ | ✓ | ✓ | ✓ | * | ✓ | ✓ | ✓ | 87.5 |
| **Hammami et al. 2020** | ✓ | ✓ | ✓ | ✓ | ✓ | ✓ | ✓ | ✓ | 100 |
| **Naeem et al. 2020** | ✓ | ✓ | ✓ | ✓ | ✓ | ✓ | ✓ | ✓ | 100 |
| **Lewis et al. 2019** | ✓ | ✓ | ✓ | ✓ | ✓ | ✓ | ✓ | ✓ | 100 |
| **Sabbour et al. 2018** | ✓ | ✓ | ✓ | ✓ | * | * | ✓ | ✓ | 75 |
| **Lewis et al. 2018** | ✓ | ✓ | ✓ | ✓ | ✓ | ✓ | ✓ | ✓ | 100 |
| **Lewis et al. 2018** | ✓ | ✓ | ✓ | ✓ | ✓ | ✓ | ✓ | ✓ | 100 |
| **Ebeid. et al. 2016** | ✓ | ✓ | ✓ | ✓ | * | * | ✓ | ✓ | 75 |

**√ = criterion fulfilled, X = criterion not fulfilled, * = Unclear**

Criterion No. 1: Inclusion criteria, Criterion No. 2: Description of study subject and setting, Criterion No. 3: Valid and reliable measurement of exposure, Criterion No. 4: Objective and standard criteria used, Criterion No. 5: Identification of confounder, Criterion No. 6: Strategies to handle confounder, Criterion No. 7: Outcome measurement, & Criterion No. 8: Appropriate statistical analysis.

**Appendix 3**

**Critical appraisal result of the included studies done using cohort studies, 2021**

**Criterion No (items included to appraise cohort studies).**

**Included articles**

|  | **1** | **2** | **3** | **4** | **5** | **6** | **7** | **8** | **9** | **10** | **11** | **Total** |
| --- | --- | --- | --- | --- | --- | --- | --- | --- | --- | --- | --- | --- |
| **Adebusoye et al, 2019** | ✓ | ✓ | ✓ | ***** | ***** | ✓ | ✓ | ✓ | ✓ | ¥ | ✓ | 80 |
| **K. Gray et al, 2017** | ✓ | ✓ | ✓ | ✓ | ***** | ✓ | ✓ | ✓ | X | X | ✓ | 72.7 |
| **Khater &, Mousa, 2012** | ✓ | ✓ | ✓ | ✓ | ✓ | ✓ | ✓ | ✓ | ✓ | ✓ | ✓ | 100 |

**√ = criterion fulfilled, X = criterion not fulfilled, * = Unclear, ¥ = Not applicable**

Criterion No. 1: Were the groups similar and recruited from the same population? No. 2: Eexposures measured from both exposed and unexposed groups? No. 3: Validity & reliability of exposure measurement? No. 4: Confounding factors identified? No. 5: Strategies to deal with confounding? No. 6: Participants free of the outcome at the start of the study? No. 7: Validity & reliability of outcome measurement? No. 8: Appropriate follow up time? No 9: Follow up status? No 10: Strategies to address incomplete follow- up?, No 11: Appropriate statistical analysis?


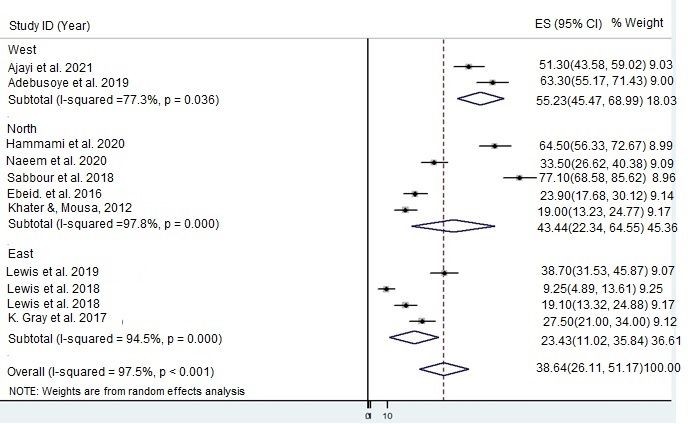
**Appendix 4:**

Random-effects pooled prevalence of frailty according to the region in which studies were undertaken, 2021

**Appendix 5**


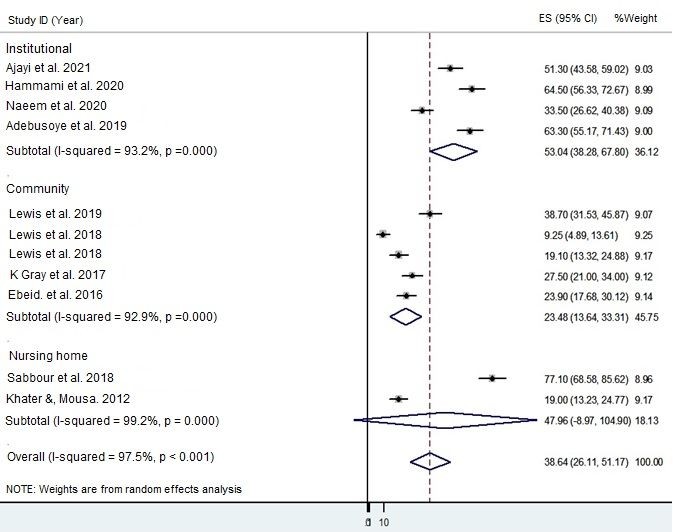


Random-effects pooled prevalence of frailty according to the setting in which studies were undertaken, 2021

For

Review

Only

**Appendix 6**

For

Review

Only


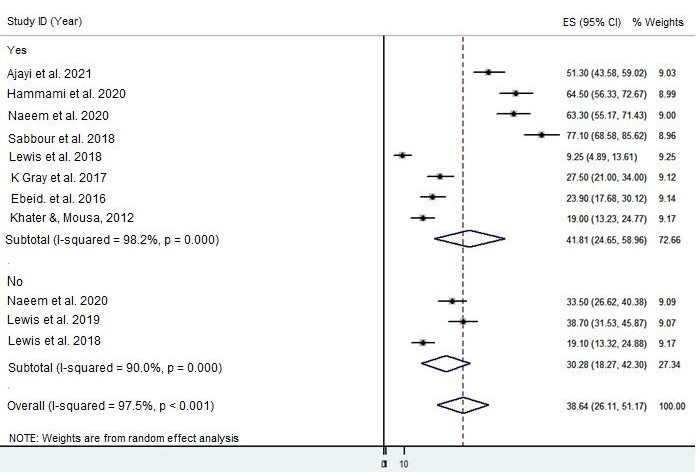


Random-effects pooled prevalence of frailty by comorbidity in the included studies, 2021

Page 32 of 37

Table 1: Characteristics of included studies assessing frailty among elderly adults in Africa, 2021.

| **Author/year** | **Country**  **/region*** | **Setting** | **Study design** | **Sampling method** | **No. of participants** | **Age** | **Mean age** | **Reported comorbidities** | **Measurement tool** | **Prevalence Reported Risk factors Quality**  **score (%)** |
| --- | --- | --- | --- | --- | --- | --- | --- | --- | --- | --- |
| Ajayi et al., 2021 | Nigeria, West | Inst. | Cross- sectional | Consecutiv e | 971 | ≥60 | 71.3 | Yes | FFC | 51.3 Increasing age, 87.5  multimorbidities, depression, increased risk of fall, and inability to perform IADL |
| Hammami et al., 2020 | Tunisia, North | Inst. | Cross- sectional | NES | 141 | ≥65 | NR | Yes | SEGAm | 64.5 NES  100 |
| Naeem et al., 2020 | Egypt, North | Inst. | Cross- sectional | NES | 230 | ≥60 | NR | No | Modified Fried | 33.5 Single leg balance test, Timed 100  Up and Go test & hand grip strength test had positive correlation with frailty |
| Adebusoye et al., 2019 | Nigeria, West | Inst. | Prospecti ve cohort | Consecutiv e | 450 | >60 | 71.5 | Yes | CSHA | 63.3 Male sex, nonengagement in 80  occupational activities, multimorbidities, functional disability, malnutrition, and being underweight |
| Lewis et al., 2019 | Tanzania  , East | Com. | Cross- sectional | Stratified sampling | 235 | ≥60 | NR | No | CGA | 38.7 physical disability, cognitive 100 |

impairment, calf circumference, poor distance vision, and problems engaging in social activities

Page 33 of 37 Nursing Open

living alone, dysfunctional family, comorbidities, receiving more than three drugs, depressive symptoms, and lower cognitive performances

| Lewis et al., 2018 | Tanzania  , East | Com. | Cross- sectional | NR | 235 | ≥60 | 74.8 | Yes | Frailty phenotype | 9.25 older age, poor self-assessed 100  health, and depression symptoms | | |
| --- | --- | --- | --- | --- | --- | --- | --- | --- | --- | --- | --- | --- |
| Lewis et al., 2018 | Tanzania  , East | Com. | Cross- sectional | Stratified | 1207 | ≥60 | NR | No | B-FIT | 19.1 older age 100 | | |
| K. Gray et al., 2017 | Tanzania  , East | Com. | Prospecti ve cohort | NES | 296 | ≥70 | NR | Yes | Frailty index | 27.5 Greater age, never 72.7  attended school, falls, dependency | | |
|  |  |  |  |  |  |  |  |  |  |  | in ADL, functional disability, and  cognitive function |  |
| Ebeid et al., 2016 | Egypt, North | Com. | Cross- sectional | NES | 88 | ≥60 | NR | Yes | SOF | 23.9 | Male sex, malnutrition, depression, low MMSE, dependent state  on ADL, worse performance of Timed Up and Go test, correlation with frailty | 75 |
| Khater & Mousa, 2012 | Egypt, North | Nursin g home | Prospecti ve cohort | NES | 84 | ≥60 | 71.9 | Yes | OFF | 19 | NR | 100 |

| Sabbour et | Egypt, | Nursin | Cross- | NR | 175 | ≥60 | NES | Yes | SHARE index | 77.1 Older age, female, widowed, 75 |
| --- | --- | --- | --- | --- | --- | --- | --- | --- | --- | --- |
| al., 2018 | North | g home | sectional |  |  |  |  |  |  |  |

B-FIT = Brief Frailty Instrument for Tanzania, CGA = Comprehensive Geriatric Assessment, Com = Community based, CSHA = Canadian Study of Health and Aging Scale, FFC = Fried Frailty Criteria, IADL = Instrumental activities of daily living, Inst. = Institution based (hospital), MMSE = Mini-Mental Status Examination, NES = Not explicitly stated, NR = Not reported, OFF = Osteoporotic fractures frailty index, SEGAm = The modified Short Emergency Geriatric Assessment score, SHARE = Survey of the Health, Aging, and Retirement in Europe, SOF = Study of Osteoporotic Fractures criteria. and *region in Africa

Table 2: Results from subgroup analyses on the prevalence of frailty among older adults in Africa, 2021.

**Variables**

**for the subgroup**

**Characteristics**

**No. of study**

**Prevalence with 95% CI *I*2 P**

| By region in | North | 5 | 43.44 (22.34, 64.55) | 97.8 | <0.001 |
| --- | --- | --- | --- | --- | --- |
| the continent | West | 2 | 57.23 (45.47, 68.99) | 77.3 | 0.036 |
|  | East | 4 | 23.43 (11.02, 35.84) | 94.5 | <0.001 |
| Setting | Institutional | 4 | 53.04 (38.28, 67.80) | 93.2 | <0.001 |
|  | Community | 4 | 23.48 (13.64, 33.31) | 92.9 | <0.001 |
|  | Nursing home | 2 | 47.96 (−8.97, 104.90) | 99.2 | <0.001 |
| Reported | Yes | 7 | 41.81 (24.65, 58.96) | 98.20 | <0.001 |

comorbidity No 3 30.28 (18.27, 42.30) 90.00 <0.001

Table 3: Pooled risk factors of frailty among elderly adults in Africa, 2021

| **No** | **Risk factors** | **AOR** | **95% CI *I*2 P** | | |
| --- | --- | --- | --- | --- | --- |
| **1** Presence of comorbidity | | 1.6 | 1.767–2.177 | 2.99 | 0.003 |
| **2** Nutritional status | | 0.407 | 0.155–1.067 | 1.83 | 0.068 |
| **3** Functional activity | | 0.573 | 0.322–1.021 | 1.89 | 0.059 |

For

Articles retrieved through combined searching

(n=317)

Articles identified through electronic database searching

(n =312)

Additional articles identified from other sources

(n=5)

**Included**

**Eligibility**

**Screening**

**Identification**

Figure 1: PRISMA flow diagram illustrating the selection of studies on frailty among older adults in Africa included in this systematic review and meta-analysis 2021.

Full text articles assessed for eligibility (n=44)

Articles screened (n=236)

Articles included in this review (n=11)

Articles excluded with reason: Outcome explicitly not reported (n=17) Not focused elderly (n= 13)

Inadequate information (n=3)

Articles excluded after reading titles (n=168) and abstracts (n=24)

Articles excluded due to duplication

(n=81)


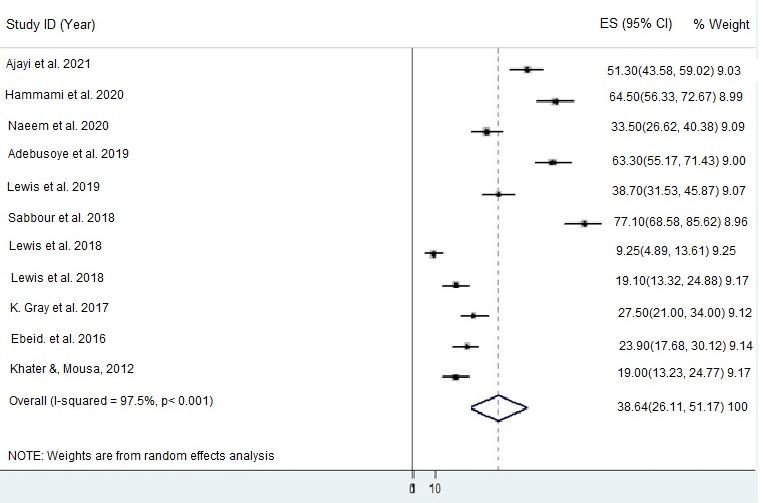


Figure 2: Prevalence of frailty among older adults in Africa, 2021

For

Review

## Appendix A2: Review two

**Title:** Depression among older adults living in resource-limited settings: A systematic review and meta-analysis

**Manuscript ID:** BMC Psych

**Depression among older adults living in resource-limited settings: A systematic review and meta-analysis**

**Ayele Semachew Kasa^1, 2, 3*^, Shu-Chun Lee (PhD) ^4^, and Hui-Chen (Rita) Chang (PhD) ^1, 2^**

**Affiliation:**

1. School of Nursing, Faculty of Science, Medicine and Health, University of Wollongong, New South Wales, Australia.
2. Illawarra Health and Medical Research Institute (IHMRI), Wollongong, New South Wales, New South Wales, Australia.
3. Department of Adult Health Nursing, School of Health Science, College of Medicine and Health Sciences, Bahir Dar University, Bahir Dar, Ethiopia.
4. School of Gerontology Health Management, Taipei Medical University, Taiwan

*Corresponding author

**Email:**

Ayele Semachew Kasa (ASK): [ask255@uowmail.edu.au](mailto:ask255@uowmail.edu.au) PO Box: 53 Porter St. North Wollongong, Australia

Shu-Chun Lee (PhD) (SCL): [sclee@tmu.edu.tw](mailto:sclee@tmu.edu.tw)

Hui Chen (Rita) Chang (PhD) (HCC): [hchang@uow.edu.au](mailto:hchang@uow.edu.au)

**Conflict of interests:** The authors declare that they have no conflict of interests.

**Authors' contributions:** ASK is the primarily responsible for drafting the systematic review with support from RC, and SCL. All authors critically reviewed the systematic review manuscript for its content and approved the final version for submission.

**Acknowledgement**

We would like to extend our acknowledgment to the Wallace Academic Editing for their editing service of this manuscript.

**Funding**: No fund was received.

**Data availability:** All data generated or analyzed during this study are included in this published article.

**Abstract**

This systematic review and meta-analysis aimed to estimate the overall prevalence of depression and identify related factors among older people in Ethiopia.

Multiple databases with no date limit were searched. The Preferred Reporting Items for Systematic Reviews and Meta-Analyses (PRISMA) was also utilized. The quality of the included studies was assessed using the Joanna Briggs Institute quality appraisal tool. The presence of publication bias was evaluated by performing Egger’s test and a visual inspection of the symmetry in funnel plots.

The overall prevalence of depression among older adults in Ethiopia was 41.85 (95% CI = 33.52, 50.18). Relative to other regions of Ethiopia, the Oromia region had a higher prevalence of depression at 48.07% (95% confidence interval [CI] = 35.62, 60.51). Female older adults (adjusted odds ratio [AOR] = 1.76; 95% CI = 1.17, 2.63), older adults with no formal education (AOR = 1.82; 95% CI = 1.03, 3.19), older adults with chronic diseases (AOR = 2.46; 95% CI = 1.00, 6.06), and with low or no social support (AOR = 2.01; 95% CI = 1.06, 3.83) had a significantly greater prevalence of depression.

Our systematic review and meta-analysis revealed that almost two out of five older adults had depression. Female sex, absence of formal education, having chronic diseases, and low or no social support were the independent predictors of depression among older adults in Ethiopia. Our systematic review and meta-analysis highlight that depression among older adults in Ethiopia is a public health problem.

**Keywords**: Depression, prevalence, predictors, risk factors, older adults, elderly, Ethiopia

**Background**

Depression is a common mental disorder that is characterized by low mood, loss of interest or pleasure, decreased energy, feelings of guilt or low self-worth, disturbed sleep or appetite, and poor concentration (114,115). Older people may not exhibit obvious symptoms of depression. Instead, they may feel tired, have difficulties sleeping, feel irritable, feel confused, have difficulties concentrating, fail to enjoy activities that they used to, move slowly, and experience changes in weight or appetite, in addition to feeling hopeless, worthless, or guilty (116). These symptoms are common in older people, and they are often overlooked during the early stage of depression in an older adult.

Depression is a leading cause of disability worldwide, and it is the fourth largest contributor to the overall global burden of disease (117). It affects people of all genders, ages, and backgrounds in communities worldwide (118–120). The 2021 Institute of Health Metrics and Evaluation report revealed that approximately 280 million people worldwide have depression (121). Studies from middle and high income countries (122,123) have revealed that depression is more common among older people than among adolescents. It is a common illness worldwide and affects an estimated 3.8% of the global population, of which 5.7% are adults older than 60 (124).

Depression is a common mental health disorder among older adults, and it is a major public health problem (125). However, it is a treatable medical condition that is not a normal part of aging. When people age, they will develop various comorbidities. Eighty percent of older adults have at least one chronic health condition, and 50% have two or more. In addition, when people age, they often spend more time alone. Therefore, older adults are at an increased risk for depression (126–128). Moreover, depression reduces an individual’s productivity and ability to engage in daily activities of life, and severe depression can even lead to suicide (129).

Research has identified multiple factors that are associated with depression in older adults (130). Sociodemographic factors such as older age, female sex, widowhood, a separated/divorced marital status (96,131), lack of formal education, living alone (75), and poor economic status have been highlighted by multiple studies (132–134). In addition, low quality of life (130–132), psychological stress (135,136), functional impairment (130,132,137), poor social support (96), chronic diseases (75,136), and cognitive impairment (98,136) have been identified as risk factors for depression among older people.

In Ethiopia, depression contributes approximately 6.5% of the country’s disease burden. Depression is regarded as the third most common disease burden in Ethiopia (138). This is the highest proportion of burden compared with other forms of mental disorders (133). Most studies on depression in Ethiopia have focused on adults with various medical conditions (119,139–142) and not on older people. Although depression is a major public health problem among older adults in Ethiopia, few studies have explored this topic (143), and the few that did have reported an epidemiologic variation of depression among older adults that was between 26.7 % (144) and 68.10% (98) and that has varied over time and across geographical regions.

In addition to the lack of studies on depression among older adults, studies have failed to pinpoint the overall magnitude of depression and its main predictors for the development of appropriate measures. Thus, a systematic review and meta-analysis is required to estimate the overall magnitude of depression among older adults in Ethiopia. Our study addressed the following two research questions. 1. What is the pooled prevalence of depression in Ethiopia? 2. What are the factors of depression in the older adults living in different regions of Ethiopia?

**Methods**

**Reporting**

The Preferred Reporting Items of Systematic Reviews and Meta-Analysis (PRISMA) checklist was used to report the results of the present systematic review (Supplementary table 1). In addition, the PRISMA flow chart was utilized to show the selection process for the studies included in the present analysis (145).

**Searching strategies**

A search strategy was developed under the guidance of an experienced university librarian. Studies and gray literature in the subject area that reported on outcomes of interest were identified. The PubMed, Web of Science, SCOPUS, CINAHL, **PsycInfo**, WHO Global Index Medicus, and Hinari databases were searched to identify relevant studies published between these databases’ inception and October 20, 2021. In addition, the research repositories of Addis Ababa University and Bahir Dar University were searched using the search terms “prevalence,” “magnitude,” “epidemiology,” “factors,” “predictors,” “depression,” “elderly,” “old age,” and “Ethiopia.” Search strings were established using “AND” and “OR” Boolean operators. We also checked the reference lists of the retrieved studies for relevant studies that could be included in the current review. In addition, a manual search was conducted using the names of each region of Ethiopia.

**Eligibility criteria**

**Types of studies**

Original research studies that reported the prevalence and or predictors of depression in older adults in Ethiopia were included. No restriction was imposed for the date of publication of studies. Thesis reports or dissertations that reported outcome variables were not available during our search. Studies that were published as review articles, conference abstracts, editorials, commentaries, and articles without full text were not considered.

**Types of participants, outcomes, and context**

In the present systematic review, the included study participants were older adults, defined as individuals aged 60 years or older (146,147). In this review, studies were included if they examined depression as an outcome of interest or as a risk factor among older adults living in Ethiopia. The review considered studies that were conducted in community settings, outpatient clinics of hospitals, and residential aged care facilities.

**Assessment of methodological quality**

Relevant studies were also evaluated for methodological quality before they were included. The two reviewers (ASK and HCC) independently assessed the risk of bias in each study by using the Hoy 2012 tool (148), which was designed to assess the quality of prevalence studies. The Joanna Briggs Institute (JBI) critical appraisal checklist, which is a standardized critical appraisal instrument for assessing prevalence studies (149), was utilized to appraise the methodological quality of the selected studies. The checklist comprises nine items. A score was assigned for each item (0 for a “not reported” or “not appropriate” response and 1 for a “yes” response); the scores of the items were summed to obtain a total quality score of between 0 and 9. Studies were then classified as being of low, moderate, and high quality when the obtained score was between 0 and 4, between 5 and 7, and either 8 or 9, respectively (150). Studies that had high or moderate quality were included in the final analysis (Table 1). Any disagreements between the reviewers were resolved through discussions or consultations with the third reviewer (SCL) until a consensus was reached.

**Data extraction**

Data were extracted from the included studies by two independent reviewers (ASK and HCC). A standard data extraction form was designed and pilot tested before it was applied to each included study (151). The extracted data included data on the setting, study design, sampling methods, number of study participants, age range, mean age, outcome measurements, and outcome status of each study.

**Data analysis**

A statistical meta-analysis was conducted to analyze the collected data and determine the pooled prevalence of depression among older adults in Ethiopia. The data were analyzed using STATA version 14.0 (STATA Corporation, College Station, Texas, USA) software. Heterogeneity across the studies was assessed using the *I*^2^ test and Cochran’s *Q* test (152). The thresholds of 25%, 50%, and 75% were used to indicate low, moderate, and severe heterogeneity, respectively (153,154).

The included studies were summarized and presented using a table and a forest plot. Using *I*^2^ test and Cochrane *Q* statistics (155), we evaluated the presence of potential heterogeneity among the included studies.

The collected data were pooled, and the meta-analysis results provided a summary of depression statistics with 95% confidence intervals (CIs). Consequently, the individual study proportions were listed with their 95% CI values. Because we observed heterogeneity across the studies, a subgroup analysis was performed to assess the contribution of each study to overall heterogeneity. The presence of publication bias was evaluated by performing Egger’s test and a visual inspection of the symmetry in funnel plots (152,156,157).

**Results**

**Search results**

In the present review, 1615 studies were retrieved by performing multiple forms of electronic searches on multiple databases. Among the aforementioned studies, 1470 were excluded because they were duplicates, 75 studies were excluded after their titles were reviewed, 43 studies were excluded after their titles and abstracts were reviewed, and 16 studies were excluded for other reasons. Finally, 11 studies were included in the present systematic review and meta-analysis. The inclusion process was conducted in accordance with the PRISMA flowchart (Figure 1).

**Characteristics of included studies**

In the present systematic review and meta-analysis, 11 full-text studies were included. The included studies all utilized a cross-sectional study design. The number of study participants in each study ranged from 116 in a study conducted in Addis Ababa (98) to 941 in a study conducted in Amhara region (143); the included studies had a total of 6521 study participants. The included studies were published between 2016 (97) and 2021 (75,143,144). Four regional states (counties) of Ethiopia were represented in the studies. Four studies were conducted in the Amhara region (96)(143)(158)(159), three studies conducted in Addis Ababa (91,98,144), two studies were conducted in Oromia (75,125) and two studies were conducted in Harar (97,160). Three studies (91,98,160) did not report the mean age of their study participants, whereas the mean age of the participants in the other included studies ranged from 66.69 to 75.46 years. The response rate of the included studies ranged from 93.4% (160) to 100% (75,98). All the included studies utilized the Geriatric Depression Scale-15 (GDS-15) as their outcome diagnostic measurement tool. Two studies recruited their participants from a residential aged care facility (98) and the outpatient clinic of a referral hospital (144), respectively, whereas the rest of the studies were conducted in community settings (Table 2).

**Prevalence of depression among older adults in Ethiopia**

Eleven studies were incorporated to estimate the overall prevalence of depression among older adults in Ethiopia. In the included studies, the minimum prevalence and maximum prevalence of depression among older adults were 26.7 (144) and 68.1 (98), respectively. The overall prevalence of depression among older adults in Ethiopia was 41.85 (95% CI = 33.52, 50.18; *I*^2^ = 93.3%; P < 0.001; Figure 2). Because the test statistics revealed a considerable heterogeneity (161) across the studies (*I*^2^ = 93.3%, P < 0.001), a random effects model was used to estimate the Der Simonian and Laird’s pooled effect. To minimize the random variations between the point estimates of the included studies, a subgroup analysis was performed.

**Subgroup analysis**

Because of a high level of heterogeneity across the included studies, a subgroup analysis was performed by region (county) and study settings in relation to outcome variables. The analysis revealed that the prevalence of depression among older adults was highest in the Oromia region (48.07%; 95% CI = 35.62, 60.51) followed by the Amhara region (45.72%; 95% CI = 32.43, 59.02) and Addis Ababa (40.68%; 95% CI = 16.36, 64.99), respectively (Figure 3). Older adults who were recruited from institutions exhibited a higher rate of depression with a prevalence of 47.32% (95% CI = 6.74, 87.89), whereas older adults in community-based settings exhibited a lower rate of depression with a prevalence rate of 37.92% (95% CI = 25.9, 49.93; Supplementary figure 1).

**Meta-regression**

In addition to conducting the subgroup analysis, we also conducted meta-regression to identify the source of heterogeneity. A meta-regression analysis was conducted using the study variables of year of publication, number of study participants, and prevalence. However, its results revealed that none of the aforementioned variables were significant sources of heterogeneity.

**Publication bias**

Two methods for detecting publication bias in systematic reviews and meta-analysis were used. The funnel plot was visually inspected for publication bias (Figure 4). In addition, publication bias was also objectively examined using Egger’s weighted correlation and Begg’s regression intercept test at a 5% significance level (152,156,157,162,163). The test results indicated the absence of significant publication bias (β = –0.22; standard error = 2.37; P = 0.93; Supplementary table 2).

**Factors associated with depression among older adults**

In the present systematic review, seven of the included primary studies reported on factors that are associated with depression among older adults. From those studies, we extracted multiple factors. Among the extracted factors, having a chronic disease, having a lack of formal education, being female, being retired, being widowed, being divorced, and having low or no social support were associated with depression among older adults. Four studies (96,97,125,143) reported that female older adults were more depressed than male older adults. Three studies (96)(75)(97) reported that older adults with chronic diseases were more depressed than those without such diseases. Four studies (75,96,143,144) reported that older adults who had low or no social support were more depressed than those who received greater levels of social support. Two studies (75,97) reported that older adults who did not receive formal education were more depressed than those who did.

In the present systematic review and meta-analysis, being female (AOR = 1.76; 95% CI = 1.17, 2.63; Supplementary figure 2), having a lack of formal education (AOR = 1.82; 95% CI = 1.03, 3.19; Supplementary figure 3), having chronic diseases (AOR = 2.46, 95% CI = 1.00, 6.06; Supplementary figure 4), and having low or no social support (AOR = 2.01; 95% CI = 1.06, 3.83; Supplementary figure 5) were revealed to be independent predictors of depression among older adults in Ethiopia.

**Discussion**

The present study is the first systematic review and meta-analysis to estimate the pooled prevalence of depression and its associated risk factors among older adults in Ethiopia. In this systematic review and meta-analysis, 6521 older adults were included. The World Health Organization estimated that the overall prevalence rate of depressive disorders among the older adults in a given region or country generally varies between 10% and 20%, depending on cultural factors (164). However, our study revealed that the pooled estimated prevalence of depression among older adults in Ethiopia was 41.85 (95% CI = 33.52, 50.18; *I*^2^ = 93.3%; P < 0.001). This finding is consistent with that of a study conducted in a neighboring country, Sudan, which reported that 41.1% of older adults had depressive symptoms (165). The results of our study are also relatively consistent with those of studies conducted in other African countries. Studies conducted in Ghana, Egypt, and Tanzania have revealed that 37.8% (166), 44.4% (167), and 44.4% (168), respectively, of older adults had depressive symptoms. Furthermore, our study results are relatively consistent with those of a study that was conducted in Iran and that reported a depression prevalence of 43% among older adults (169).

Compared with our findings, those of other Africa-based studies have indicated a higher prevalence of depression among older adults. Studies conducted in Nigeria have indicated that between 45.5% (170) and 52% (171) of older adults have depression. The older adults examined in these Nigeria-based studies exhibited high functional disability (62), and more than one fifth of them were living alone (63); these are factors that can increase the likelihood of depression in older adults. A study from South Africa also revealed that 51.9% of older adults had depressive symptoms (172). These older adults in South Africa were living with a human immunodeficiency virus infection or acquired immunodeficiency syndrome, which might have increased their likelihood of developing depression. Another study conducted in Egypt indicated that 62.7% of older adults had depressive symptoms (67).

In contrast to the higher prevalence of depression observed in the aforementioned African countries, lower rates of depression were observed among older adults in other African countries. A study that examined populations in sub-Saharan countries revealed that 9.2% (173) of older adults had depressive symptoms. Compared with our study, this sub-Saharan study included older adults who were aged 50 years or older and recruited them from multiple countries; these older adults varied in their socioeconomic characteristics and lifestyle. Studies conducted in Tanzania and Kenya have also revealed that 16.2% (174) and 22.9% (175), respectively, of older adults had depressive symptoms. In addition to the study setting–related and age-related differences between these studies and the present review study, differences in methodology were also noted. A South Africa–based study also reported that only 4% of older adults had depression (176). This study had apparently recruited healthy community-based older adults who were aged 50 years or older. All the aforementioned factors might have contributed to the difference in the prevalence of depression among the participants of the South Africa–based study and that reported in our study.

Studies from Europe and Asia have also reported a high prevalence of depression among older adults. A study conducted in Vietnam reported that 66.9% of older adults had depressive symptoms (177). A study of older ethnic Turkish adults in the Netherlands revealed that 61.5% of them had depression (178). In addition to variations in the tools used to measure outcome variables, these older adults in the Netherlands were migrants from Turkey. These migrant older people might have experienced diverse biopsychosocial constraints that caused their depression. A study from Palestine revealed that 51.9% of its study participants had depressive symptoms (179); in contrast to our study, it recruited older adults from refugee camps, which was a factor that could have contributed to the higher prevalence of depression among the older adults who were examined in that study. A study conducted in Nepal reported that 57.8% of older adults had depressive symptoms (180). This Nepal study recruited older adults who were not cared for by their children, which is a factor that could have increased the incidence of depression among these older adults. By contrast, most of the older adults that were included in our study were recruited from communities and had social connections.

However, other studies from various countries in Asia and Europe have revealed a low prevalence of depression among older adults. A study conducted in Europe revealed that 12.3% of older adults had depressive symptoms (181). A Malaysia-based (182) study reported that 7.6% of older adults had depressive symptoms. A study from Japan (183) reported that 20.5% of older adults had depression. In addition to cultural and lifestyle differences, those studies utilized various diagnostic instruments to measure outcome variables. In the aforementioned studies, which were conducted in high-income and middle-income countries, the older adult participants were recruited from apparently healthy people who lived in community settings. In contrast to our study, a systematic review and meta-analysis conducted in Iran reported that only 8.2% of older adults had depressive symptoms. This considerable difference between the finding of our study and that of the Iran-based study could be attributed to age. The Iran-based study included older adults aged 50 years or older, whereas our study included older adults aged 60 years or older. Furthermore, the Iran-based study only reported on older adults with severe depression. A Beijing-based study reported that 13% of older adults suffered from depression (75). All of these reasons could have contributed to the lower levels of depression among the older adults reported by that study relative to those reported in our study (184).

Depression is the result of a complex interaction of social, psychological, and biological factors (117). Our study revealed that being female is a risk factor for depression among older adults. This finding is consistent with those reported by studies conducted in Kenya (175), Nigeria (171), Ghana (185), Egypt (186), Iran (169), and the Netherlands (178), all of which also reported a significant association between female sex and depression among older adults. Our finding also revealed that older adults with no formal education were more likely to have depressive symptoms. A study from Palestine also indicated that low educational status (179) was a predictor for depression among older adults. This systematic review and meta-analysis revealed that older adults with chronic disease were more likely to have depressive symptoms. This finding is also consistent with those of studies conducted in South Africa (176), China, (184) and the Netherlands (178), all of which reported that older adults living with chronic medical conditions were more likely to have depressive symptoms. In our study, we discovered that older adults with no or low social support were more likely to have depressive symptoms. Similarly, studies from Japan (183) and Tanzania (168) have also indicated that older adults with favorable social support were less likely to experience depression.

A further challenge for research and clinical practices related to the diagnosis of depression in older adults involves the diagnosis of depression in individuals with cognitive impairment. Because the prevalence of cognitive impairment increases with age, the differential diagnosis of depression and dementia becomes increasingly difficult with age. Our review identified only two studies (97)(159) that assessed cognitive impairment among older people. The underlying neuropathological conditions that lead to mild cognitive impairment (MCI) or dementia may also contribute to depressive symptoms; thus later-life depression, MCI, and dementia may fall in a clinical continuum (187).

**Conclusion and recommendation**

Our systematic review and meta-analysis revealed that depression among older adults in Ethiopia is now a public health problem, and appropriate screening and interventions should be implemented to reduce its occurrence and considerable effects on older people in Ethiopia. Our findings also revealed that being female, having a lack of formal education, having chronic diseases, and having low or no social support were independent predictors of depression among older adults in Ethiopia.

The WHO developed brief psychological intervention manuals for depression that lay workers can apply to individuals and groups (117). Therefore, healthcare facilities and health care professionals in Ethiopia should use these manuals in health care facilities in primary health care, community, and other settings. Furthermore, information on cognition and functional status is essential, and an appropriate diagnostic framework for depression in cognitively impaired older individuals is required.

**Strength & limitation**

The included studies utilized a consistent set of diagnostic instruments to measure outcome variables, and this helped them to produce unbiased estimates of the overall prevalence of depression among older adults in Ethiopia. The overall prevalence of depression among older adults was derived only from studies conducted in four regional states (counties) of Ethiopia. Therefore, the findings of the present study should be interpreted with caution because the included studies only examined fewer than half of all the regional states of Ethiopia.

**Ethics**

Because this study is a systematic review, ethical approval was not required. The included patients or individuals were not involved in the development of the study protocol.

**Author’s contribution**

ASK is primarily responsible for drafting the systematic review protocol with support from HCC and SCL. All authors critically reviewed the systematic review protocol for its content and approved the finalized version of the systematic review protocol for submission.

**Acknowledgement**

We would like to extend our acknowledgment to the Wallace Academic Editing for their editing service of this manuscript.

***Disclosure statement*Conflict of interests:** The authors have no potential conflicts of interest to disclose.

**Authors' contributions:** ASK is the primarily responsible for drafting the systematic review with support from RC, and SCL. All authors critically reviewed the systematic review manuscript for its content and approved the final version for submission.

**Funding**: No fund was received.

**Data availability:** All data generated or analyzed during this study are included in this published article.

Reference

1. Velkoff VA KP. Aging in Sub-Saharan Africa: The Changing Demography of the Region. National Academies Press (US). 2018. 55–92 p.

2. Jacksonvilli University. Nurses Caring for Geriatric Patients. 2008. p. 3.

3. United Nations Population Fund. Ageing in the Twenty-First Century : A Celebration and A Challenge. 2012.

4. Commissioner UH rights O of the high. United Nations Principles for Older Persons. 1991.

5. Kelly G, Mrengqwa L, Geffen L. “They don’t care about us”: older people’s experiences of primary healthcare in Cape Town, South Africa. BMC Geriatr [Internet]. 2019 Dec 4;19(1):98. Available from: https://bmcgeriatr.biomedcentral.com/articles/10.1186/s12877-019-1116-0

6. Pillemer K, Burnes D, Riffin C et al. E. Elder abuse: global situation, risk factors, and prevention strategies. Gerontol 2004;44469-78. 2016;56:196–205.

7. Evans JM, Kiran PR BOA. Activating the knowledge-to-action cycle for geriatric care in India. Heal Res Policy Syst. 2011;9(42).

8. Yaya S, Idriss-wheeler D, Sanogo NA, Vezina M, Bishwajit G. Self-reported activities of daily living , health and quality of life among older adults in South Africa and Uganda : a cross sectional study. 2020;1–11.

9. Smith M, Saunders R, Stuckhardt L et al. Imperative: Managing Rapidly Increasing Complexity. National Academies Press. National Academies Press (US). 2013.

10. Guarinoni M, Petrucci C, Lancia L et al. The Concept of Care Complexity: A Qualitative Study. J Public Heal Res. 2015;13(3).

11. Aging in Sub-Saharan Africa: Recommendations for Furthering Research. Aging in Sub-Saharan Africa. 2006.

12. Schatz E, Seeley J. Gender. Gender, ageing & carework in east and southern Africa: a review. Glob Public Heal. 2015;10:1185–200.

13. Fried LP, Tangen CM, Walston J et al. Frailty in older adults: evidence for a phenotype. J Gerontol Ser. 2001;56(3):2001.

14. Luciana Correia Alves, Yeda Aparecida de Oliveira Duarte JLFS. Factors Associated the Transitions in the Frailty States Among Elderly in Brazil 2006-2010. 2018. p. 1–16.

15. Espinoza SE, Jung I, Hazuda H. Frailty transitions in the San Antonio Longitudinal Study of Aging. J Am Geriatr Soc. 2012 Apr;60(4):652–60.

16. Uchmanowicz I, Chudiak A, Jankowska-pola B, Gobbens R. Hypertension and Frailty Syndrome in Old Age : Current Perspectives. Card Fail Rev. 2017;3(2):102–7.

17. Gobbens RJ, Assen MA Van, Luijkx KG, Schols JM. Testing an integral conceptual model of frailty. J Adv Nurs. 2011;1–14.

18. Theou O, O’Connell MDL, King-Kallimanis BL, O’Halloran AM, Rockwood K, Kenny RA. Measuring frailty using self-report and test-based health measures. Age Ageing. 2015 May;44(3):471–7.

19. Chamberlain AM, Sauver JLS, Jacobson DJ, Manemann SM, Fan C, Roger VL, et al. Social and behavioural factors associated with frailty trajectories in a population-based cohort of older adults. 2016;1–10.

20. Siriwardhana DD, Hardoon S, Rait G, Weerasinghe MC, Walters KR. Prevalence of frailty and prefrailty among community-dwelling older adults in low-income and middle- income countries : a systematic review and meta-analysis. BMJ Open. 2018;8:1–17.

21. Fairhall N, Kurrle SE, Sherrington C, Lord SR, Lockwood K, John B, et al. Effectiveness of a multifactorial intervention on preventing development of frailty in pre-frail older people : study protocol for a randomised controlled trial. 2015;1–8.

22. Kim C.O., Lee H.Y., Ho S.H., Park H.S. PCW. Effects of visiting prehabilitation program against functional decline in the frail elderly: A prospective randomized community trial. J Korean Gerontol. 2010;30:1293–309.

23. Lee I.S., Ko Y., Lee K.O. YES. Evaluation of the effects of a frailty preventing multi-factorial program concentrated on local communities for high-risk younger and older elderly people. J Korean Acad Community Heal Nurs. 2012;23:201–11.

24. Marcus-Varwijk AE, Peters LL, Visscher TLS, Smits CHM, Ranchor A V., Slaets JPJ. Impact of a Nurse-Led Health Promotion Intervention in an Aging Population: Results From a Quasi-Experimental Study on the “Community Health Consultation Offices for Seniors.” J Aging Health. 2020;32(1):83–94.

25. Kuhirunyaratn P, Prasomrak P, Jindawong B. Effects of a health education program on fall risk prevention among the urban elderly: A Quasi-experimental study. Iran J Public Health. 2019;48(1):103–11.

26. Viggars RJ, Finney A, Panayiotou B. Educational programmes for frail older people, their families, carers and healthcare professionals. Wien Klin Wochenschr. 2021;(September).

27. Thea Dunn, Julie Bliss IR. The impact of community nurse-led interventions on the need for hospital use among older adults: An integrative review. Int J Older People Nurs. 2021;

28. Goodman C, Davies SL, Dinan S, Tai SS, Iliffe S. Activity promotion for community-dwelling older people: a survey of the contribution of primary care nurses. Br J Community Nurs,. 2011;16(1):12–7.

29. Lee Jong-wook. Chronic Disease and Nursing : What ’ s the issue? 2005.

30. Song MS, Boo S. Effects of a nurse-led multicomponent intervention for frail older adults living alone in a community: a quasi-experimental study. BMC Nurs. 2022;21(1):1–9.

31. Markle-Reid M, Browne G, Gafni A. Nurse-led health promotion interventions improve quality of life in frail older home care clients: Lessons learned from three randomized trials in Ontario, Canada. J Eval Clin Pract. 2013;19(1):118–31.

32. Ha J, Park YH. Effects of a person-centered nursing intervention for frailty among prefrail community-dwelling older adults. Int J Environ Res Public Health. 2020;17(18):1–19.

33. Markle-Reid M, Weir R, Browne G, Roberts J, Gafni A, Henderson S. Health promotion for frail older home care clients. J Adv Nurs. 2006;54(3):381–95.

34. Rice H, Say R, Betihavas V. The effect of nurse-led education on hospitalisation, readmission, quality of life and cost in adults with heart failure. A systematic review. Patient Educ Couns [Internet]. 2018;101(3):363–74. Available from: http://www.ncbi.nlm.nih.gov/pubmed/29102442

35. Corley AG, Thornton CP, Glass NE. The Role of Nurses and Community Health Workers in Confronting Neglected Tropical Diseases in Sub-Saharan Africa: A Systematic Review. PLoS Negl Trop Dis. 2016;10(9):1–24.

36. Semachew A, Belachew T, Tesfaye T, Adinew YM. Predictors of job satisfaction among nurses working in Ethiopian public hospitals, 2014: institution-based cross-sectional study. Hum Resour Health [Internet]. 2017;15(1):31. Available from: http://www.ncbi.nlm.nih.gov/pubmed/28438214

37. PEPFAR F& I. The Global Nurse Capacity Producing and Maintaining a Skilled Nursing Workforce in Ethiopia. 2017.

38. Vellas B, Sourdet S. PREVENTION OF FRAILTY IN AGING. J Frailty Aging. 2017;6(4):174–7.

39. Cameron ID, Fairhall N, Langron C, Lockwood K, Monaghan N, Aggar C, et al. A multifactorial interdisciplinary intervention reduces frailty in older people : randomized trial. 2013;

40. Lohman M, Dumenci L, Mezuk B. Depression and Frailty in Late Life: Evidence for a Common Vulnerability. Journals Gerontol Ser B Psychol Sci Soc Sci [Internet]. 2016 Jul;71(4):630–40. Available from: https://academic.oup.com/psychsocgerontology/article-lookup/doi/10.1093/geronb/gbu180

41. Soysal P, Veronese N, Thompson T, Kahl KG, Fernandes BS, Prina AM, et al. Relationship between depression and frailty in older adults: A systematic review and meta-analysis. Ageing Res Rev [Internet]. 2017 Jul;36:78–87. Available from: https://linkinghub.elsevier.com/retrieve/pii/S1568163717300247

42. Frost R, Nair P, Aw S, Gould RL, Kharicha K, Buszewicz M, et al. Supporting frail older people with depression and anxiety: a qualitative study. Aging Ment Health [Internet]. 2020 Dec 1;24(12):1977–84. Available from: https://www.tandfonline.com/doi/full/10.1080/13607863.2019.1647132

43. Markle-reid M, Browne G. Conceptualizations of frailty in relation to older adults. J Adv Nurs. 2003;44(1):1–11.

44. Kivunja C. Distinguishing between theory, theoretical framework, and conceptual framework: A systematic review of lessons from the field. Int J High Educ. 2018;7(6):44–53.

45. Jabareen Y. Building a Conceptual Framework: Philosophy, Definitions, and Procedure. Int J Qual Methods. 2009;8(4):49–62.

46. David B. Hogan., Chris MacKnight HB et al. Models, definitions, and criteria of frailty. Aging Clin Exp Res. 2003;15(3):3–29.

47. Rockwood K, Mitnitski A. Frailty in Relation to the Accumulation of Deficits. J Gerontol Med Sci. 2007;62(7):722–7.

48. Ii WMB. A Conceptual Framework of Frailty : A Review. J Gerontol Med Sci. 2002;57(5):283–8.

49. Jhon E. et al. Frailty consensus: A call to action. J Am Med Dir Assoc. 2013;14(6):1–16.

50. Walston J, Hadley EC, Ferrucci L, Guralnik JM, Newman AB, Studenski SA, et al. Research agenda for frailty in older adults: Toward a better understanding of physiology and etiology: Summary from the American Geriatrics Society/National Institute on Aging research conference on frailty in older adults. J Am Geriatr Soc. 2006;54(6):991–1001.

51. Wang H, Wang J, Xie B, Liu B, Wang J. Multi-dimensional frailty and its risk factors among older residents in long-term care facilities in Shanghai, China. Int J Nurs Sci. 2021;8(3):298–303.

52. Gobbens RJJ, Luijkx KG, Wijnen-Sponselee MT, Schols JMGA. Towards an integral conceptual model of frailty. J Nutr Health Aging. 2009 Mar;14(3):175–81.

53. Clegg A et al. Frailty in elderly people. Lancet. 2013;381(9868):752–62.

54. Morley J. et al. Frailty consensus: a call to action. J Am Med Dir Assoc. 2013;14(6):392.

55. Bergman H et al. Frailty: an emerging research and clinical paradigm--issues and controversies. The Journals of Gerontology Series. Biol Sci Med Sci. 2007;62(7):731–7.

56. Fried LP et al. Frailty in older adults: evidence for a phenotype. The Journals of Gerontology Series A: Biol Sci Med Sci. 2001;56(3):146–56.

57. Bieniek J, Wilczyński K, Szewieczek J. Fried frailty phenotype assessment components as applied to geriatric inpatients. Clin Interv Aging. 2016;11:453–9.

58. Alves S, Teixeira L, Ribeiro O, Paúl C. Examining Frailty Phenotype Dimensions in the Oldest Old. Front Psychol. 2020;11(March):1–8.

59. Gobbens R.J., Luijkx K.G. W-SMT& SJM. towards an integral conceptual model of frailty. J Nutr Health Aging. 2010;14(175–181).

60. Collard RM, Boter H, Schoevers RA OVR. Prevalence of frailty in community- dwelling older persons: A systematic review. J Am Geriatr Soc. 2012;60:1487–92.

61. Levers M-J, Estabrooks CA, Ross Kerr JC. Factors contributing to frailty: literature review. J Adv Nurs. 2006 Nov;56(3):282–91.

62. Xie B, Larson JL, Gonzalez R, Pressler SJ, Lustig C, Arslanian-Engoren C. Components and Indicators of Frailty Measures: A Literature Review. J frailty aging. 2017;6(2):1–7.

63. Ravaglia G, Forti P, Lucicesare A, Pisacane N, Rietti E, Patterson C. Development of an easy prognostic score for frailty outcomes in the aged. Age Ageing. 2008;37(2):161–6.

64. Oostrom SH Van, A DL Van Der, Rietman ML, Picavet HSJ, Lette M, Verschuren WMM, et al. A four-domain approach of frailty explored in the Doetinchem Cohort Study. BMC Geriatr. 2017;17(196):1–12.

65. F. Béland, H. Bergman, P. Lebel, L. Dallaire, J. Fletcher A. Integrated services for frail elders (SIPA): a trial of a model for Canada. Can J Aging. 2006;25(1):2006.

66. Gobbens RJ et al. Towards an integral conceptual model of frailty. J Nutr Health Aging. 2010;14(3):175–81.

67. WHO. The World Health Organization Quality of Life (WHOQOL). 2012. p. 1–12.

68. Gobbens RJ, Luijkx KG, Wijnen-Sponselee MT et al. Toward a conceptual definition of frail community dwelling older people. Nurs Outlook. 2010;58:76–86.

69. Mousavi Sisi M, Shamshirgaran SM, Rezaeipandari H, Matlabi H. Multidimensional Approach to Frailty among Rural Older People: Applying the Tilburg Frailty Indicator. Elder Heal J. 2019;5(2):92–101.

70. Des Jarlais et. Standards for reporting non-randomized evaluations of behavioral and public health interventions: The TREND statement. Am J Public Health. 2004;94(3):361–6.

71. Birhanie G, Melese H, Solomon G, Fissha B, Teferi M. Fear of falling and associated factors among older people living in Bahir Dar City, Amhara, Ethiopia- a cross-sectional study. BMC Geriatr [Internet]. 2021 Dec 21;21(1):586. Available from: https://bmcgeriatr.biomedcentral.com/articles/10.1186/s12877-021-02534-x

72. Argaw MD, Desta BF, Bele TA, Ayne AD. Improved performance of district health systems through implementing health center clinical and administrative standards in the Amhara region of Ethiopia. BMC Health Serv Res [Internet]. 2019 Dec 19;19(1):127. Available from: https://bmchealthservres.biomedcentral.com/articles/10.1186/s12913-019-3939-y

73. Bahir Dar city administration health office. Bahir Dar City Administration demographic profile. 2021. p. 1.

74. Faul F, Erdfelder E, Buchner A LA. Statistical power analyses using G*power 3.1: tests for correlation and regression analyses. Behav Res Methods [Internet]. 2009;41(4):1149–60. Available from: https://doi.org/10.3758/BRM.41.4.1149

75. Jemal K, Hailu D, Tesfa B, Lama T, Kinati T, Mengistu E. Geriatric depression and quality of life in North Shoa Zone, Oromia region: a community cross-sectional study. Ann Gen Psychiatry. 2021;20(1):1–10.

76. Federal Negarit Gazeta of the Federal Democratic Republic Of Ethiopia. A Proclamation to Provide For Public Servants’ Pension: Proclamation No. 714/2011. 2011.

77. HelpAge International. Vulnerability of Older People in Ethiopia: The Case of Oromia, Amhara and SNNP Regional States. 2013.

78. Federal Ministry of Health, USAID & JOHN SNO W I. Ethiopian Urban Health Extension Program [Internet]. 2018. p. 4. Available from: https://publications.jsi.com/JSIInternet/Inc/Common/_download_pub.cfm?id=22119&lid=3

79. Tilahun H, Fekadu B, Abdisa H, Canavan M, Linnander E, Bradley EH, et al. Ethiopia’s health extension workers use of work time on duty: Time and motion study. Health Policy Plan. 2017;32(3):320–8.

80. Lee IFK, Yau FN, Yim SSH, Lee DTF. Evaluating the impact of a home-based rehabilitation service on older people and their caregivers: A matched-control quasi-experimental study. Clin Interv Aging. 2018;13:1727–37.

81. Haider S, Dorner TE, Luger E, Kapan A, Titze S, Lackinger C, et al. Impact of a home-based physical and nutritional intervention program conducted by lay-volunteers on handgrip strength in prefrail and frail older adults: A randomized control trial. PLoS One. 2017;12(1):1–15.

82. Yu R, Tong C, Ho F, Woo J. Effects of a Multicomponent Frailty Prevention Program in Prefrail Community-Dwelling Older Persons: A Randomized Controlled Trial. J Am Med Dir Assoc [Internet]. 2020 Feb;21(2):294.e1-294.e10. Available from: https://linkinghub.elsevier.com/retrieve/pii/S1525861019306401

83. Wong AKC, Wong FKY. The psychological impact of a nurse-led proactive self-care program on independent, non-frail community-dwelling older adults: A randomized controlled trial. Int J Nurs Stud [Internet]. 2020 Oct;110:103724. Available from: https://linkinghub.elsevier.com/retrieve/pii/S0020748920302108

84. Dedeyne L, Deschodt M, Verschueren S, Tournoy J, Gielen E. Effects of multi-domain interventions in (pre)frail elderly on frailty, functional, and cognitive status: a systematic review. Clin Interv Aging [Internet]. 2017;12:873–96. Available from: http://www.ncbi.nlm.nih.gov/pubmed/28579766

85. Gobbens RJ, Uchmanowicz I. Assessing frailty with the tilburg frailty indicator (TFI): A review of reliability and validity. Clin Interv Aging. 2021;16:863–75.

86. Ma L. Current Situation of Frailty Screening Tools for Older Adults. J Nutr Heal Aging. 2019;23(1):111–8.

87. Gobbens RJ, van Assen MA, Luijkx KG, Wijnen-Sponselee MT SJ. The Tilburg frailty indicator: psychometric properties. J Am Med Dir Assoc. 2010;11(5):2010.

88. Uchmanowicz I, Jankowska-Polańska B, Łoboz-Rudnicka M, Manulik S, Łoboz-Grudzień K, Gobbens RJJ. Cross-cultural adaptation and reliability testing of the Tilburg frailty indicator for optimizing care of polish patients with frailty syndrome. Clin Interv Aging. 2014;9:997–1001.

89. Hailemariam H, Singh P, Fekadu T. Evaluation of mini nutrition assessment (MNA) tool among community dwelling elderly in urban community of Hawassa city, Southern Ethiopia. BMC Nutr. 2016;2(11):1–6.

90. Yordanos Mezemir, Gudina Egata DG and AL. Nutritional status and associated factors among community-dwelling elderly. Nutr Diet Suppl. 2020;12:1–11.

91. Abate T, Mengistu B, Atnafu A, Derso T. Malnutrition and its determinants among older adults people in Addis Ababa, Ethiopia. BMC Geriatr. 2020;20(1):1–9.

92. Institute NN. Nutrition screening - a guide to completing the mini nutritional assessment. 2011.

93. Phillips MB, Foley AL, Barnard R, Isenring EA, Miller MD. Nutritional screening in community-dwelling older adults: A systematic literature review. Asia Pac J Clin Nutr. 2010;19(3):440–9.

94. Mesfin Agachew and DH. Faculty of Health Sciences School of Public Health [Internet]. 2017. Available from: http://146.141.12.21/handle/10539/14452

95. John E and David R. Geriatric nutrition Vol. 8. 2007, taylor and francis group: new york. 602. Taylor Fr Gr. 2007;8(602):2007.

96. Amha H, Fente W, Sintayehu M, Tesfaye B, Yitayih M. Depression and associated factors among old age population in Dega damot district, North West Ethiopia. A cross-sectional study. J Affect Disord Reports. 2020;2(October):100034.

97. Girma M, Hailu M, Wakwoya DA, Yohannis Z, Ebrahim J. Geriatric Depression in Ethiopia: Prevalence and Associated Factors. J Psychiatry. 2016;20(1):1–5.

98. Habte E, Tekle T. Cognitive Functioning among Elders with Symptoms of Depression: The Case of Two Selected Institutionalized Care Centers in Addis Ababa, Ethiopia. Heal Sci J. 2018;12(03):1–7.

99. Sarason IG, Sarason BR, Shearin EN and PG. A brief measure of social support: Practical and theoretical implications. J Soc Pers Relat. 1987;4(4):497–510.

100. Nursing THI for G. Katz Index of Independence in Activities of Daily Living. Best Pract Nurs Care to Older Adults,. 2007;25(2):8–9.

101. Donna McCabe. Katz Index of Independence in Activities of Daily Living (ADL). Director [Internet]. 2019;8(2):1–2. Available from: https://hign.org/consultgeri/try-this-series/katz-index-independence-activities-daily-living-adl%0A

102. Shelkey M, Wallace M. Katz Index of Independence in Activities of Daily Living (ADL). Director [Internet]. 2000;8(2):72–3. Available from: https://www.researchgate.net/publication/12246793_Katz_Index_of_Independence_in_Activities_of_Daily_Living_ADL

103. Birhanie G, Melese H, Solomon G, Fissha B, Teferi M. Fear of falling and associated factors among older people living in Bahir Dar City, Amhara, Ethiopia- a cross-sectional study. BMC Geriatr [Internet]. 2021;21(1):1–12. Available from: https://doi.org/10.1186/s12877-021-02534-x

104. Reba K, Birhane BW, Gutema H. Validity and reliability of the Amharic version of the world health organization’s quality of life questionnaire (whoqolbref) in patients with diagnosed type 2 diabetes in felege hiwot referral hospital, Ethiopia. J Diabetes Res. 2019;2019:1–7.

105. Temesgen WA. Progress of recovery and its associated factors in recent onset Psychosis: a mixed-methods study Worku Animaw Temesgen. 2020.

106. World Health Organization (WHO). WHOQOL_ Measuring Quality of Life [Internet]. WHO. 2012. p. 1–88. Available from: https://apps.who.int/iris/rest/bitstreams/110129/retrieve

107. Sørensen MP, Ravn T, Marušić A, Elizondo AR, Kavouras P, Tijdink JK, et al. Strengthening research integrity: which topic areas should organisations focus on? Humanit Soc Sci Commun. 2021;8(1):1–15.

108. Impact R for development. Effective and ethical research and evaluation [Internet]. 2022. p. 1–5. Available from: https://rdinetwork.org.au/effective-ethical-research-evaluation/ethical-practice-starter- kit/research-merit-integrity/

109. Australian Government: National Health and Medical Research Council. Conduct in human research national statement on ethical conduct in human research [Internet]. Vol. 2007. 2018. 104 p. Available from: www.nhmrc.gov.au/guidelines/publications/e72%0Ahttps://www.nhmrc.gov.au/about-us/publications/national-statement-ethical-conduct-human-research-2007-updated-2018

110. University of Michigan. Research ethics and compliant: Informed Consent Guidelines & Templates [Internet]. 2022. p. 1–4. Available from: https://research-compliance.umich.edu/informed-consent-guidelines

111. The Australian National University. Information Sheets & Consent Forms [Internet]. 2022. p. 1–4. Available from: https://services.anu.edu.au/research-support/ethics-integrity/getting-ethics-approval/information- sheets-consent-forms%0A

112. University of Nevada Research. Research integrity: Maintaining Data Confidentiality [Internet]. 2021. p. 6. Available from: https://www.unr.edu/research-integrity/human-research/human-research-protection-policy- manual/410-maintaining-data-confidentiality

113. Bos J. Research Ethics for Students in the Social Sciences [Internet]. Research Ethics for Students in the Social Sciences. 2020. 1–287 p. Available from: https://link.springer.com/chapter/10.1007/978-3-030-48415-6_7

114. Marina Marcus, M. Taghi Yasamy et al. A Global Public Health Concern: WHO Department of Mental Health and Substance Abuse. 2012.

115. J. Cordes. “Depression” in Encyclopedia of Sciences and Religions,. 2013.

116. Debra Fulghum Bruce. Depression in Older People,. WebMD. 2020. p. 2020.

117. WHO. Depression. Vol. 4. 2021.

118. World Federation for Mental Health. Depression: A global crisis? 2012.

119. Woledesenbet MA, Shumet Mekonen S, Sori LM, Abegaz TM. Epidemiology of Depression and Associated Factors among Asthma Patients in Addis Ababa, Ethiopia. Psychiatry J. 2018;2018:1–7.

120. WHO. Integrating mental health into primary care-A global perspective. Geneva, Switzerland:; 2008.

121. World Health Organization (WHO). Depression. 2021. p. 1–3.

122. Paukert LA, LeMaire A CA. Predictors of depression episodes in older veterans with heart failure. Aging Ment Heal. 2009;13(4):601–10.

123. Quan CH, Rong BD, Chan ZL, Rong JY XO. Chronic diseases and risk for depression in old age: A meta- analysis of published literature. Ageing Res Rev. 2009;10:1016–27.

124. Institute of Health Metrics and Evaluation. Institute of Health Metrics and Evaluation. Global Health Data Exchange (GHDx). 2021.

125. Mirkena Y, Reta MM, Haile K, Nassir Z, Sisay MM. Prevalence of depression and associated factors among older adults at ambo town, Oromia region, Ethiopia. BMC Psychiatry. 2018;18(1):1–7.

126. National Institute of Health. Depression and Older Adults. 2022. p. 1–4.

127. Sparrow, E. P., & Erhardt D. How Do I Know If Is Adhd? Essentials of ADHD assessment for children and adolescents. 2014.

128. Fiske A, Wetherell JL GM. Depression in older adults. Annu Rev Clin Psychol. 2009;5:363–89.

129. WHO. Depression and Other Common Mental Disorders: Global Health Estimates, Geneva. 2017.

130. Peltzer K, Phaswana-Mafuya N. Depression and associated factors in older adults in South Africa. Glob Health Action. 2013;6(1):1–9.

131. Thapa SB, Martinez P, Clausen T. Depression and its correlates in South Africa and Ghana among people aged 50 and above: Findings from the WHO study on global ageing and adult health. African J Psychiatry (South Africa). 2014;17(6):1–11.

132. Gureje O, Kola L AE. Epidemiology of major depressive disorder in elderly Nigerians in the Ibadan Study of Ageing: a community-based survey. Lancet. 2007;370:957–64.

133. Bitew T. Prevalence and risk factors of depression in Ethiopia: a review. Ethiop J Health Sci. 2014;24(2):161–9.

134. Hailemariam S, Tessema F, Asefa M, Tadesse H, Tenkolu G. The prevalence of depression and associated factors in Ethiopia: findings from the National Health Survey. Int J Ment Health Syst. 2012;6:1–11.

135. Moledina SM, Bhimji KM, Manji KP. Prevalence and Associated Factors of Depression in an Asian Community in Dar es Salaam, Tanzania. Psychiatry J. 2018;2018:1–5.

136. Yunming L, Changsheng C, Haibo T, Wenjun C, Shanhong F, Yan M et al. Prevalence and risk factors for depression in older people in Xi’an China: a community-based study. Int J Geriatr Psychiatry. 2012;27:31–9.

137. Li N, Pang L, Chen G, Song X, Zhang J ZX. Risk factors for depression in older adults in Beijing. Can J Psychiatry. 2011;56:466–73.

138. Misganaw A, Melaku YA, Tessema GA, Deribew A, Deribe K, Abera SF et al. National disability-adjusted life years (DALYs) for 257 diseases and injuries in Ethiopia, 1990–2015: findings from the global burden of disease study 2015. Popul Heal Metrics. 2017;15(1):28.

139. Edmealem A, Olis CS. Factors Associated with Anxiety and Depression among Diabetes, Hypertension, and Heart Failure Patients at Dessie Referral Hospital, Northeast Ethiopia. Behav Neurol. 2020;2020.

140. Mossie TB, Berhe GH, Kahsay GH, Tareke M. Prevalence of depression and associated factors among diabetic patients at Mekelle City, North Ethiopia. Indian J Psychol Med. 2017;39(1):52–8.

141. Gebre BB, Anand S, Assefa ZM. Depression and Its Predictors among Diabetes Mellitus Patients Attending Treatment in Hawassa University Comprehensive Specialized Hospital, Southern Ethiopia. J Diabetes Res. 2020;2020.

142. Asmare Y, Ali A. Magnitude and Associated Factors of Depression Among People With Hypertension in Addis Ababa , Ethiopia : A Hospital Based Cross-Sectional Study. Res Sq. 2021;

143. Mulat N, Gutema H, Wassie GT. Prevalence of depression and associated factors among elderly people in Womberma District, north-west, Ethiopia. BMC Psychiatry. 2021;21(1):1–9.

144. Yimer YM, Buli MB, Nenko G, Mirkena Y, Kassew T. The prevalence and determinant factors of self-reported depressive symptoms among elderly people with visual impairment attending an outpatient clinic in Ethiopia. Clin Optom. 2021;13:63–72.

145. Moher D, Liberati A, Tetzlaff J, Altman DG. Preferred reporting items for systematic reviews and meta-analyses: The PRISMA statement. BMJ. 2009;339(7716):332–6.

146. Medicare I of M (US) C to D a S for QR and A in, Lohr KN. The Elderly Population. National Academies Press (US); 1990. 12 p.

147. Orimo H, Ito H, Suzuki T, Araki A, Hosoi T, Sawabe M. Reviewing the definition of “elderly.” Geriatr Gerontol Int. 2006;6(3):149–58.

148. Hoy D, Brooks P, Woolf A, Blyth F, March L, Bain C, et al. Assessing risk of bias in prevalence studies: Modification of an existing tool and evidence of interrater agreement. J Clin Epidemiol. 2012;65(9):934–9.

149. The Joanna Briggs Institute Critical Appraisal tools for use in JBI Systematic Reviews. Checklist for Prevalence Studies. 2016.

150. Adane T, Getawa S. Anaemia and its associated factors among diabetes mellitus patients in Ethiopia : A systematic review and meta- ­ analysis. Endocrinol Diabetes Metab. 2021;1–10.

151. JBI. Joanna Briggs Institute-System for the Unified Management, Assessment and Review of Information (JBI-SUMARI). [Online software program]. Adelaide; JBI; Copyright 2016. 2016.

152. Lin L, Chu H. Quantifying publication bias in meta-analysis. Biometrics. 2018;74(3):785–94.

153. Huedo-Medina TB, Sánchez-Meca J, Marín-Martínez F, Botella J. Assessing heterogeneity in meta-analysis: Q statistic or I 2 Index? Psychol Methods. 2006;11(2):193–206.

154. Lee YH. Overview of the Process of Conducting Meta-analyses of the Diagnostic Test Accuracy. J Rheum Dis. 2018;25(1):3.

155. Rücker G, Schwarzer G, Carpenter JR, Schumacher M. Undue reliance on I 2 in assessing heterogeneity may mislead. BMC Med Res Methodol. 2008;8(79).

156. Egger M, Smith GD, Schneider M, Minder C. Bias in meta-analysis detected by a simple, graphical test. BMJ. 1997 Sep;315(7109):629–34.

157. Soeken, Karen L.; Sripusanapan A. Nursing research: Assessing Publication Bias in Meta- Analysis. Rec Manag J. 2003;52(1):57–60.

158. Mezemir Y, Egata G, Geset D LA. Nutritional Status and Associated Factors Among the Community-Dwelling Elderly Population in. Nutr Diet Suppl. 2020;12:289–99.

159. Bekele GT, Allene MD, Getnet MG, Hunegnaw MT, Janakiraman B. Assessing falls risk and associated factors among urban community dwellers older adults in Gondar town, Northwest Ethiopia 2019: A cross sectional study. Int J Surg Open. 2020;24:177–84.

160. Abdu AO, Yimamu ID, Kahsay AA. Predictors of malnutrition among older adults aged above 65 years in eastern Ethiopia: neglected public health concern. BMC Geriatr. 2020;20(1):1–11.

161. Higgins JPT, Thompson SG. Quantifying heterogeneity in a meta-analysis. Stat Med. 2002 Jun;21(11):1539–58.

162. Sterne JA, Egger M. Funnel plots for detecting bias in meta-analysis: guidelines on choice of axis. J Clin Epidemiol. 2001 Oct;54(10):1046–55.

163. Redina-Gobioff et al. Detecting Publication Bias in Random Effects Meta-Analysis: An Empirical Comparison of Statistical Methods. 2006. p. 1–6.

164. Barua A, Ghosh M, Kar N, Basilio M. Prevalence of depressive disorders in the elderly. Ann Saudi Med. 2011;31(6):620–4.

165. Assil SM, Zeidan ZA. Prevalence of depression and associated factors among elderly Sudanese : a household survey in Khartoum State. EMHJ. 2013;19(5):435–40.

166. Nuworza. Prevalence of geriatric depression in a community sample in Ghana : Analysis of associated risk and protective factors. Arch Gerontol Geriatr. 2018;78:171–6.

167. Elkhawaga GO, Sarraf BB. Depression and its associated factors among elderly : A community-based study in Egypt. Arch Gerontol Geriatr. 2018;77:1–10.

168. Adams DJ, Ndanzi T, Rweyunga AP, George J, Mhando L, Ngocho JS, et al. Aging & Mental Health Depression and associated factors among geriatric population in Moshi district council , Northern Tanzania. Aging Ment Health. 2021;25(6):1–10.

169. Sarokhani D, Parvareh M, Dehkordi AH, Sayehmiri K. Prevalence of Depression among Iranian Elderly : Systematic Review and Meta-Analysis. Iran J Psychiatry. 2018;13(1):55–64.

170. Akosile et al. Depression , functional disability and quality of life among Nigerian older adults : Prevalences and relationships. Arch Gerontol Geriatr. 2018;74:39–43.

171. Igbokwe CC, Ejeh VJ, Agbaje OS, Ifeanachor P, Umoke C, Iweama CN, et al. Prevalence of loneliness and association with depressive and anxiety symptoms among retirees in Northcentral Nigeria : a cross-sectional study. BMC Geriatr. 2020;20(153):1–10.

172. gang hao et al. Social participation and perceived depression among elderly population in South Africa. Clin Interv Aging. 2017;12:971–6.

173. Mckinnon B, Harper S, Moore S. The relationship of living arrangements and depressive symptoms among older adults in sub-Saharan Africa. BMC Public Health. 2013;13:1–9.

174. Mlaki DA, Asmal L, Paddick S, Gray WK, Dotchin C. Prevalence and associated factors of depression among older adults in rural. Int J Geriatr Psychiatry. 2021;36(10):1559–66.

175. Ndetei DM. Prevalence and determinants of depression among patients under the care of traditional health practitioners in a Kenyan setting : Policy implications. Transcult Psychiatry. 2017;54(3):1–8.

176. Peltzer K, Phaswana-mafuya N. Depression and associated factors in older adults in South Africa. Glob Heal Action. 2013;18:23336621.

177. Dao ATM, Nguyen VT, Nguyen H V. Urban Vietnam. Biomed Res Int. 2018;2018:1–10.

178. F.B. Van der Wurff et al. Prevalence and risk-factors for depression in elderly Turkish and Moroccan migrants in the Netherlands. J Affect Disord. 2004;83(1):2004.

179. Badrasawi M, Zidan S. Prevalence and correlates of depressive symptoms in older people in the West Bank , Palestine : cross-sectional study. EMHJ. 2021;27(3):1–9.

180. Chalise HN. Depression among elderly living in Briddashram ( old age home ). Adv Aging Res. 2014;3(1):6–11.

181. Etal JRM. Depression among older people in Europe : the EURODEP studies. World Psychiatry 31. 2004;3(1):45–9.

182. Sherina MS, Rampal L, Mustaqim A. The Prevalence of Depression Among the Elderly in. Med J Malaysia. 2004;59(June 2014):1–6.

183. Ryuta FUKUNAGA et al. Living alone is associated with depression among the elderly in a rural community in Japan. Psychogeriatrics ©. 2012;12:179–85.

184. Li N, Pang L, Chen G, Song X, Zhang J. Risk Factors for Depression in Older Adults in Beijing. Can J Psychiatry. 2020;56(8):1–8.

185. Lloyd-sherlock P, Agrawal S, Amoakoh-coleman M, Adom S. Old age and depression in Ghana : assessing and addressing diagnosis and treatment gaps. Glob Health Action. 2019;12(1).

186. Aly HY, Hamed AF, Mohammed NA. governorate. Saudi Med J. 2018;39(2):185–90.

187. Panza F, Frisardi V, Capurso C, D’Introno A, Colacicco AM, Imbimbo BP, et al. Late-life depression, mild cognitive impairment, and dementia: possible continuum? Am J Geriatr Psychiatry. 2010 Feb;18(2):98–116.

Table 1: Critical appraisal of the included studies, 2021

| **Included articles** | | **Criterion No (items included to appraise prevalence studies).** | | | | | | | | | | **Total (%)** | | **Overall quality** | |
| --- | --- | --- | --- | --- | --- | --- | --- | --- | --- | --- | --- | --- | --- | --- | --- |
| Author | Year | **1** | **2** | **3** | **4** | **5** | **6** | **7** | **8** | **9** | **%** | |  | |  |
| Yimer YM. et al | 2021 | **√** | **√** | **√** | **√** | **√** | **√** | **√** | **√** | **√** | 100 | | High | |  |
| Mulat N. et al | 2021 | **√** | **√** | **√** | **√** | **√** | **√** | **√** | **√** | **√** | 100 | | High | |  |
| Jemal K. et al | 2021 | **√** | **√** | **√** | **√** | **√** | **√** | **√** | **√** | **√** | 100 | | High | |  |
| Abate T. et al | 2020 | **√** | **√** | **√** | **√** | **√** | **√** | **√** | **√** | **√** | 100 | | High | |  |
| Mezemir Y. et al | 2020 | **√** | **√** | **√** | **√** | **√** | **√** | **√** | **√** | **√** | 100 | | High | |  |
| Amha H. et al | 2020 | **√** | **√** | **√** | **√** | **√** | **√** | **√** | **√** | **√** | 100 | | High | |  |
| Bekele GT. et al | 2020 | **√** | **√** | **√** | **√** | **√** | **√** | **√** | **√** | **√** | 100 | | High | |  |
| Abdu AO et al | 2020 | **√** | **√** | **√** | **√** | **√** | **√** | **√** | **√** | **√** | 100 | | High | |  |
| Habte E. & Takele T. | 2018 | X | **√** | X | **√** | X | **√** | **√** | **√** | **√** | 66.7% | | Medium | |  |
| Mirkena Y. et al | 2018 | **√** | **√** | **√** | **√** | **√** | **√** | **√** | **√** | **√** | 100 | | High | |  |
| Girma M. et al | 2016 | **√** | **√** | **√** | **√** | **√** | **√** | **√** | **√** | **√** | 100 | | High | |  |
| √ =Yes, criterion fulfilled, X = No, criterion not fulfilled | | | | | | | | | |  | |  | |  | |

Item 1: Was the sample frame appropriate to address the target population? Item 2: Were study participants sampled appropriately? Item 3: Was the sample size adequate? Item 4: Were the study subjects and the setting described in detail? Item 5: Was the data analysis conducted with sufficient coverage of the identified sample? Item 6: Were valid methods used for the identification of the condition? Item 7: Was the condition measured in a standard, reliable way for all participants? Item 8: Was there an appropriate statistical analysis? Item 9: Was the response rate adequate?

Table 2: Characteristics of the included studies, 2021.

| **Authors name** | **Publication year** | **Region** | **Study setting** | **Study design** | **Sampling method** | **% of female** | **Age rang** | **Mean age** | **No. of study participants** | **Response rate (%)** | **Outcome measures** | **Tot. No of outcome** | **Prevalence (%)** | **Do risk factors reported** |
| --- | --- | --- | --- | --- | --- | --- | --- | --- | --- | --- | --- | --- | --- | --- |
| Yimer YM. et al | 2021 | Addis Ababa | Inst. | Cross sectional | SRS | 36.4 | 60-80+ | 72.63 | 423 | 97.3 | GDS-15 | 113 | 26.7 | Yes |
| Mulat N. et al | 2021 | Amhara | Comnt (U & R) | Cross sectional | MSS | 50.8 | 60-75+ | 69.04 | 941 | 98.1 | GDS-15 | 423 | 45 | Yes |
| Jemal K. et al | 2021 | Oromia | Comnt (U & R) | Cross sectional | MSS | 48.7 | 60-90+ | 75.46 | 882 | 100 | GDS-15 | 481 | 54.5 | Yes |
| Abate T. et al | 2020 | Addis Ababa | Comnt. | Cross sectional | SRS | 63.7 | 65-85+ | NR | 662 | 97 | GDS-15 | 183 | 27.64 | No |
| Mezemir Y. et al | 2020 | Amhara | Comnt. | Cross sectional | SRS | 37.54 | 60-75+ | 69 | 341 | 98.27 | GDS-15 | 214 | 62.8 | No |
| Amha H. et al | 2020 | Amhara | Comnt (U & R) | Cross sectional | SRS | 59.3 | 60-75+ | 68.67 | 813 | 98.78 | GDS-15 | 373 | 45.9 | Yes |
| Bekele GT. et al | 2020 | Amhara | Comnt. | Cross sectional | SRS | 57.8 | 60-80+ | 70.51 | 607 | 95 | GDS-15 | 180 | 29.7 | No |
| Abdu AO et al | 2020 | Harar | Comnt (U & R) | Cross sectional | MSS | 50.8 | 65+ | NR | 592 | 93.4 | GDS-15 | 187 | 31.6 | No |
| Habte E. & Takele T. | 2018 | Addis Ababa | Inst. | Cross sectional | Purposive | 41.40 | 60-85+ | NR | 116 | 100 | GDS-15 | 79 | 68.1 | No |
| Mirkena Y. et al | 2018 | Oromia | Comnt. | Cross sectional | MSS | 45 | 60-75+ | 66.69 | 800 | 94.8 | GDS-15 | 334 | 41.8 | Yes |
| Girma M. et al | 2016 | Harar | Comnt. | Cross sectional | SRS | 61.9 | 60-75+ | 69.56 | 344 | 97.7 | GDS-15 | 98 | 28.5 | Yes |

Comnt (U & R) = Community based study done both urban & rural, Comnt.= Community based study done in town, Inst.= Institution care centers, MSS=Multi-stage sampling, SRS=Systematic random sampling

## Appendix B: Information Sheets & Consent Forms

**Participant information sheet**

**Project title:**

**The effect of nurse-led intervention to decrease frailty status of Ethiopian older adults: A quasi-experimental study**

You are invited to participate in a study supervised by *Hui Chen (Rita) Chang (Dr.), Peta Drury (Dr.), Victoria Traynor (Prof.), and Shu Chun Lee (Dr.)* conducted by *Ayele Semachew Kasa* *(PhD Student*) who is a student in the School of Nursing at The University of Wollongong, Australia.

The overall aim of this study is to design, implement and evaluate the effect of a nurse-led intervention on frailty and associated health consequences among older persons living in Bahir Dar, Ethiopia.

If you agree to be involved, you will be asked to attend approximately a 30 to 40-minutes monthly nurse-led education at your own home for a total of six months. The nurse-led intervention will focus on ageing and age-related changes, healthy nutrition, physical activity, mental health, social support, and in the final session there will be an overall discussion and reflection. Furthermore, you will be interviewed for a paper-based survey and non-invasive physical health measurements (height and weight) just before the start of the first nurse-led intervention session and at the twelfth week of the last session. To assess and measure the required information, different questions will be asked that may take approximately 40 minutes.

There is no risk in participating in this study, except for spending time taking the nurse-led intervention and responding to the study questions. No identifiable information is being collected. Your involvement in the study is entirely voluntary and you may choose not to participate. You have all the rights to ask questions and withdraw from the study before or during the intervention session without consequence of any kind. All information related to you will remain confidential and will be identifiable by codes known only to the researcher.

The research has no direct benefit to you. However, it will provide data on the extent of frailty among older adults and the effectiveness of nurse-led education in reducing frailty status among older adults from an Ethiopian perspective. The finding will inform healthcare policy and community nursing care practices aimed at reducing frailty in older adults’ health assessment and management in community settings.

If you feel uncomfortable during either the interventional sessions or during the interview, the intervention session and the interview can be stopped by you at any time. If needed, you will be referred to a nearby health centre for emotional support or counseling/support services.

It is expected that the results of this project will be published in professional journals. In any publication, information will be provided that the participant (you) cannot be identified. Results from this study will only be reported and published in ways that ensure your identifying information remains confidential.

If you need more information about this study, you are kindly requested to contact Ayele Semachew at telephone number +251-913959205 or his lead supervisor Dr. Rita Chang using [hchang@uow.edu.au](mailto:hchang@uow.edu.au)

If you have any concerns or complaints about the ethical conduct of this study, you may contact the Institutional Review Board (IRB) of College of Medicine and Health Sciences, Bahir Dar University through Tel **+**251 58 220 65 57 or the University of Wollongong (UOW) Ethics Officer +61 2 4239 2191 or email [uow-humanethics@uow.edu.au](mailto:uow-humanethics@uow.edu.au). Any issues you raise will be treated in confidence and investigated fully, and you will be informed of the outcome.

This study has been approved by the University of Wollongong Human Research Ethics Committee with the approval number of *[****The*** ***approval number will be entered once the project has been approved****]* and from the Institutional Review Board (IRB) of College of Medicine and Health Sciences, Bahir Dar University *[****The*** ***approval number will be entered once the project has been approved****].*

If you agree to participate in this study, you may be asked to sign the Participant Consent Form. The information sheet is for you to keep, and the consent form is retained by the researcher/s.

Thank you for your time!

**Participants’ Information Sheet፡ Amharic Version**

**የተሳታፊ መረጃ ወረቀት**

**የፕሮጀክት ርዕስ፡-**

**በኢትዮጵያውያ አረጋውያን ጎልማሶች ላይ ደካማ ሁኔታን ለመቀነስ በነርስ-መር የሚሰጥ ትምህርታዊ ጥናት ውጤታማነት፡-ኳሲ-የጥናት ዘዴ**

በሁይ *ቼን (ሪታ) ቻንግ (ዶ/ር)፣ ፔታ ድሩሪ (ዶ/ር)፣ ቪክቶሪያ ትሬኖር (ፕሮፌሰር) እና ሹ ቹን ሊ (ዶ/ር) ተቆጣጣሪነት* በሚመራው *እና በአየለ ስማቸው ካሳ (የዶክትሬት ተማሪ*) በዎሎንግንግ ዩኒቨርሲቲ፣ አውስትራሊያ የነርስ ትምህርት ቤት ተማሪ በሚደረገው ምርምር ላይ እንዲሳተፉ ተጋብዘዋል።

የዚህ ጥናት አጠቃላይ ዓላማ በነርስ የሚመራ ጥናት በባህር ዳር ኢትዮጵያ ውስጥ በሚኖሩ አረጋውያን ላይ በደካማነት (አካላዊ፤ ስነ-ልቦናዊ እና ማህበራዊ) እና ተያያዥ የጤና ችግሮች ላይ የሚያሳድረውን ተጽእኖ መንደፍ፣ መተግበር እና መገምገም ነው።

በዚህ ጥናት ለመሳተፍ ፈቃድዎ ከሆነ የትም ሳይሄዱ በራስዎ ቤት ከ30 እስከ 40 ደቂቃ የሚፈጅ ወርሃዊ ትምህርት በነርሶች አማካይነት በድምሩ ለስድስት ወራት ይከታተሉ ። በነርስ የሚመራው ስልጠና በእድሜ እና ከእድሜ ጋር በተያያዙ ለውጦች፤ ስለጤናማ አመጋገብ፤ የአካል ብቃት እንቅስቃሴ፤ የአእምሮ ጤና፤ ማህበራዊ ድጋፍ ላይ ያተኩራል, እና በመጨረሻ ወይም በስድስተኛው ወር አጠቃላይ ስለነበረው የስልጠና ሂደት ውይይት እና አስተያዬት ይኖራል:: በተጨማሪም፣ በነርሶች የሚሰጠው የመጀመሪያው ስልጠና ከመጀመሩ በፊት እና የበመጨረሻው ክፍለ ጊዜ ስልጠና ከተሰጠ በአስራ ሁለተኛው ሳምንት ላይ ወደ 40 ደቂቃዎች የሚወስዱ ቃለ-መጠይቆች እና ሌሎች አካላዊ ጤና መለኪያዎች (ቁመት እና ክብደት) ይደረግልዎታል።

በነርሶች የሚሰጠው ስልጠና እና ለጥያቄዎች ምላሽ ለመስጠት የተወሰነ ጊዜዎን ከመውሰዱ በስተቀር በዚህ ጥናት ውስጥ መሳተፍ ምንም አይነት አደጋ የለውም።

በዚህ ጥናት ለመሳተፍ በእርስዎ ሙሉ ነፃ ፈቃድ እና ምርጫ ላይ የተመሰረተ ሲሆን በጥናቱ ያለመሳተፍ እንዲሁም በጥናቱ ያለመሳተፍ መብት አለዎት፡፡

ከጥናቱ በፊት፤ በስልጠናው ወቅት ወይም በኋላ ምንም አይነት መዘዝ ሳይኖረው ጥያቄዎችን የመጠየቅ እና ከጥናቱ የመውጣት ሁሉም መብቶች አሎት። ከእርስዎ ጋር የተያያዙ ሁሉም መረጃዎች ሚስጥራዊ ሆነው በተመራማሪው ብቻ በሚታወቁ ኮዶች ተለይተው ይያዛሉ።

ጥናቱ ለእርስዎ ቀጥተኛ ጥቅም ላይኖረው ይችላል ነገር ግን፣ በአረጋውያን መካከል ያለውን የድክመት (አካላዊ፤ ስነ-ልቦናዊ እና ማህበራዊ) መጠን እና በነርስ የሚመራ ትምህርት በኢትዮጵያዊ አተያይ በአረጋውያን መካከል ያለውን የደካማነት ሁኔታ ለማሻሻል ያለውን ውጤታማነት መረጃ ይሰጣል። ግኝቱ በጤና አጠባበቅ ፖሊሲ እና በማህበረስብ ውስጥ ለሚሰሩ ነርሶች እና ባለሞያዎች በማህበረሰቡ ውስጥ ለሚኖሩ አረጋዊን ድክመትን ለመቀነስ ያለመ የጤና እንክብካቤን ያሳውቃል።

ምቾት የማይሰማዎት ከሆነ ፣ በትምህርት ክፍለ ጊዜው እና በቃለ-መጠይቁ ወቅት በማንኛውም ጊዜ በእርስዎ ፈቃድ ሊያስቆሙ ይችላሉ። ካስፈለገ ለስሜታዊ ድጋፍ ወይም ለምክር/ድጋፍ አገልግሎት በአቅራቢያ ወደሚገኝ የጤና ማእከል እንዲሄዱ ይደረጋል፡፡

የዚህ ፕሮጀክት ውጤት በፕሮፌሽናል መጽሔቶች ላይ እንደሚወጣ ይጠበቃል:: በማንኛውም ህትመቶች ውስጥ የእርስዎን ማንነት የሚገልጹ መረጃዎች በኅትመቱ ላይ አይካተቱም፡፡

ከዚህ ጥናት የሚገኘው ውጤት ማንነትን የሚገልጹ መረጃዎች በሚስጥር እንደተያዙ ይቆያሉ፡፡

ስለዚህ ጥናት ተጨማሪ መረጃ ከፈለጉ አየለ ስማቸውን በስልክ ቁጥር +251-913959205 ወይም ዋና ሱፐርቫይዘሩን ዶ/ር ሪታ ቻንግ’ን በ [hchang@uow.edu.au በመጠቀም እንድታነጋግሩ በትህትና እንጠይቃለን።](mailto:hchang@uow.edu.au%20%20በመጠቀም%20እንድታነጋግሩ%20በትህትና%20እንጠይቃለን።)

በዚህ ጥናት ስነምግባር ላይ የሚያሳስባችሁ ወይም ቅሬታ ካላችሁ በባህር ዳር ዩኒቨርሲቲ ህክምና እና ጤና ሳይንስ ኮሌጅ ተቋማዊ ግምገማ ቦርድ በስልክ ቁጥር **+** 251 58 220 65 57 ወይም በወሎንጎንግ ዩኒቨርሲቲ ማነጋገር ይችላሉ፡፡ (UOW) የስነምግባር ኦፊሰር +61 2 4239 2191 ወይም ኢሜል [uow-humanethics@uow.edu.au](mailto:uow-humanethics@uow.edu.au) . የሚያነሱት ማንኛውም ጉዳይ በምስጢር ይያዛል እና ሙሉ በሙሉ ይመረመራል፣ ውጤቱም ይነገርዎታል።

*ይህ* ጥናት በወሎንጎንግ ዩኒቨርሲቲ የሰው ጥናትና ሥነ ምግባር ኮሚቴ በተፈቀደ ቁጥር ***ጸድቋል*** ***የማረጋገጫ ቁጥር ፕሮጀክቱ ከፀደቀ በኋላ ይመዘገባል*** *]* እና ከባህር ዳር ዩኒቨርሲቲ ህክምና እና ጤና ሳይንስ ኮሌጅ ተቋማዊ ግምገማ ቦርድ *[* ***The*** ***የማጽደቂያ ቁጥር ፕሮጀክቱ ከፀደቀ በኋላ ይገባል*** *።*

በዚህ ጥናት ለመሳተፍ ከተስማሙ፣ የተሳትፎ ስምምነት ቅጽ ላይ እንዲፈርሙ ሊጠየቁ ይችላሉ። የመረጃ ወረቀቱ እርስዎ እንዲያስቀምጡት ነው፣ እና የፈቃድ ቅጹ በተመራማሪው/ዎች የሚያዝ ይሆናል።

ለጊዜዎት አመሰግናለሁ!

**Consent Form**

**Project title:**

**The effect of nurse-led education to decrease frailty status of Ethiopian older**

**persons: A quasi-experimental study**

**Consent to Participate in Research**

I received adequate information in the language that I understand in the research project titled “The effect of nurse-led intervention to decrease frailty status of Ethiopian older persons: A quasi-experimental study” supervised by *Hui Chen (Rita) Chang (Dr), Victoria Traynor (Prof), Peta Drury (Dr) and Shu Chun Lee (Dr)* conducted by *Ayele Semachew Kasa* *(Ph.D. Student*) discussed the research project with me.

I have been made aware of any known or expected inconvenience, risk, or discomfort, and of their implications as far as they are currently known by the researchers.

I understand that my participation in this study will allow the researchers to assess the extent of frailty and examine the effect of nurse-led intervention on frailty status among older adults living in Bahir Dar, Ethiopia.

I consent to participate in the research project and the following has been explained to me:

- The research may not be of direct benefit to me
- My participation is completely voluntary
- My right to ask questions and withdraw from the study at any time without any implications for me.
- What I am expected and required to do
- Whom I should contact for any complaints about the research or the conduct of the research
- Security and confidentiality of my personal information.

Participant’s name______________________________________

Signature __________________Date: ______________________

**Consent to Participate: Amharic Version**

**የስምምነት ቅጽ**

**የፕሮጀክት ርዕስ፡-**

**በኢትዮጵያ በአረጋዊያን ላይ ደካማነትን (አካላዊ፣ ማህበራዊ እና ስነ-ልቦናዊ) ለመቀነስ በነርስ የሚመራ ትምህርታዊ ጥናት፡ ኳሲ-የጥናት ዘዴ**

**በምርምር ውስጥ ለመሳተፍ ፈቃድ**

*ከላይ የተጠቀሰው የምርምር ርዕስ በሁይ ቼን (ሪታ) ቻንግ (ዶ/ር) ቪክቶሪያ ትሬኖር (ፕሮፌሰር)፣ ፔታ ድሩሪ (ዶ/ር) እና ሹ ቹን ሊ (ዶ/ር) ተቆጣጣሪነት እና በአየለ ስማቸው ካሳ (የዶክትሬት ተማሪ* ) አማካይነት እንደሚካሄድ እና ስለምርምር ሂደቱ ከእኔ ጋር ተወያይተናል።

ማንኛውም የሚታወቅ ወይም የሚጠበቅ ምቾት የሚነሳ ወይም አደጋ የሚያስከትል፣ እና ምንም ስጋት እንድለልወ እንዲሁም አንድምታዎቻቸው ተመራማሪዎቹ እስከሚታወቁት ድረስ እንዳውቅ ተደርጌያለሁ።

በዚህ ጥናት ውስጥ መሳተፍ ተመራማሪዎቹ በነርስ የሚመራው ጥናት ደካማነት (አካላዊ፤ ስነ-ልቦናዊ እና ማህበራዊ) በባህር ዳር ኢትዮጵያ በሚኖሩ አረጋዊያን ላይ ያለውን ተጽእኖ ለመመርመር እንደሚያስችላቸው ተረድቻለሁ።

የሚከተሉት ነጥቦች የተብራሩልኝ ሲሆን እኔም በምርምር ፕሮጀክቱ ለመሳተፍ ፈቃደኛ ነኝ::

- ጥናቱ ለእኔ ቀጥተኛ ጥቅም ላይኖረው ይችላል
- የእኔ ተሳትፎ ሙሉ በሙሉ በእኔ ፈቃደኝነት ላይ የተመሰረተ ነው።
- በእኔ ላይ ምንም አይነት እንድምታ ሳይኖር በማንኛውም ጊዜ ጥያቄዎችን የመጠየቅ እና ከጥናቱ የመውጣት መብት አለኝ ።
- የሚጠበቅብኝ እና ማድረግ ስለሚጠበቅብኝ
- ስለ ጥናቱ ወይም ስለ ጥናቱ አፈጻጸም ለሚነሱ ቅሬታዎች ማንን ማግኘት እንዳለብኝ
- ስለ ግልመረጃዬ አስተማማኝነት እና ሚስጥራዊነት።

የተሳታፊው ስም ______________________________________

ፊርማ __________________ ቀን: ______________________

## Appendix C: Data Collection Tools

### Questionnaires: English version

**Participant’s ID number: ____________**

| Tick the corresponding box per the time of data collection | Time of survey | Pre-intervention |  |
| --- | --- | --- | --- |
|  |  | Post-intervention |  |
|  |  |  |  |

**Part I: Socio demographic characteristics**

| **Q No.** | **Question/items** | **Coding and category** |
| --- | --- | --- |
| Q1 | Sex | 1.Male 2. Female |
| Q2 | Age | _________ In year |
| Q3 | What is your marital status? | 1. Married 2. Single  3. Divorce 4. widowed  5. Separated 6. Cohabitated |
| Q4 | What is your highest education level? | **1.** Can’t read and write **4**. High school  **2.** Able to read and write **5.** College and above  **3.** Primary school |
| Q5 | Religion | 1. Orthodox 2. Muslim 3. Protestant 4. Catholic   Other _________ |
| Q6 | With whom are you living? | 1.With spouse  2. With child/ren  3. Both spouse & child/ren  3. Alone  4. Other(specify)_________ |
| Q7 | What is your work status? | 1. Merchant 2. Farmer 3. Livestock production 4. Retired/ No occupation 5. Other_______ |
| Q8 | Do you have caregiver? | 1. Yes 2. No |
| Q9 | How much household income do you earn per month | _____________ETB |

**Part II: History of diseases presence, health care services, and behaviors**

**Choose your best answer that represents your feeling or status.**

| **Q. No.** | **Question/items** | **Coding and category** | **Skip** |
| --- | --- | --- | --- |
| Q10 | Do you have any confirmed medical problem? | 1. Yes 2. No | Q11 |
| Q10a | If “Yes” for Q10, what is/are the medical problem/s? | 1. Hypertension/CHF  2. Diabetic Mellitus  3. Arthritis  4. Asthma  5. Other--------------- | Multiple answers are possible |
| Q11 | Have you had fall history in the past 12 months? | 1. Yes 2. No | Q12 |
| Q11a | If “Yes” for Q12, how many times? | 1. Once 2. Twice 3. >=3 |  |
| Q12 | Do you visit health facility for getting health care in the last 3-month? | 1.Yes  2. No |  |
| Q13 | Do you ever used cigarette smoking? | 1. Yes  2. No | Q14 |
| Q13a | If “Yes” to Q14, how many years do you smoke? | ____________ |  |
| Q13b | If yes to Q14, how many packs per day | ____________ |  |
| Q14 | Do you ever used chew chat? | 1. Yes 2. No | Q15 |
| Q14a | If “Yes” to Q14, how does the frequency of chewing chat? | 1. Rare (≤2 days/week)  2. Sometimes (3-4 days/wk.)  3. Usually (5-6 days/wk.)  4.Always/daily |  |
| Q14b | If “Yes” to Q14, how many years do you chewing chat? | _________________ |  |
| Q15 | Do you ever used alcohol* drinks? | 1. Yes 2. No | Q17 |
| Q15a | If “Yes” for q16, how many bottles per day? |  |  |
| Q16 | Overall, how healthy would you say your lifestyle is? | 1. Healthy 2. Unhealthy 3. I don’t judge |  |
| Q17 | Have you experienced one or more of the following events during the past year? |  |  |
| Q17a | the death of a loved one |  |  |
| Q17b | a serious illness yourself |  |  |
| Q17c | a serious illness in a loved one |  |  |
| Q17d | a divorce or ending of an important intimate relationship |  |  |
| Q17e | a traffic accident |  |  |
| Q17f | a crime |  |  |
| Q17g | Are you satisfied with your home living environment? |  |  |
|  | *Such as: ‘tela’, ‘tej’, ‘katicala/areke’, beer, wine | |  |

**Part III: Tilburg Frailty Indicator (TFI)**

| **S. No** | **Variables** | **Response** |
| --- | --- | --- |
| B1: Physical components | | |
| Q900 | Do you feel physically healthy? | 1. Yes 2. No |
| Q901 | Have you lost a lot of weight recently without wishing to do so? (a lot’ is: 6 kg or more during the last six months, or 3 kg or more during the last month) | 1. Yes 2. No |
| Q902 | Do you experience problems in your daily life due to: |  |
| Q902a | difficulty in walking? | 1. Yes 2. No |
| Q902b | difficulty maintaining your balance? | 1. Yes 2. No |
| Q902c | poor hearing? | 1. Yes 2. No |
| Q902d | poor vision? | 1. Yes 2. No |
| Q902f | lack of strength in your hands? | 1. Yes 2. No |
| Q902g | physical tiredness? | 1. Yes 2. No |
| B2 Psychological components | | |
| Q903 | Do you have problems with your memory? | 1. Yes 2. Sometimes 3. No |
| Q904 | Have you felt down during the last month? | 1. Yes 2. Sometimes 3. No |
| Q905 | Have you felt nervous or anxious during the last month? | 1. Yes 2. Sometimes 3. No |
| Q906 | Are you able to cope with problems well? | 1. Yes 2. No |
| B3: Social components | | |
| Q907 | Do you live alone? | 1. Yes 2. No |
| Q908 | Do you sometimes miss having people around you? | 1. Yes 2. Sometimes 3. No |
| Q909 | Do you receive enough support from other people? | 1. Yes 2. No |

**Part IV: Mini Nutritional Assessment (MNA) tool to assess the nutritional status of elders.**Complete the screen by filling in the boxes with the appropriate numbers.

| **Q. No.** | **Question/items** | **Coding and category** | **Score** |
| --- | --- | --- | --- |
| Q300 | Has food intake declined over the past 3 months due to loss of appetite, digestive problems, chewing or Swallowing difficulties? | 0 = severe decrease in food intake 1 = moderate decrease in food intake 2 = no decrease in food intake |  |
| Q301 | Weight loss during the last 3 months | 0 = weight loss greater than 3kg (6.6lbs) 1 = does not know 2 = weight loss between 1 and 3kg 3 = no weight loss |  |
| Q302 | Mobility | 0 = bed or chair bound 1 = able to get out of bed/chair but does not go out 2 = goes out |  |
| Q303 | Has suffered psychological stress or acute disease in the Past 3 months? | 0= Yes 2= No |  |
| Q304 | Neuropsychological problems | 0 = severe dementia or depression  1 = mild dementia  2 = no psychological problems |  |
| Q305 | Body Mass Index (BMI)=weight in kg/ (height in m2 | 0 = BMI less than 19  1 = BMI 19 to less than 21  2 = BMI 21 to less than 23  3 = BMI 23 or greater |  |
|  | Subtotal screening score for MNA short form | |  |
| Q306 | Lives independently (not in nursing home or hospital) | 1 = yes 0 = no |  |
| Q307 | Takes more than3 prescription drugs per day | 0 = yes 1 = no |  |
| Q308 | Takes more than3 prescription drugs per day | 0 = yes 1 = no |  |
| Q309 | How many full meals does the patient eat daily? | 0 = 1 meal 1 = 2 meals 2 = 3 meals |  |
| Q310 | Selected consumption markers for protein intake  • At least one serving of dairy products (milk, cheese, yoghurt) perday --yes/no • Two or more servings of legumesor eggs per week ---yes/no • Meat, fish or poultry every day--- yes/no | 0.0 = if 0 or 1 yes 0.5 = if 2 yes 1.0 = if 3 yes |  |
| Q311 | Consumes two or more servings  of fruit or vegetables per day? | 0= no  1=yes |  |
| Q312 | How much fluid (water, juice, coffee, tea, milk...) is Consumed per day? | 0.0 = less than 3 cups 0.5 = 3 to 5 cups 1.0 = more than 5 cups |  |
| Q313 | Mode of feeding | 0 = unable to eat without assistance 1 = self-fed with some difficulty 2 = self-fed without any problem |  |
| Q314 | Self-view of nutritional status | 0 = views self as being malnourished 1 = is uncertain of nutritional state 2 = views self as having no nutritional problem |  |
| Q315 | In comparison with other people of the same age, how does the patient consider his / her health status? | 0.0 = not as good 0.5 = does not know 1.0 = as good 2.0 = better |  |
| Q316 | Mid-arm circumference (MAC) in cm | 0.0 = MAC less than 21  0.5 =MAC 21 to 22  1.0 = MAC greater than 22 |  |
| Q317 | Calf circumference (CC) in cm | 0 = CC less than 31  1 = CC 31 or greater |  |
|  | **Total** | |  |
|  | **Total assessment (max. 30 points): Malnutrition indicator score**  ≥ 24 points well-nourished------------------------1  17 to 23.5 points at risk of malnutrition-------2  < 17 points malnourished-------------------------3 | |  |

**Part V: Simplified Nutritional Appetite Questionnaire (SNAQ)**

Below there are four questions that assess your appetite status, and each question has five options to be answered. Choose your best answer from the given five options for how you have felt each question.

| **Q. No.** | **Question/items** | **Coding and category** | **Score** |
| --- | --- | --- | --- |
| Q400 | My appetite is | Very poor ---------------------------1  Poor-----------------------------------2  Average------------------------------3  Good----------------------------------4  Very good----------------------------5 |  |
| Q401 | When I eat | I feel full after eating only a few mouthfuls ---------------------1  I feel full After eating about a third of my meal ----------------2  I feel full After eating over half of a meal-------------------------3  feel full After eating most of the meal----------------------------4  I hardly ever feel full---------------------------------------------------5 |  |
| Q402 | Food tastes | Very bad---------------------------------------------1  Bad----------------------------------------------------2  Average----------------------------------------------3  Good--------------------------------------------------4  Very good--------------------------------------------5 |  |
| Q403 | Normally I eat | Less than one meal a day------------------------1  one meal a day-------------------------------------2  two meals a day------------------------------------3  three meals a day----------------------------------4  more than three meals a day--------------------5 |  |
|  | **Total** | Score <= 14 risk for 5% wt loss within 6 month---------1  Score>14 no risk----------------------------------------------2 |  |

**Part VI: Information on depression using Geriatric Depression Scale Short Form (GDS-15)**

**Choose your best answer for how you have felt each question over the past week.**

| **Q. No.** | **Question/items** | **Coding and category** | **Score** |
| --- | --- | --- | --- |
| Q500 | Are you satisfied with your life? | 1. YES 0. NO |  |
| Q501 | Have you dropped many of your activities and interests? | 1. YES 0. NO |  |
| Q502 | Do you feel that your life is empty? | 1. YES 0. NO |  |
| Q503 | Do you often get bored? | 1. YES 0. NO |  |
| Q504 | Are you in good spirits most of the time? | 1. YES 0. NO |  |
| Q505 | Are you afraid that something bad is going to happen to you? | 1. YES 0. NO |  |
| Q506 | Do you feel happy most of the time? | 1. YES 0. NO |  |
| Q507 | Do you often feel helpless? | 1. YES 0. NO |  |
| Q508 | Do you prefer to stay at home, rather than going out and doing new things? | 1. YES 0. NO |  |
| Q509 | Do you feel you have more problems with memory than most? | 1. YES 0. NO |  |
| Q510 | Do you think it is wonderful to be alive now? | 1. YES 0. NO |  |
| Q511 | Do you feel worthless the way you are now? | 1. YES 0. NO |  |
| Q512 | Do you feel full of energy? | 1. YES 0. NO |  |
| Q513 | Do you feel that your situation is hopeless? | 1. YES 0. NO |  |
| Q514 | Do you think that most people are better off than you are? | 1. YES 0. NO |  |

**Part VII: Social Support Questionnaire_ short form (SSQ-6)**

**Instructions**

The following questions ask you about people in your living environment who provide you with help or support. Each question has two parts. For the first part, list all the people you know, excluding yourself, whom you can count on for help or support in the manner described. Give the person’s initials and their relationship to you (see the example). Do not list more than 1 person for each of the numbers beneath the question. Do not list more than nine persons per question. For the second part, circle how satisfied you are with the overall support you have. If the best answer for a particular question is no one, put a tick in the bracket next to “No one”, but still rate your level of satisfaction.

**Please answer all questions as best you can.**

Key: In Examples and keys in answering the questions:

1. Who can you count on to distract you from your worries when you feel under stress?

No one

1. T. F (Spouse) 3) N.B (Sister) 5) T.N (Relatives) 7) 9)
2. A. B (Brother) 4) R.S (Friend/s) 6) 8)
3. Answer of satisfaction is rated from 6: very satisfied 5: fairly satisfied 4: a little satisfied 3: a little dissatisfied 2: dissatisfied and 1: very dissatisfied

| **Q. No.** | **Questions/items** | **Response** | | | | | | | | | | | | | | | | | | | | | | | | | | | |
| --- | --- | --- | --- | --- | --- | --- | --- | --- | --- | --- | --- | --- | --- | --- | --- | --- | --- | --- | --- | --- | --- | --- | --- | --- | --- | --- | --- | --- | --- |
| Q600 | Who can you count on to distract you from your worries when you feel under stress? | No one 5) Relatives  1) Spouse 6)  2) Brother 7)  3) Sister 8)  4) Friend/s 9) | | | | | | | | | | | | | | | | | | | | | | | | | | | |
|  | How satisfied overall? | 6 | | | | | 5 | | | | | 4 | | | | 3 | | | | | | | 2 | | | | | | 1 |
| Q601 | Who can you really count on to help you feel more relaxed when you are under pressure or tense? | No one 5)  1) 6)  2) 7)  3) 8)  4) 9) | | | | | | | | | | | | | | | | | | | | | | | | | | | |
|  | How satisfied overall? | 6 | | | | 5 | | | | | 4 | | | | | | | 3 | | | | 2 | | | | | | 1 | |
| Q602 | Who accepts you totally, including both your worst and your best points? | No one 5)  1) 6)  2) 7)  3) 8)  4) 9) | | | | | | | | | | | | | | | | | | | | | | | | | | | |
|  | How satisfied overall? | 6 | | | 5 | | | | | | 4 | | | | | | 3 | | | | | 2 | | | | | 1 | | |
| Q603 |  | No one 5)  1) 6)  2) 7)  3) 8)  4) 9) | | | | | | | | | | | | | | | | | | | | | | | | | | | |
|  | How satisfied overall? | 6 | | | 5 | | | | | 4 | | | | | 3 | | | | | | 2 | | | | | 1 | | | |
| Q604 | Who can you really count on to care about you, regardless of what is happening to you? | No one 5)  1) 6)  2) 7)  3) 8)  4) 9) | | | | | | | | | | | | | | | | | | | | | | | | | | | |
|  | How satisfied overall? | 6 | 5 | | | | | 4 | | | | | | 3 | | | | | | 2 | | | | | 1 | | | | |
| Q605 | Who can you really count on to help you feel better when you are feeling generally down-in-the-dumps? | No one 5)  1) 6)  2) 7)  3) 8)  4) 9) | | | | | | | | | | | | | | | | | | | | | | | | | | | |
|  | How satisfied overall? | 6 | | 5 | | | | | 4 | | | | 3 | | | | | | 2 | | | | | 1 | | | | | |
| Q606 | Who can you count on to console you when you are very upset? | No one 5)  1) 6)  2) 7)  3) 8) | | | | | | | | | | | | | | | | | | | | | | | | | | | |
|  | How satisfied overall? | 6 | | 5 | | | | | 4 | | | | 3 | | | | | | 2 | | | | | 1 | | | | | |

**Part VIII: Activity of daily life using Katz Index of Independence in Activities of Daily Living**

| **Q. No.** | **Questions/items** | **Response** |
| --- | --- | --- |
| Q700 | Do you ask supervision, direction, or personal assistance when you bath? | 1. Yes 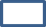 2. No 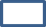 |
| Q701 | Do you ask supervision, direction, or personal assistance when you are dressing? | 1. Yes 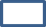 2. No 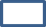 |
| Q702 | Do you ask supervision, direction, or personal assistance when your toileting? | 1. Yes 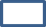 2. No 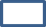 |
| Q703 | Do you asking supervision, direction, or personal assistance when you move and out of bed or moving from bed to chair? | 1. Yes 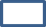 2. No 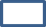 |
| Q704 | Can you control when your urine or bowel is coming till to reach to toilet? | 1. Yes 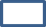 2. No 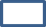 |
| Q705 | Do you move food from plate into mouth without help? | 1. Yes 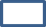 2. No 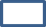 |

**Part IX: The World Health Organization’s Quality of Life Questionnaire (WHOQOL-BREF)**.

**Instructions**

This assessment asks how you feel about your quality of life, health, or other areas of your life. Please answer all the questions. If you are unsure about which response to give to a question, please choose the one that appears most appropriate. This can often be your first response.

Please keep in mind your standards, hopes, pleasures and concerns. We ask that you think about ***your life in the last two weeks.***

**Please read each question, assess your feelings, and circle the number on the scale**

**for each question that gives the best answer for you.**

| Q.No. | Questions/Items | Very poor | Poor | Neither | Good | Very good |
| --- | --- | --- | --- | --- | --- | --- |
| Q1 | How would you rate your quality of life? | 1 | 2 | 3 | 4 | 5 |

|  |  | Very dissatisfied | Dissatisfied | Neither | Satisfied | Very ssatisfied |
| --- | --- | --- | --- | --- | --- | --- |
| Q2 | How satisfied are you with your health? | 1 | 2 | 3 | 4 | 5 |

The following questions ask about **how much** you have experienced certain things in

the last two weeks.

|  |  | Not  at all | A little | A moderate  amount | Very  much | Extreme  amount |
| --- | --- | --- | --- | --- | --- | --- |
| Q3 | To what extent do you feel that physical  pain prevents you from doing what you  need to do? | 1 | 2 | 3 | 4 | 5 |
| Q4 | How much do you need any medical treatment to function in your daily life? | 1 | 2 | 3 | 4 | 5 |
| Q5 | How much do you enjoy life? | 1 | 2 | 3 | 4 | 5 |
| Q6 | To what extent do you feel your life to be  meaningful? | 1 | 2 | 3 | 4 | 5 |
| Q7 | How well are you able to concentrate? | 1 | 2 | 3 | 4 | 5 |
| Q8 | How safe do you feel in your daily life? | 1 | 2 | 3 | 4 | 5 |
| Q9 | How healthy is your physical environment? | 1 | 2 | 3 | 4 | 5 |

The following questions ask about **how completely** you experience or were able to do

certain things in the last two weeks.

|  |  | Not  at all | A little | Moderately | Mostly | Completely | |
| --- | --- | --- | --- | --- | --- | --- | --- |
| Q10 | Do you have enough energy for everyday life? | 1 | 2 | 3 | 4 | 5 | |
| Q11 | Are you able to accept your bodily appearance? | 1 | 2 | 3 | 4 | 5 | |
| Q12 | Have you enough money to meet your  needs? | 1 | 2 | 3 | 4 | 5 | |
| Q13 | How available to you is the information  that you need in your day-to-day life? | 1 | 2 | 3 | 4 | 5 | |
| Q14 | To what extent do you have the  opportunity for leisure activities? | 1 | 2 | 3 | 4 | 5 | |
|  | Questions/Items | Very poor | Poor | Neither | Good | | Very good |
| Q15 | How well are you able to get around? | 1 | 2 | 3 | 4 | | 5 |

The following questions ask you to say how **good or satisfied** you have felt about

various aspects of your life over the **last two weeks**.

|  | Questions/Items | Very dissatisfied | Dissatisfied | Neither | Satisfied | Very ssatisfied |
| --- | --- | --- | --- | --- | --- | --- |
| Q16 | How satisfied are you with your sleep? | 1 | 2 | 3 | 4 | 5 |
| Q17 | How satisfied are you with your ability to perform your daily living activities? | 1 | 2 | 3 | 4 | 5 |
| Q18 | How satisfied are you with your capacity for work? | 1 | 2 | 3 | 4 | 5 |
| Q19 | How satisfied are you with yourself? | 1 | 2 | 3 | 4 | 5 |
| Q20 | How satisfied are you with your personal relationships? | 1 | 2 | 3 | 4 | 5 |
| Q21 | How satisfied are you with your sex life? | 1 | 2 | 3 | 4 | 5 |
| Q22 | How satisfied are you with the support you get from your friends? | 1 | 2 | 3 | 4 | 5 |
| Q23 | How satisfied are you with the conditions of your living place? | 1 | 2 | 3 | 4 | 5 |
| Q24 | How satisfied are you with your access to health services? | 1 | 2 | 3 | 4 | 5 |
| Q25 | How satisfied are you with your transport? | 1 | 2 | 3 | 4 | 5 |

The following question refers to **how often** you have felt or experienced certain things

in the ***last two weeks***.

|  | Questions/Items | Never | Seldom | Quite often | Very often | Always |
| --- | --- | --- | --- | --- | --- | --- |
| Q26 | How often do you have negative feelings such as blue mood, despair, anxiety, depression? | 1 | 2 | 3 | 4 | 5 |

### Questionnaire: Amharic version

**የጥናቱ መጠይቅ**

**የጥናቱ ተሳታፊ መለያ ቁጥር: ____________**

| ጥናቱ በሚሰበሰብበት ወቅት ትይዩ ያለውን ሳጥን ምልክት ይደረግበት | የጥናቱ ጊዜ | ስልጠና ከመሰጠቱ በፊት |  |
| --- | --- | --- | --- |
|  |  | ስልጠና ከመሰጠቱ በኋላ |  |
|  |  |  |  |

**ክፍል 1፡ የማህበራዊ ጉዳዮችን የተመለከቱ ጥያቄዎች**

| **ተ.ቁ** | **የጥያቄ ይዘት** | **የመልስ መለያ ኮድ** |
| --- | --- | --- |
| Q1 | ጸታ | 1. ወንድ 2. ሴት |
| Q2 | እድሜ | _________ ዓመት |
| Q3 | የጋብቻ ሁኔታ | 1. ያገባ/ች 2. ያላገባ/ች  3. የፈታ/ች 4. የትዳር አጋር በሞት የተለየው/ያት  5. ከትዳር አጋር ጋር ተራርቆ/ቃ የሚኖር የምትኖር  6. ካለ ሀጋዊ ትዳር አብሮ የሚኖር/የምትኖር |
| Q4 | የትምህርት ሁኔታ | 1. መፃፍና ማንበብ የማይችል/የማትችል  2. መደበኛ ት/ርት ሳይማሩ መፃፍና ማንበብ የሚችል  3. የመጀመሪያ ደረጃ ት/ርት  4. ሁለተኛ ደረጃ ት/ርት  5. ኮሌጅ እና ከዚያ በላይ |
| Q5 | የየትኛው እምነት ተከታይ ነዎት | 1. ኦረቶዶክስ 2. ሙስሊም 3. ፕሮቴስታንት 4. ካቶሊክ 5. ሌላ ________ |
| Q6 | ቤት ዉስጥ የሚኖሩት ከማን ጋር ነዉ? | 1. ትዳር ጓደኛ  2. ልጆች  3. ለብቻየ  4. ሌላ ---------- |
| Q7 | የመተዳደሪያ ስራዎት ምንድን ነዉ? | 1. ነጋዴ 2. ግብርና 3. እንስሳት ርባታ 4. ጡረተኛ 5. ስራ የለኝም 6. ሌላ_______ |
| Q8 | እርስዎን የሚንከባከበዎት አለዎት? | 1. አዎ 2. የለም |
| Q9 | የእርስዎ የ ወር ገቢ ምን ያክል ነዉ? | _____________ብር |

**ክፍል 2፡ የበሽታ ሁኔታ፤ የጤና አጠዋቀም እና ባህረይ የተመለከቱ ጥያቄዎች**

**ከተሰጡት አማራጮች ውስጥ የእርስዎን ሁኔታ የሚገልፀውን አማራጭ ይምረጡ**

| ተ.ቁ | የጥያቄ ይዘት | የመልስ መለያ ኮድ | **እለፍ** |
| --- | --- | --- | --- |
| Q10 | በህክምና የተረጋገጠ የጤና እክል/ችግር አለበዎት? | 1. አዎ 2. የለም | Q11 |
| Q10a | ለጥያቄ ቁጥር Q10 መልስዎ አዎ ከሆነ፤ የጤና እክሉ/ሎች ምንድን ነው/ናቸው? | 1. የልብ እና ተያያዝ/የደም ግፊት  2. የስኳር  3. የመገጣጠሚያ  4. የመተንፈሻ/አስም  5. ሌላ--------------- | ከአንድ በላይ መመለስ ይቻላል |
| Q11 | ባለፈው 12 ወራት ውስጥ የመውደቅ አደጋ አጋጥሞዎት የውቃል? | 1. አዎ 2. የለም | Q12 |
| Q11a | ለጥያቄ ቁጥር Q11 መልስዎ አዎ ከሆነ፤ ምን ያህክል ጊዜ? | 1. አንድ ጊዜ 2. ሁለት ጊዚ 3. ሶስት ጊዜ እና በላይ |  |
| Q12 | ባለፉት 3 ወራት፣ የጤና እንክብካቤ ለማግኘየት የጤና ተቋም ጎብኝተው ያውቃሉ? | 1.አዎ  2. የለም |  |
| Q13 | በህይዎት ዘመንዎ ሲጋራ አጭሰው ያውቃሉ? | 1.አዎ  2. የለም | Q14 |
| Q13a | ለጥያቄ ቁጥር Q14 መልስዎ አዎ ከሆነ፤ ለምን ያክል ጊዜ አጭሰዋል? | ____________ |  |
| Q13b | ለጥያቄ ቁጥር Q14 መልስዎ አዎ ከሆነ፤ ምን ያክል የሲጋራ ፓኬት? | ____________ |  |
| Q14 | በህይዎት ዘመንዎ ጫት ቅመው ያውቃሉ? | 1.አዎ 2. የለም | Q15 |
| Q14a | ለጥያቄ ቁጥር **Q14** መልስዎ አዎ ከሆነ፤ ተደጋጋሚነቱ ምን ይመስላል? | 1. በጣም አልፎ አልፎ (≤2 days/week)  2. አልፎ አልፎ  3. ብዙ ጊዜ  4.ሁልጊዜ |  |
| Q14b | ለጥያቄ ቁጥር **Q14** መልስዎ አዎ ከሆነ፤ ለምን ያህል ጊዜ አጭሰዋል? | _________________ |  |
| Q15 | አልኮል ጠጥተው ያውቃሉ*? | 1.አዎ 2. የለም | Q17 |
| Q15a | ለጥያቄ ቁጥር **Q15** መልስዎ አዎ ከሆነ፤, በቀን ምንያክል ጠርሙስ/ብርጭቆ ይጠጣሉ? |  |  |
| Q16 | በአጠቃላይ የጤና ሁኔታዎን እንዴት ይገመግሙታል? | 1. ጤናማ 2. ጤናማ ያልሆነ 3. ለመገምገም እቸገራሉ |  |
| Q17 | ባለፈው አንድ አመት ውስጥ የሚከተሉት ክስተቶችን አጋጥሞዎት ያውቃል? |  |  |
| Q17a | የሚወዱት ሰው ሞት | 1. አዎ 2. አያውቅም |  |
| Q17b | በእረስዎ ላይ ከባድ የሚባል ህመም | 1. አዎ 2. አያውቅም |  |
| Q17c | በሚወዱት ሰው ላይ ከባድ የሚባል ህመም | 1. አዎ 2. አያውቅም |  |
| Q17d | ፍቺ ወይም ከአስፈላጊ የቅርብ ወዳጅ ግንኙነት ማቋረጥ | 1. አዎ 2. አያውቅም |  |
| Q17e | የትራፊክ አደጋ | 1. አዎ 2. አያውቅም |  |
| Q17f | ወንጀል | 1. አዎ 2. አያውቅም |  |
| Q17g | በሚኖሩበት የመኖሪያ ቤት ሁኔታና አካባቢ ረክተዋል? | 1. አዎ 2. አይደለም |  |
|  | *Such as: ‘tela’, ‘tej’, ‘katicala/areke’, beer, wine | |  |

**ከፍል 3፡ በአካላዊ፣ ስነልቦናዊ እንዲሁም ማህበራዊ በሆኑ ጉዳዮቸ ላይ ያትኮሩ መጥይቆቸ ናቸው::**

ለእያንዳደዱ ጥያቄዎች ከተሰጡት አማራጮች ውስጥ እኔን ይገልፁኛል የሚሉትን ይምረጡ፡፡

| **ተ.ቁ** | **Variables/መለክያ** | | **ምላሽ** |
| --- | --- | --- | --- |
| **ለ1፡ አካላዊ ክፍል** | | | |
| ጥያቄ 11 | ሰለ አካላዊ ጤናዎ ጤናማ ስሜት ይሰማዎታል? | | 1. አዎ 2. አይደለም |
| ጥያቄ 12 | ሳይፈልጉ ብዙ የክብደት መቀነስ አሳይተዋል? (ብዙ ማለትም 6ኪ.ግ ወይም ከዚያ በላይ በአለፉት ስድስት ወራት ውስጥ ወይም 3ኪ.ግ ወይም ከዚያ በላይ ባለው የመጨረሻ ወር ጊዜ ውስጥ ) | | 1. አዎ 2. አይደለም |
| ጥያቄ 13 | ከዝህ በታች በተገለፁት የዕለት ተዕለት የሕይወትዎ እንቅስቃሴዎች ችግሮች አጋጥሙዎታል፡ | | 1. አዎ 2. አይደለም |
| ጥያቄ 13.1 | ……….መራመድ አለመቻል? | | 1. አዎ 2. አይደለም |
| ጥያቄ 13.2 | ..........ሚዛንዎን ጠብቆ ማቆየት? | | 1. አዎ 2. አይደለም |
| ጥያቄ 13.3 | ..........ደካማ የመስማት ችሎታ? | | 1. አዎ 2. አይደለም |
| ጥያቄ 13.4 | ..........ደካማ የማየት ችሎታ? | | 1. አዎ 2. አይደለም |
| ጥያቄ 13.5 | ...........የእጆችዎ ጥንካሬ እጥረት? | | 1. አዎ 2. አይደለም |
| ጥያቄ 13.6 | …………….የአካል ድካም ? | | 1. አዎ 2. አይደለም |
| **ለ2፡ የስነልቦና ክፍል** | | | |
| ጥያቄ 14 | የማስታወስ ችሎታዎ ላይ ችግሮች አሉብዎት? | 1. አዎ 2. አንዳንድ ጊዜ 3. የለም | |
| ጥያቄ 15 | ባለፈው ወር ውስጥ መጥፎ ስሜት ተሰምቶታል? | 1. አዎ 2. አንዳንድ ጊዜ 3. የለም | |
| ጥያቄ 16 | ባለፈው ወር ወቅት ብስጭት ተሰምቶታል? | 1. አዎ 2. አንዳንድ ጊዜ 3. የለም | |
| ጥያቄ 17 | ችግሮችን በጥሩ ሁኔታ መቋቋም ይችላሉ? | 1. አዎ 2. አይ(አይደለም) | |
| **ለ3: ማህበራዊ ክፍል** | | | |
| ጥያቄ 18 | ብቻዎን ነው ሚኖሩት? | 1. አዎ 2. አይ(አይደለም) | |
| ጥያቄ 19 | አንዳንድ ጊዜ በአካባቢዎ ያሉ ሰዎችን አጥተው ያዉቃሉ? | 1. አዎ 2. አንዳንድ ጊዜ 3. የለም | |
| ጥያቄ 20 | ከሌሎች ሰዎች በቂ ድጋፍ ያገኛሉ? | 1. አዎ 2. አይ(አይደለም) | |

**ክፍል 4፡- የአረጋዊያንን የአመጋገብ ሁኔታ የሚዳስስ መጠይቅ (MNA)**

መጠይቁን እና ልኬቱን ከወሰዱ በኋላ በጥያቄዎቹ ትይዩ ባሉት ሳጥኖቹ ተገቢውን ቁጥር በመሙላት ያጠናቅቁ::

| **ተ.ቁ** | **የጥያቄ ይዘት** | **የመልስ መለያ ኮድ** | **ነጥብ** |
| --- | --- | --- | --- |
| Q300 | ላለፉት 3 ወራት በምግብ ፍላጎት መቀነስ ወይም በሌላ ምክንያት የሚወስዱት የምግብ መጠን ቀንሶ ያዉቃል? | 0= ከፍተኛ የምግብ ፍላጎት መቀነስ  1 = መካከለኛ የምግብ ፍላጎት መቀነስ  2 = ምንም የምግብ ፍልጎት መቀነስ አይታይም |  |
| Q301 | ባለፉት 3 ወራት የነበረ ክብደት መቀነስ ነበር | 0 =ከ3 ኪሎ በላይ የቀነሰ  1 =አላዉቀዉም  2 =የክብደት መቀነስ ከ1-3 ኪሎ  3 =ምንም የክብደት መቀነስ የለም |  |
| Q302 | የእንቅስቃሴ ሁኔታ ምን ይመስላል? | 0 = የአልጋ ቁራኛ 1 = ከአልጋ መነሳት እችላለሁ ግን ወደ ዉጭ መዉጣት አልችልም  2 = ወደ ዉጭ መዉጣት እችላለሁ |  |
| Q303 | ላለፉት 3 ወራት የስነ- ልቦና ጭንቀት ወይም ህመም ገጥሞዎት ያዉቃል? | 0= አዎ  2=የለም |  |
| Q304 | የአእምሮ እና የስነ- ልቦና ችግሮች አሉ? | 0 = ከፍተኛ የሆነ ድብርት ወይም ነገሮችን የመርሳት  1 = መካከለኛ የሆነ የመሳት ችግር  2 = ምንም የአእምሮ ችግር የለም |  |
| Q305 | ክብደት ለቁመት በካሬሜትር (BMI) | 0 = BMI < 19  1 = BMI 19 to < 21  2 = BMI 21 to < 23  3 = BMI ≥23 |  |
|  | Subtotal screening score for MNA short form | |  |
| Q306 | ያለምንም እገዛ እራስዎን ችለዉ እየኖሩ ነዉ? | 0 = አይደለም 1 = አዎ |  |
| Q307 | በቀን ከ3 በላይ የታዘዘ መድሃኒት ይወስዳሉ? | 0 = አዎ 1 = አልወስድም |  |
| Q308 | የቆዳ ላይ ቁስል አለ? | 0 = አዎ  1 = የለም |  |
| Q309 | በቀን የተሟላ ምግብ ስንት ጊዜ ይመገባሉ? | 0 = 1 ጊዜ በቀን 1 = 2 ጊዜ በቀን 2 = 3 ጊዜ በቀን |  |
| Q310 | ለመለኪያነት የተመረጡ የገንቢ ምግቦች   - በቀን አንድ ጊዜ የወተት ተዋጽኦ ይመገባሉ? **አዎ/አልመገብም** - በቀን 2 እና ከዚያ በላይ እንቁላል ወይም ጥራጥሬ ይመገባሉ?  **አዎ/አይ** - ስጋ፣ አሳ በየቀኑ ይመገባሉ? **አዎ/አይ** | 0.0 =0 ወይም 1 አዎ ከሆነ  0.5 = 2 አዎ ከሆነ  1.0 =3 አዎ ከሆነ |  |
| Q311 | በቀን2 እና ከዚያ በላይ አትክልትና ፍራፍሬ ይመገባሉ? | 0= አልመገብም  1=አዎ እመገባለሁ |  |
| Q312 | በቀን ምን ያክል ፈሳሽ(ዉሃ ፣ ወተት፣ሻይ፤ ቡና፤ ጁስ) ይወስዳሉ? | 0.0 = ከ3ብርጭቆ ያነሰ  0.5 = ከ3-5 ብርጭቆ 1.0 = ከ5 ብርጭቆ በላይ |  |
| Q313 | የአመጋገብ ሁኔታ? | 0 = ያለ እገዛ መመገብ አልችልም 1 = ትንሽ ብቸገርም በራሴ እመገባለሁ 2 = ያለ ምንም ችግር እመገባለሁ |  |
| Q314 | የእርስዎን የአመጋገብ ሁኔታ እንዴት ያዩታል? | 0 = በጣም በምግብ የተጎዱ  1 = አላዉቅም/ መካከለኛ በምግብ የተጎዱ  2 = ምንም የምግብ ጉዳት የለም |  |
| Q315 | እራስዎን በእድሜ እኩል ከሆኑ ሌሎች  ሰዎች ጋር ሲያነጻጽሩ የጤናዎ ሁኔታ  ምን ይመስላል? | 0.0 = ጥሩ አይደለም  0.5 = አላዉቅም 1.0 = ጥሩ ነዉ  2.0 = በጣም ጥሩ ነዉ |  |
| Q316 | የመሃል ክንድ ዙሪያ ልኬታ በሴ.ሜ (MAC) | 0.0 = MAC < 21  0.5 =MAC 21 ≤22  1.0 = MAC > 22 |  |
| Q317 | የባት ዙሪያ ልኬታ በሴ.ሜ (CC) | 0 = CC < 31 1 = CC ≥31 |  |
|  | **Total** | |  |
|  | **(ከፍተኛ ነጥብ** = 30)  **የምግብ እጥረት አመላካች ዉጤቶች**  **≥24** ነጥብ= ጥሩ አመጋገብ ያለዉ--------------------------------------------1  **17** to **23.5**=ነጥብ5 ለምግብ እጥረት የተቃረበ/የተጋለጠ-------------------2  **< 17** ነጥብ=የምግብ እጥረት ያለበት-------------------------------3 | |  |

**ክፍል 5፡- የምግብ ፍላጎት ለመዳስስ የተዘጋጀ መጠይቅ**

የሚቀጥሉት አራት ጥያቄዎች የእርስዎን የምግብ ፍላጎት ሁኔታ የሚዳስሱ ሲሆን እያንዳንዱ ጥያቄ አምስት አማራጮች ያሉት ሲሆን ከተሰጡት አማራጮች ውስጥ የእርስዎን የምግብ ፍላጎት የሚወክለውን አማርጭ ይምረጡ፡፡

| **ተ.ቁ** | **የጥያቄ ይዘት** | **የመልስ መለያ ኮድ** | **ነጥብ** |
| --- | --- | --- | --- |
| Q400 | የእርስዎ የምግብ ፍላጎት ምን  ይመስላል | በጣም ዝቅተኛ ----------------------------1  ዝቅተኛ------------------------------------2  መካከለኛ----------------------------------3  ጥሩ----------------------------------------4  በጣም ጥሩ---------------------------------5 |  |
| Q401 | ምግብ በሚመገቡት ወቅት? | ትንሽ ጉርሻ ከጎረስኩ በኋላ እጠግባለሁ-----------------------1  የምግቡን አንድ ሶስተኛ እንደበላሁ እጠግባለሁ---------------2  የቀረበዉን ምግብ ግማሽ እንደበላሁ እጠግባለሁ--------------3  ከቀረበዉ ምግብ አብዛኛዉን እንደበላሁ እጠግባለሁ----------4  በፍፁም ሙሉ የመጥገብ ስሜት አይሰማኝም።----------------5 |  |
| Q402 | የምግቡ ጣእም ምን ይመስላል? | በጣም መጥፎ------------------------------------------1  መጥፎ-------------------------------------------------2  መካከለኛ-----------------------------------------------3  ጥሩ----------------------------------------------------4  በጣም ጥሩ---------------------------------------------5 |  |
| Q403 | በመደበኛዉ በቀን ዉስጥ ስንት ጊዜ ይመገባሉ? | በቀን ከ አንድ በታች--------------------------------1  በቀን አንድ ምግብ----------------------------------2  በቀን ሁለት ምግብ----------------------------------3  በቀን ሶስት ምግብ----------------------------------4  በቀን ከ ሶስት ምግበ በላይ--------------------------5 |  |
|  | **አጠቃላይ ዉጤት** | - አጠቃላይ ዉጤት **<= 14** በሚቀጥሉት 6 ወራት 5% ክብደት የመቀነስ እድል ይኖራል =1 - አጠቃላይ ዉጤት **> 14** በሚቀጥሉት 6 ወራት ክብደት የመቀነስ ስጋት አይኖርም =2 |  |

**ክፍል 6:- የሚቀጥሉት ጥያቄዎች የድብርት ስሜትን ለማወቅ የሚያግዙ ሲሆኑ ባለፈው አንድ ሳምንት ውስጥ የነበረዎትን ስሜት የሚገልፀውን አማረጭ ይመልሱ፡፡**

| **ተ.ቁ** | **የጥያቄ ይዘት** | **የመልስ መለያ ኮድ** |
| --- | --- | --- |
| Q500 | አሁን ባለዎት ህይወት ደስተኛ ነዎት? | 1. አዎ 0. አይደለሁም |
| Q501 | እየሰሩና ሊሰሩ ያሰቧቸው ስራዎችን መስራት አቁመዋል? | 1. አዎ 0. የለም |
| Q502 | ህይወትዎ ሙሉ እንዳልሆነ ይሰማዎታል? | 1. አዎ 0. አይሰማኝም |
| Q503 | ብዙ ጊዜ ቀኑ አሰልቺ ይሆንበዎታል? | 1. አዎ 0. አይሆንብኝም |
| Q504 | አብዛኛውን ጊዜ በጥሩ መንፈስ ነዎት? | 1. አዎ 0. አይደለሁም |
| Q505 | አንድ መጥፎ/ ጥሩ ያልሆነ ነገር ይመጣብኝ ይሆናል የሚል ስጋት ይሰማዎታል? | 1. አዎ 0. አይሰማኝም |
| Q506 | ብዙውን ጊዜ ደስተኛ ነዎት? | 1. አዎ 0. አይደለሁም |
| Q507 | አብዛኛውን ጊዜ ምንም ማድረግ አልችልም የሚል ስሜት ይሰማዎታል? | 1. አዎ 0. አይሰማኝም |
| Q508 | ከቤት ወጥተው ስራዎችን ከማከናወን ይልቅ ቤት ውስጥ መቀመጥን ይመርጣሉ? | 1. አዎ 0. አልመርጥም |
| Q509 | ብዙ የማስታወስ ችግር አለብኝ ብለው ያስባሉ? | 1. አዎ 0. አላስብም |
| Q510 | አሁን ጥሩ ህይወት እየኖርኩ ነው ብለው ያስባሉ? | 1. አዎ 0. አላስብም |
| Q511 | አሁን ባሉበት ህይወት ለእራስዎ የሚሰጡት ዋጋ ትንሽ ነው ወይ? | 1. አዎ 0. አይደለም |
| Q512 | አሁን ያለኝ ጥንካሬ ጥሩ ነው ብለው ያስባሉ? | 1. አዎ 0. አላስብም |
| Q513 | አሁን ያሉበት ሁኔታ ተስፋ የሌለው እንደሆነ ይሰማዎታል? | 1. አዎ 0. አይሰማኝም |
| Q514 | ብዙ ሰዎች ከእርስዎ የተሻሉ እንደሆኑ ይሰማዎታል? | 1. አዎ 0. አይሰማኝም |

**ክፍል 7፡- ማህበራዊ ድጋፍን የሚዳስሱ ጥያቄዎች**

**መመሪያ**

ቀጥሎ ያሉት ጥያቄዎች በአካባቢዎ ካሉ ሰዎች (ቤተሰብ/ጓደኛ) እነማን እንደሚርዱዎት/እንደሚያግዙዎት የሚጥይቁ ናቸው። እያንዳንዱ ጥያቄ ሁለት ክፍል አለው።

የመጀመሪያው ክፍል እነማን እደሚያግዙዎ ስማቸውንና ዝምድናቸው/ከርስዎ ጋር ያላቸውን ግንኙነት እንዲዘረዝሩልን ሲሆን ሁልትኛው ክፍል ደግሞ ለመጀመሪያው ክፍል ለተገለጠው ድጋፍ ምን ያክል እንደረኩ የርካታ መጠንዎን ሚጠይቅ ነው።

ለመጀመሪያው ክፍል ጥያቄ መልስዎ “ማንም” ሚለው ቢሆንም እንኳን የርካታ መጠንዎን ግን ይገልፁልናል:: እባክዎ ሁሉንም ጥያቄዎች በሚችሉት መጠን ይመልሱ።

**ለምሳሌ:- እጅግ ሲበሳጩ ሊያፅናናዎትና ሊያረጋጋዎት ሚችል ሰው ማን ነው?**

ማንም( ) 2) አ.በ (ወንድም) 4) ረ.ሰ (ጓደኛ) 6) _____ 8)_________

1) ት. ፈ (የትዳር አጋር) 3) ረ.ግ( እህት) 5) ት.ነ (ዘመድ) 7) _____ 9)_________

**አጠቃላይ በነዚህ ሰዎች ድጋፍ ምን ያህል ረክተዋል?**

| መልስ | በጣም  ረክቻለሁ | በመጠኑ  ረክቻለሁ | ትንሽ  ረክቻለሁ | ትንሽ  አልረካሁም | በመጠኑ  አልረካሁም | በጣም  አልረካሁም |
| --- | --- | --- | --- | --- | --- | --- |
| የመልስ መለያ ኮድ | 6 | 5 | 4 | 3 | 2 | 1 |

| **ተ.ቁ** | **የጥያቄ ይዘት** | **የመልስ መለያ ኮድ** | | | | | | | | | | | | | | | | | | | | | | | |
| --- | --- | --- | --- | --- | --- | --- | --- | --- | --- | --- | --- | --- | --- | --- | --- | --- | --- | --- | --- | --- | --- | --- | --- | --- | --- |
| Q600 | በጭንቀት ሲዋጡ ከጭንቀትዎ እንዲወጡ ሊያደርጉዎት የሚችሉ እነማንን ሊጠቅሱልኝ ይችላሉ? | ማንም( ) 5) ____________  1) ________ 6) ____________  2) ________ 7)____________  3)_________ 8)____________  4) _________ 9)____________ | | | | | | | | | | | | | | | | | | | | | | | |
|  | አጠቃላይ በነዚህ ሰዎች ድጋፍ ምን ያህል ረክተዋል? | 6 | | | | | 5 | | | | 4 | | | 3 | | | | | | 2 | | | | | 1 |
| Q601 | ጭንቀት ወይም ውጥረት ሲያጋጥምዎ የበለጠ ዘና የሚል ስሜት እንዲሰማዎ ሊያደርግ የሚችል ሰው ሊጠቅሱልኝ  ይችላሉ? | ማንም( ) 5) ____________  1) ________ 6) ____________  2) ________ 7)____________  3)_________ 8)____________  4) _________ 9)____________ | | | | | | | | | | | | | | | | | | | | | | | |
|  | አጠቃላይ በነዚህ ሰዎች ድጋፍ ምን ያህል ረክተዋል? | 6 | | | | 5 | | | | 4 | | | | | | 3 | | | 2 | | | | | 1 | |
| Q602 | ያንተን ደካማም ጠንካራም  ማንነትህን ሙሉ በሙሉ ማን  ይቀበልሃል? | ማንም( ) 5) ____________  1) ________ 6) ____________  2) ________ 7)____________  3)_________ 8)____________  4) _________ 9)____________ | | | | | | | | | | | | | | | | | | | | | | | |
|  | አጠቃላይ በነዚህ ሰዎች ድጋፍ ምን ያህል ረክተዋል? | 6 | | | 5 | | | | | 4 | | | | | 3 | | | | 2 | | | | 1 | | |
| Q604 | ምንም ነገር ቢሆኑ/ቢያጋጥምዎት  ይንከባክበኛል/አብሮኝ ይሆናል/ ያስታምመኛል የሚሉትን ሰው ሊጠቅሱልኝ  ይችላሉ? | ማንም( ) 5) ____________  1) ________ 6) ____________  2) ________ 7)____________  3)_________ 8)____________  4) _________ 9)____________ | | | | | | | | | | | | | | | | | | | | | | | |
|  | አጠቃላይ በነዚህ ሰዎች ድጋፍ ምን ያህል ረክተዋል? | 6 | 5 | | | | | 4 | | | | | 3 | | | | | 2 | | | | 1 | | | |
| Q605 | በአጠቃላይ እጅግ አስቸጋሪ (ጭልም ያለ) ስሜት ሲሰማዎት ይደርሱልኛል፥  የተሻለ ስሜት እንዲሰማኝ  ያደርጋሉ ሚሏቸውን ሰዎች  ቢጥቅሱልኝ? | ማንም( ) 5) ____________  1) ________ 6) ____________  2) ________ 7)____________  3)_________ 8)____________  4) _________ 9)____________ | | | | | | | | | | | | | | | | | | | | | | | |
|  | አጠቃላይ በነዚህ ሰዎች ድጋፍ ምን ያህል ረክተዋል? | 6 | | 5 | | | | | 4 | | | 3 | | | | | 2 | | | | 1 | | | | |
| Q606 | እጅግ ሲናድደዱ ሊያፅናናዎት  ሊያረጋጋዎት ሚችል ሰው ማን  ነው? | ማንም( ) 5) ____________  1) ________ 6) ____________  2) ________ 7)____________  3)_________ 8)____________  4) _________ 9)____________ | | | | | | | | | | | | | | | | | | | | | | | |
|  | አጠቃላይ በነዚህ ሰዎች ድጋፍ ምን ያህል ረክተዋል? | 6 | | 5 | | | | | 4 | | | 3 | | | | | 2 | | | | 1 | | | | |

**ክፍል 8:- የዕለት ተዕለት እንቅስቃሴዎችን የተመለከቱ ጥያቄዎት**

የሚከተሉት ጥያቄዎች በዕለት ከዕለት ያለዎትን እንቅስቃሴዎች የተመለከቱ ናቸው፡፡ ለእያንዳደዱ ጥያቄዎች ከተሰጡት አማራጮች ውስጥ እኔን ይገልፁኛል የሚሉትን ይምረጡ፡፡

| **ተ.ቁ** | **የጥያቄ ይዘት** | **የመልስ መለያ ኮድ** |
| --- | --- | --- |
| Q700 | ሰውነትዎትን ሲታጠቡ፣ ክትትል፣መመሪያ ወይም የግል እርዳታ ይጠይቃሉ? | 1. አዎ 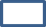 2.አልጠይቅም 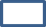 |
| Q701 | ልብስዎን በሚለብሱበት ጊዜ ክትትል፣መመሪያ ወይም የግል እርዳታ ይጠይቃሉ? | 1. አዎ 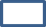 2. አልጠይቅም 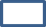 |
| Q702 | ሽንት ቤት በሚፀዳዱበት ጊዜ ክትትል፣መመሪያ ወይም የግል እርዳታ ይጠይቃሉ? | 1. አዎ 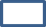 2. አልጠይቅም 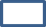 |
| Q703 | ከመኝታዎት ወይም ከአልጋዎ ወደ መቀመጫ ወንበር በሚንቀሳቀሱበት ጊዜ ክትትል፣መመሪያ ወይም የግል እርዳታ ይጠይቃሉ? | 1. አዎ 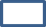 2. አልጠይቅም 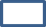 |
| Q704 | ሽንትዎ ወይም ሰገራዎ በሚመጣበት ወቅት መቆጣጠር ይችላሉ? | 1. አዎ 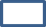 2. አልችልም 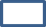 |
| Q705 | ምግብን ከእጅ መዳፍ ወደ አፋ ያለምንም እርዳታ ማንቀሳቀስ ይችላሉ? | 1. አዎ 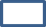 2. አልችልም 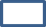 |

**ክፍል 9፡- ስለህይዎትዎ ጥራት/የኑሮ ሁኔታ፤ ጤንነትዎና ተዛማጅ ሁኔታዎችን የሚመለከቱ ጥያቄዎች**

**መመሪያ**

ይህ መጠይቅ ስለህይዎትዎ ጥራት/የኑሮ ሁኔታ፤ ጤንነትዎና ተዛማጅ ሁኔታዎች ነው። እባክዎ ሁሉንም ጥያቄዎች ለርስዎ ትክክል የሆነውን መልስ ይመልሱ። ለጥያቄዎቹ ሲመልሱ ባለፈው ሁለት ሳምንት ውስጥ ያለዎትን የንሮ ሁኔታ፥ ተስፋ፥ ደስታና ስጋት እያሰቡ ይሁን። ለእያንዳንዱ የምጠይቅወት ጥያቄ 5 የመልስ አማራጮች አሉት። እንደየጥያቄው አግባብነት ሊስተካከል ቢችልም በዋናነት መልሶቹ፥

**1.** የለም **3.** መካከልኛ **5.** እጅግ በጣም ከፍትኛ የሚሉ ናቸው።

**2.** በትንሽ **4.** በጣም ከፍትኛ

| **ተ.ቁ** | **የጥያቄ ይዘት** | በጣም  ዝቅተኛ | ዝቅተኛ | መካከለኛ | ከፍተኛ | በጣም ከፍተኛ |
| --- | --- | --- | --- | --- | --- | --- |
| QOL1 | የህይወትዎን ጥራት ደረጃ እንዴት ይገመግሙታል? | 1 | 2 | 3 | 4 | 5 |

| **ተ.ቁ** | **የጥያቄ ይዘት** | በጣም  የማያረካ | የማያረካ | መካከለኛ | የሚያረካ | በጣም  የሚያረካ |
| --- | --- | --- | --- | --- | --- | --- |
| QOL2 | በጤናዎ ምን ያህል ረክተዋል? | 1 | 2 | 3 | 4 | 5 |

**ቀጥሎ ላሉት ጥያቄዎች ባለፈው ሁለት ሳምንት ውስጥ ምን ያክል እንዳጋጠመው ይነግሩኛል**

| **ተ.ቁ** | **የጥያቄ ይዘት** | የለም | በትንሽ | መካከለኛ | በጣም  ብዙ | እጅግ  በጣም ብዙ |
| --- | --- | --- | --- | --- | --- | --- |
| QOL3 | የአካል ህመም ምክንያት ማድረግ ካለብዎት ነገር ምን ያህል እንዳስትጓጎልዎት ይሰማዎታል? | 1 | 2 | 3 | 4 | 5 |
| QOL4 | የዕለት ተለት እንቅስቃሴዎን ለመከወን ምን ያክል የህክምና እርዳታ ያስፈልግዎታል? | 1 | 2 | 3 | 4 | 5 |
| QOL5 | በህይወትዎ ምን ያክል ይደሰታሉ? | 1 | 2 | 3 | 4 | 5 |
| QOL6 | ህይወትዎ ምን ያክል ትርጉም አለው ብለው ይገምታሉ? | 1 | 2 | 3 | 4 | 5 |
| QOL7 | አእምሮዎን ለማሰባሰብ (ትኩረት ለማድረግ) ምን ያህል አቅም አለዎት? | 1 | 2 | 3 | 4 | 5 |
| QOL8 | በእልታዊ ህይዎትዎ ምን ያህል ደህንነት ይሰማዎታል? | 1 | 2 | 3 | 4 | 5 |
| QOL9 | ምን ያክል የአካል ጤንነት ይሰማዎታል? | 1 | 2 | 3 | 4 | 5 |

**ላልፉት ሁለት ሳምንታት ቀጥሎ ያሉትን ጥያቄዎች/ነግሮች ምን ያህል በተሟላ ሁኔታ እንደሰሩ ይመልሱልኛል**

| **ተ.ቁ** | **የጥያቄ ይዘት** | የለም | በትንሽ | መካከለኛ | በአብዛኛው | ሁሌም |
| --- | --- | --- | --- | --- | --- | --- |
| QOL10 | ለዕለት ተዕለት እንቅስቃሴዎ በቄ ጉልበት አለዎት? | 1 | 2 | 3 | 4 | 5 |
| QOL11 | የአካልዎን ገፅታ በፀጋ ተቀብለዋል? | 1 | 2 | 3 | 4 | 5 |
| QOL12 | ፍላጎትዎን ለማሟላት በቂ ገንዘብ አለዎት? | 1 | 2 | 3 | 4 | 5 |
| QOL13 | በየእለቱ የሚያስፈላዎትን መረጃ ያገኛሉ? | 1 | 2 | 3 | 4 | 5 |
| QOL14 | የመዝናኛ እንቅስቃሴዎችን የማግኘት አቅምዎ ምን ያክል ነው? | 1 | 2 | 3 | 4 | 5 |
| QOL15 | በአቅራቢያዎ ለመዘዋወር ምን ያክል አቅም አለዎት? | 1 | 2 | 3 | 4 | 5 |

**የሚከተሉት ጥያቄዎች ባለፉት ሁለት ሳምንታት ውስጥ ምን ያክል እርካታ፥ የደስታ፥ ወይም ጥሩ ስሜት እንዳደረብዎ የሚጠይቁ ናቸው**

| **ተ.ቁ** | **የጥያቄ ይዘት** | በጣም  የማያረካ | የማያረካ | መካከለኛ | የሚያረካ | በጣም  የሚያረካ |
| --- | --- | --- | --- | --- | --- | --- |
| QOL16 | በእንቅልፍዎ ምን ያክል ረክተዋል | 1 | 2 | 3 | 4 | 5 |
| QOL17 | ዕለታዊ የኑሮ እንቅስቃሴዎን  በመምራት ምን ያክል ረክተዋል? | 1 | 2 | 3 | 4 | 5 |
| QOL18 | በስራ ችሎታዎ ምን ያክል ረክተዋል? | 1 | 2 | 3 | 4 | 5 |
| QOL19 | በራስዎ ምን ያክል ረክተዋል? | 1 | 2 | 3 | 4 | 5 |
| QOL20 | ከሰዎች ጋር ባለዎት ግንኙነት ምን ያክል ረክተዋል? | 1 | 2 | 3 | 4 | 5 |
| QOL21 | በወሲባዊ ህይዎትዎ ምን ያክል ረክተዋል? | 1 | 2 | 3 | 4 | 5 |
| QOL22 | ከጓደኞችዎ በሚያገኙት ዕርዳታ ምን  ያክል ረክተዋል? | 1 | 2 | 3 | 4 | 5 |
| QOL23 | በመኖሪያ ቦታዎ ምን ያክል ረክተዋል? | 1 | 2 | 3 | 4 | 5 |
| QOL24 | ለጤና አግልግሎት አቅርቦት ያለዎት  እርካታ ምን ያህል ነው? | 1 | 2 | 3 | 4 | 5 |
| QOL25 | በመጓጓዣ በኩልስ? | 1 | 2 | 3 | 4 | 5 |

**ቀጥሎ ያለው ጥያቄ ባለፍው ሁለት ሳምንት ግዜ ውስጥ ምን ያህል በተደጋጋሚ እንዳጋጠመዎ ነው**

| **ተ.ቁ** | **የጥያቄ ይዘት** | በፍፁም | አልፎ  አልፎ | በተድጋጋሚ | ብዙ  ግዜ | ሁልግዜ |
| --- | --- | --- | --- | --- | --- | --- |
| QOL26 | አሉታዊ ስሜቶች፤ እንደመከፋት፥ ተስፋ  መቁረጥ፥ ጭንቀት ወይም ድብርት ምን ያህል  ተድጋግሞ ደርሶብዎታል? | 1 | 2 | 3 | 4 | 5 |
