# Supplementary material for: Measuring the effects of a nurse-led intervention on frailty status of older people living in the community in Ethiopia: A protocol for a quasi-experimental study
Source: PLoS One. 2024 Jan 19;19(1):e0296166. doi: 10.1371/journal.pone.0296166 (PMC10798498; doi:10.1371/journal.pone.0296166)
Supplement: Attachment — Submitted filename: Response to Reviewers_PLOS ONE_05.10.23.docx [file pone.0296166.s005.docx]

**Manuscript title:** Measuring the Effects of a Nurse-led Intervention on Frailty Status of Older People Living in the Community in Ethiopia: A Protocol for a Quasi-experimental Study.

**Manuscript ID:** PONE-D-23-00901

Dear Editor, we would like to thank you for the time to review our manuscript and for sharing supporting reference materials. In the current version of the manuscript, we included a figure that reflects the construct of the intervention. All the amendments and revisions made are highlighted in light yellow colour.

A. **Response to the academic editor**

| **Academic editor’s comment** | **Author’s response/explanation** |
| --- | --- |
| 1. Please ensure that your manuscript meets PLOS ONE's style requirements, including those for file naming. The PLOS ONE style templates can be found at   <https://journals.plos.org/plosone/s/file?id=wjVg/PLOSOne_formatting_sample_main_body.pdf> and  <https://journals.plos.org/plosone/s/file?id=ba62/PLOSOne_formatting_sample_title_authors_affiliations.pdf> | **Completed.**  Thank you for sharing the link to access the templates. We assure you that the manuscript meets PLOS ONE’S style requirements, including file naming. In the revised manuscript a figure has been included (see p. 7, line 170). |
| 2. Please include the following request for minor text overlap and do not ping with follow up: "We noticed you have some minor occurrence of overlapping text with the following previous publication(s), which needs to be addressed:- https://doi.org/10.3390/ijerph17186660. In your revision ensure you cite all your sources (including your own works), and quote or rephrase any duplicated text outside the methods section. Further consideration is dependent on these concerns being addressed." | **Completed**  For the requested minor textual overlaps, we ensured all the sources were cited.  We thought that we acknowledged the sources of information with appropriate citations. For example, information taken from the previously mentioned source/publication was cited as indicated using reference number 24. In the meantime, if still there are some concerns, we kindly wait for your advice. |
| 3. Please include the following request in the decision letter, and ping me with follow up." Please include a complete copy of PLOS’ questionnaire on inclusivity in global research in your revised manuscript. Our policy for research in this area aims to improve transparency in the reporting of research performed outside of researchers’ own country or community. The policy applies to researchers who have travelled to a different country to conduct research, research with Indigenous populations or their lands, and research on cultural artefacts. The questionnaire can also be requested at the journal’s discretion for any other submissions, even if these conditions are not met. Please find more information on the policy and a link to download a blank copy of the questionnaire here: https://journals.plos.org/plosone/s/best-practices-in-research-reporting. Please upload a completed version of your questionnaire as Supporting Information when you resubmit your manuscript. | **Completed**  The requested PLOS’ questionnaire on inclusivity in global research was completed and uploaded as ‘**Supporting Information’.** |
| 4. Please ask the authors to include an explanation for the retrospective CT registration and confirmation that all related CTs are registered, using send back in ITC desk notes. At RTC, please check the authors' response and ping me if the authors do not address this. | **Completed**  Update made to detail about clinical trial registration to ensure clarity about when the clinical trial was registered (see p.5, lines 115-119). |
| 5. Thank you for stating the following financial disclosure: "The funders had and will not have a role in study design, data collection and analysis, decision to publish, or preparation of the manuscript."At this time, please address the following queries:   a) Please clarify the sources of funding (financial or material support) for your study. List the grants or organizations that supported your study, including funding received from your institution.  b) State what role the funders took in the study. If the funders had no role in your study, please state: “The funders had no role in study design, data collection and analysis, decision to publish, or preparation of the manuscript.”  c) If any authors received a salary from any of your funders, please state which authors and which funders.  d) If you did not receive any funding for this study, please state: “The authors received no specific funding for this work.”  Please include your amended statements within your cover letter; we will change the online submission form on your behalf. | **Completed**  Regarding the financial disclosure, we want to assure you the following:   a. The source of funding for this study has been stated in the ‘funding statement’ section (see p.12, lines 284 to 287) b. The funders had no role in study design, data collection, and analysis, decision to publish, or preparation of the manuscript. c. Not applicable. d. The authors received no specific funding for this work except the student received a support Scholarship in the form of the University Postgraduate Award (UPA).     Amendments are included in the cover letter. |
| 6. In your Data Availability statement, you have not specified where the minimal data set underlying the results described in your manuscript can be found. PLOS defines a study's minimal data set as the underlying data used to reach the conclusions drawn in the manuscript and any additional data required to replicate the reported study findings in their entirety. All PLOS journals require that the minimal data set be made fully available. For more information about our data policy, please see <http://journals.plos.org/plosone/s/data-availability>. Upon re-submitting your revised manuscript, please upload your study’s minimal underlying data set as either Supporting Information files or to a stable, public repository and include the relevant URLs, DOIs, or accession numbers within your revised cover letter. For a list of acceptable repositories, please see http://journals.plos.org/plosone/s/data-availability#loc-recommended-repositories. Any potentially identifying patient information must be fully anonymized. Important: If there are ethical or legal restrictions to sharing your data publicly, please explain these restrictions in detail. Please see our guidelines for more information on what we consider unacceptable restrictions to publicly sharing data: http://journals.plos.org/plosone/s/data-availability#loc-unacceptable-data-access-restrictions. Note that it is not acceptable for the authors to be the sole named individuals responsible for ensuring data access.   We will update your Data Availability statement to reflect the information you provide in your cover letter. | **Completed**  The manuscript was edited to more clearly describe the minimal data set underlying the results described in the ‘Data Availability Statement’ (see p. 13, lines 304-305). |
| 7. We note that you have stated that you will provide repository information for your data at acceptance. Should your manuscript be accepted for publication, we will hold it until you provide the relevant accession numbers or DOIs necessary to access your data. If you wish to make changes to your Data Availability statement, please describe these changes in your cover letter and we will update your Data Availability statement to reflect the information you provide. | **Completed**  We thought that this had been done mistakenly during the submission process. We would like to publish our manuscript without holding for the provision of accession or DOI. Because we do not have any repository data at the acceptance. Information concerning this has been updated in the ‘Data Availability Statement’ section. |

B. **Response to Reviewer 1**

| **Reviewer’s comment** | **Author’s response/explanation** |
| --- | --- |
| You have clearly identified the rationale for the study. Developing an education package and delivering it is an ambitious project. I wish you well with this. | Thank you so much for your positive comments. |
| As a new program, I would think determining its acceptability and identifying any areas for modification are important outcomes. Yet I see no mention of acceptability/ adherence measures as outcomes. | **Completed**  The manuscript was amended with additional detail addressing this comment in the ‘Strengths and limitations’ section (see p. 9, line 228-231). |
| How will the program’s acceptability with those delivering it and those reviewing it be assessed? | **Completed**  The comments addressed in the revised manuscript (see p. 9, lines 228-232). |
| Will you measure adherence? If so, how? Meeting with the CHWs each month would be one measure. | **Completed**  The data collectors record completion of each of the six sessions by each of the participants. This will enable reporting on adherence to the intervention (see 9. 226-228). |
| Will you seek feedback on the training book? | **Completed**  Feedback from the participants was taken into consideration. Moreover, during the intervention period, there will be a fortnightly 5 to 10-minute follow-up phone call with participants to receive feedback about the education sessions and provide opportunistic counselling on the specific topics (see p. 10, line 231-232). |
| How do you determine compliance with the recommendations for exercise or diet? Or is this not possible? | **Completed**  The comment is addressed in the revised manuscript under the ‘strengths and limitations’ subheading (see p. 12, lines 278-282). |
| Please include the name of the instruments used to measure the primary outcome (frailty status) and the instruments used for the secondary measures. Table 3 lists and outlines the outcome measures (i.e. the score range, number of questions and a reference for the instrument). Maybe refer to this table earlier in the text in the methods, rather than later (in the data collection), or move text from this table into the methods section. | **Completed**  The names of the instruments used to measure the primary outcome (frailty status) has been included in the revised manuscript (see p.5, line 105). The names of the instruments used to measure the secondary outcomes are also included in the revised manuscript (see p. 5, lines 108 to 112)  We agree with the comments to refer to Table 3 earlier. To make the tables in their order, Table 3 in the previous manuscript has now been amended as Table 2 in the revised manuscript (see p. 5, line 112). |
| Line 123: as your sample calculation was an a priori analysis, change from future tense to past tense, i.e. sample size WILL be calculated, to sample size WAS calculated …. | **Completed**  Amendment made to sample size calculation (see p.6, line 131). |
| Line 154 – change singular to plural, i.e. study participant’s to study participants’ involvement. | **Completed**  On the ‘Authors access to study participant information and confidentiality’ amendments from study participant’s to study participants’ (see p. 7, line 163. |

**Response to Reviewer 2**

| **Reviewer’s comment** | **Author’s response/explanation** |
| --- | --- |
| Minor revisions:  1- Abstract: Grammatical error: Statistical  2- Line 126: Indicate the statistical testing method which achieves 95% power.  3- Line 220: Categorical data is typically summarized using both frequencies and percentages.  4- Line 220: State the statistical approach that will be used to test for normality of the data.  5- Since data will be collected at 3 time points, specifically indicate which outcomes and time points will be compared using Fisher’s exact tests, paired sample t-tests, and GLM with repeated measures ANVOVAs. | **Completed**   1. We could not find the typo for ‘statistical’. Please provide further detail so we can address this revision. 2. Regarding the statistical testing achieving 95%, a revision made (see p. 6, lines 133 to 134). 3. Regarding categorical data, a revision made (see p 10, line 238). 4. Regarding the statistical approach to test the normality of the data revision made (see p. 10 line 239). 5. Regarding both the primary and secondary outcomes measured at three points in times, revisions made. (see p. 10, lines 240-242). |

Thank you!
