# Supplementary material for: Measuring the effects of a nurse-led intervention on frailty status of older people living in the community in Ethiopia: A protocol for a quasi-experimental study
Source: PLoS One. 2024 Jan 19;19(1):e0296166. doi: 10.1371/journal.pone.0296166 (PMC10798498; doi:10.1371/journal.pone.0296166)
Supplement: Attachment — Submitted filename: Response to Reviewers_PLOS ONE_02.12.23.docx [file pone.0296166.s006.docx]

**Manuscript title:** Measuring the Effects of a Nurse-led Intervention on Frailty Status of Older People Living in the Community in Ethiopia: A Protocol for a Quasi-experimental Study.

**Manuscript ID:** PONE-D-23-00901

A. **Response to Reviewer 3**

| Academic editor’s comment | Author’s response/explanation |
| --- | --- |
| Minor comments: 1. Abstract: please specify the expected number of participants | **Completed**  Expected number of participants specified in abstract (see p. 2, line 38). |
| 2. Methods: I recommendend to add the number of falls among the secondary outcomes "number of falls in the 3 months prior to intervention, number of falls at 3 months, 6 months, and 12 months"). | **Completed**  Thank you for your genuine comments on this matter. To be clear the frequency of the data collection, will be immediately before the intervention (baseline) (T0), immediately post-intervention (T1), and at 12 weeks post-intervention (T2). Considering the literature and our study aim the number of falls is included in the baseline data collection. The falls history and frequency of falls of study participants in the last 12 months will be recorded at baseline. The details of our primary and secondary outcomes are to be measured at three different points in time (see p.10 lines 102 to 112). |
| 3. Methods: non-inclusion criteria, replace "cognitive impairment" by "major cognitive impairment" | **Completed**  The term "cognitive impairment" is replaced by "major cognitive impairment" (see p. 16, line 149). |
| 4. Methods: Student's t-test will be probably used to compare the level of frailty with categorical variables (sex, education, etc). Add Student's t-test.  I recommend this manuscript for publication. | **Completed**  Student's t-test will used to compare the level of frailty with categorical variables (see p. 24, line 274-275). |

B. **Response to Reviewer 4**

| **Reviewer’s comment** | **Author’s response/explanation** |
| --- | --- |
| Line 111.6, it is unclear why the study was registered PROSPECTIVELY 3 months after data collection. Should it be RETROSPECTIVELY as registration of the study happened after the start of data collection? | **Completed**  Thank you for your observation on this matter. This has been addressed in the revised manuscript (see p. 15, line 121). |
| Line 113-133, If the data is assumed to be normally distributed, why paired-t test is not used for sample size calculation? Also, please clarify the end point of the primary outcome for sample size calculation, either from T0 to T1 or T0 to T2. | Even though we used the Wilcoxon signed rank test we considered normal parent distribution methods to determine the sample using G-Power. The endpoint for the primary outcome for sample size calculation is from T0 to T2. |
| The author should state clearly which outcome measure is used for sample size calculation as the assumed effect size of 0.5 means it is assumed that the effect size of the intervention on the primary outcome of the study is 0.5, which should also be supported by evidence. | **Completed**  The primary outcome measure, frailty, was used for sample size calculation.  We set the effect size at 0.5 as it is often used as a practical rule of thumb, especially when there is no prior knowledge or specific expectations about the effect size. It provides a balance between detecting meaningful effects and ensuring a feasible sample size. A moderate effect size of 0.5 is considered large enough to be practically significant in many fields ^1^. |
| Line 137-144, it is unclear how the subjects will be recruited, either from the poster or CHW proactively to recruit subjects through the available list. More specifically, which sampling method will be used? | **Completed**  The reason for distributing a poster containing the aim of the study, eligibility criteria, and benefits of participating in the study was to make it readily available to anyone who need to take part in the study. However, we have made a revision so that it will be clearer how the subjects will be recruited. The study participants will be recruited using convenient sampling (see p. 16, lines 152 to 153, line 155 to 156). |
| The section Recruitment should place after the section on Eligibility. | **Completed**  A revision has been made based on the comment (see p. 16 line 142 and p.16 line 152). |
| Line 169, for the NLI program, the sessions can be distinct and interconnected (independent means they are unconnected) | **Completed**  A revision has been made based on the comment (see p. 17 line 172). |
| Line 226. Some descriptions of the outcome measures should be provided in the text, rather than provided in the hypothesis under Study design and hypothesis. Moreover, some of the outcomes were not touched such as recent appetite. | **Completed**  Thank you for your insights on this section.  In the study design and hypothesis section, we tried to amend the flow.  We believe that the outcome measurement descriptions are provided and explained at the end of the hypothesis and design section. Moreover, a brief description of each outcome measure, measurement tools and frequency of measurement has been indicated in Table 2.  ‘Recent appetite’ has been included as one of the outcome measures in the revised manuscript (see p. 10, lines 109). The measurement tool description for this outcome has been described in Table 2, section VI. |
| Acceptability of the program should also be measured from the perspectives of the participants on top of the interventionist (i.e., CHWs) | **Completed**  Acceptability of the program from the perspective of the participants will be recorded. This has been stated in the revised manuscript (see p. 23 lines 263 to 264). |
| Line 272-285, Please group the points for two subsections of strengths and limitations of the study. | Two subsections have been made in the revised version (see p. 25 line 304 and line 311). |

1.	Althubaiti, A. Sample size determination: A practical guide for health researchers. *J. Gen. Fam. Med.* **24**, 72–78 (2023).

Thank you!
